# Small‐Molecule Inhibitors Targeting Sterol 14α‐Demethylase (CYP51): Synthesis, Molecular Modelling and Evaluation Against *Candida albicans*

**DOI:** 10.1002/cmdc.202000250

**Published:** 2020-06-22

**Authors:** Faizah A. Binjubair, Josie E. Parker, Andrew G. Warrilow, Kalika Puri, Peter J. Braidley, Esra Tatar, Steven L. Kelly, Diane E. Kelly, Claire Simons

**Affiliations:** ^1^ School of Pharmacy & Pharmaceutical Sciences Cardiff University King Edward VII Avenue Cardiff CF10 3NB UK; ^2^ Centre for Cytochrome P450 Biodiversity Institute of Life Science Swansea University Swansea SA2 8PP UK; ^3^ Department of Pharmaceutical Chemistry Faculty of Pharmacy Marmara University 34668 Istanbul Turkey

**Keywords:** antifungal agents, azoles, *Candida albicans*, CYP51, drug design, molecular dynamics

## Abstract

Fungal infections are a global issue affecting over 150 million people worldwide annually, with 750 000 of these caused by invasive *Candida* infections. Azole drugs are the frontline treatment against fungal infections; however, resistance to current azole antifungals in *C. albicans* poses a threat to public health. Two series of novel azole derivatives, short and extended derivatives, have been designed, synthesised and investigated for CYP51 inhibitory activity, binding affinity and minimum inhibitory concentration (MIC) against *C. albicans* strains. The short derivatives were more potent against the *C. albicans* strains (e. g., MIC 2‐(4‐chlorophenyl)‐*N*‐(2,4‐dichlorobenzyl)‐3‐(1*H*‐imidazol‐1‐yl)propanamide (**5 f**) <0.03 μg/mL, *N*‐(4‐((4‐chlorophenyl)sulfonamido)benzyl)‐2‐phenyl‐3‐(1*H*‐1,2,4‐triazol‐1‐yl)propanamide (**12 c**), 1 μg/mL, fluconazole 0.125 μg/mL) but both displayed comparable enzyme binding and inhibition (**5 f** *K*_d_ 62±17 nM, IC_50_ 0.46 μM; **12 c** *K*_d_ 43±18 nM, IC_50_ 0.33 μM, fluconazole *K*_d_ 41±13 nM, IC_50_ 0.31 μM, posaconazole *K*_d_ 43±11 nM, IC_50_ 0.2 μM). The short series had poor selectivity for CaCYP51 over the human homologue, whereas the selectivity of the extended series, for example, compound **12 c**, was higher (21.5‐fold) than posaconazole (4.7‐fold) based on *K*_d_ values, although posaconazole was more selective (615‐fold) than **12 c** (461‐fold) based on IC_50_ values. Based on inhibitory activity and selectivity profile, the extended series are the better of the two series for further development.

## Graphical abstract

Two series of novel azole derivatives, short and long, have been designed, synthesised and investigated for CYP51 inhibitory activity, binding affinity and minimum inhibitory concentration against *C. albicans* strains. The short derivatives were more potent than the extended derivatives, but both displayed comparable enzyme binding and inhibition. Molecular modelling and biological and selectivity profiles provide useful SAR for further development.

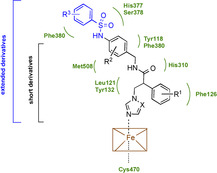

## Introduction

Fungal infections affect over 150 million people per annum, 750 000 of which are invasive infections by *Candida* sp. The incidence of life‐threatening systemic infections caused by *Candida albicans*, such as disseminated candidiasis and candidemia, has risen over the past several decades with mortality rates between 46–75 %.^1^ An estimated 2 million people annually contract oral candidiasis and a further 1.3 million oesophageal candidiasis.^2^ In addition, recurrent bouts of vulvovaginal candidosis (thrush) affect at least 75 million women annually,^3^ thus indicating unmet need for better treatments.

**Figure 1:**
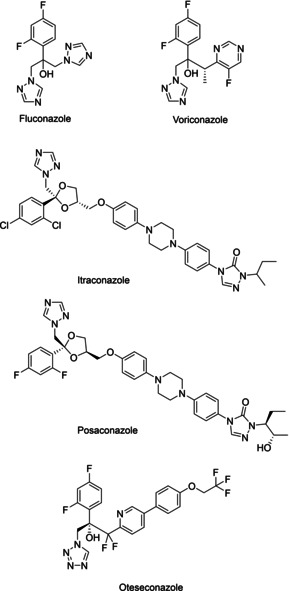
Chemical structures of azole antifungal agents.

Fluconazole has become the first‐line agent for treatment and prophylaxis against invasive candidiasis with voriconazole and itraconazole as alternative options (Figure 1).^4^, ^5^ The use of posaconazole has been limited mainly for oropharyngeal or oesophageal candidiasis and for prophylaxis in high‐risk patients owing to its erratic bioavailability and unpredictable trough plasma concentration.^6^ A new tetrazole‐based drug candidate VT‐1161 (oteseconazole) has been described and successfully completed Phase 2b clinical trials (Figure 1).^7^, ^8^ Azole antifungals inhibit sterol 14α‐demethylase (CYP51) resulting in depletion of ergosterol thereby affecting cell membrane integrity. However, resistance to current azole antifungals in *C. albicans* is becoming progressively more serious, posing a threat to public health^9^ and can be attributed to the prophylactic use of azole drugs and prolonged treatment regimens in the clinic.^10^, ^11^, ^12^

Azole resistance can arise through four main mechanisms. The affinity for *C. albicans* CYP51 may be reduced through point mutations leading to amino acid substitutions, the amount of CYP51 present may be increased due to upregulation of the gene and the efflux of azole from the cell may be increased due to the overexpression of transporters. In addition, it is possible for secondary mutations to occur which confer resistance such as *ERG3* null mutants that are resistant to azoles through a lack of *ERG3* activity leading to the production of 14‐methyl fecosterol when treated which is capable of supporting membrane function, thus the fungistatic effect of the accumulation of other 14‐methylated sterols is circumvented.^13^, ^14^, ^15^, ^16^

Over 140 CaCYP51 mutations have been described with single, double, triple and more recently quadruple mutations identified from clinical isolates.^17^, ^18^ However, the majority occur in drug‐sensitive strains and are therefore unlikely to contribute to azole resistance. CYP51 mutations associated with drug‐resistant strains primarily occur in the active site cavity (Y132H, Y132F, K143R, G307S and S405F), those that interact with the haem or are present in the Cys‐pocket which may affect the redox potential of the haem (K143R, G464S and R467K) and residues located on the β5‐hairpin (Y447H, G448E, G448V and G450E) that may affect interaction with the electron partner NADPH‐cytochrome P450 reductase (CPR) potentially affecting catalytic efficiency.^17^, ^18^, ^19^, ^20^

The enzymatic properties of *C. albicans* CYP51 (CaCYP51)^18^, ^21^ and the first X‐ray structures of this enzyme^22^ enable the computational study of CaCYP51 amino acid substitutions and the inhibitory effect of new azole compounds, whilst the X‐ray structures of human CYP51^23^ enable predictions of compound specificity for the fungal CYP51 over the human homologue to be made.

**Figure 2:**
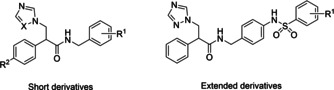
*N*‐Benzyl‐3‐(1*H*‐azol‐1‐yl/)‐2‐phenylpropanamide skeletons used in this study.

In this study *N*‐benzyl‐3‐(1*H*‐azol‐1‐yl/)‐2‐phenylpropanamides were used to explore binding and fit within CaCYP51 for both short and extended derivatives (Figure 2) establishing preliminary structure‐activity relationships (SARs), exploring additional binding interactions within the hydrophobic substrate access channel of CaCYP51 and evaluating inhibitory activity against CaCYP51 and MIC against *C. albicans* strains.

## Results and Discussion

### Chemistry

**Scheme 1:**
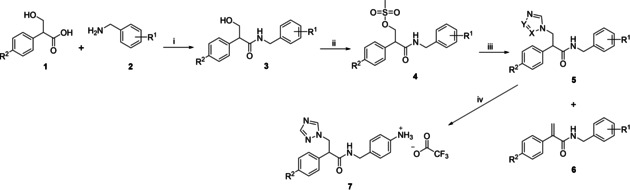
i) B(OCH_3_)_3_, CPME, 4 Å molecular sieves, 100 °C, o/n; ii) MsCl, Et_3_N, CH_2_Cl_2_, o/n; iii) a) K_2_CO_3_, imidazole/triazole/tetrazole, CH_3_CN, 45 °C, 1 h; b) 4, 70 °C, 1–24 h; iv) **5 l**, TFA, CH_2_Cl_2_.

*N*‐Benzyl‐3‐(1*H*‐imidazol/triazol‐1‐yl)‐2‐phenylpropanamides (**5**) were obtained by a three‐step synthetic route commencing with a trimethylborate (B(OMe)_3_) facilitated amidation (Scheme 1). A range of coupling reagents were investigated for the amidation step including carbonyldiimidazole, DCC, PyBOP, TBTU and T3P however either yields were unsatisfactory or, in the case of PyBOP, the product could not be isolated from the phosphine oxide by‐product.

The use of B(OCH_2_CF_3_)_3_ as the amidation reagent^24^ gave very good yields; however, we opted to use the more readily available and cheaper B(OMe)_3_ reagent which, when combined with activated 4 Å molecular sieves to absorb the generated water, gave satisfactory yields after solid‐phase work‐up described by Sabatini et al.^25^ (Table 1).

**Table 1:** Yields and melting points of amides (**3**) when using the B(OCH_3_)_3_ amidation method.

Cmpd	R^1^	R^2^	Yield [%]	Mp [°C]
**3 a**	H	H	98	108–110
**3 b**	4‐F	H	89	114–116
**3 c**	4‐Cl	H	85	135–136
**3 d**	4‐Cl	Cl	84	126–128
**3 e**	2,4‐diCl	H	100	78–80
**3 f**	2,4‐diCl	Cl	40	106–108
**3 g**	4‐CH3	H	100	96–98
**3 h**	4‐CF3	H	34	104–106
**3 i**	4‐OCH3	H	98	126–128
**3 j**	3,4‐diOCH3	H	83	116–118
**3 k**	NHBoc	H	79	118–120

The hydroxy group of **3** was then converted to the mesylate on reaction with methanesulfonyl chloride in the presence of Et_3_N, which after purification by column chromatography gave the mesylate products (**4**) in yields ranging from 35–70 %. The mesylates (**4**) were then reacted with either the potassium salt of imidazole or triazole or tetrazole (prepared in situ by treatment of imidazole/triazole/tetrazole with potassium carbonate in acetonitrile at 45 °C for 1 h) overnight at 70 °C. On reaction with the imidazolate anion a mixture of two products was generally obtained, the required imidazole (**5**) and the alkene elimination product (**6**; Scheme 1).

Only the 4‐chloro‐mesylate product (**4 c**) was reacted with all three azole anions; in the case of imidazole and triazole only the azole products (**5 c** and **5 k**, respectively) were formed, whereas on reaction with the tetrazole anion only the alkene elimination product (**6 c**) was formed, albeit in low yield (Table 2).

**Table 2:** The percentage of azole (**5**) and alkene (**6**) product and the ratio obtained.

Cmpd		R^1^	R^2^	Azole [%]	Cmpd	Alkene [%]	Ratio **5**/**6**
**Imidazole (X=Y=CH)**							
**5 a**		H	H	–	**6 a**	62	0 : 1
**5 b**		4‐F	H	34	**6 b**	39	1 : 1.15
**5 c**		4‐Cl	H	56	**6 c**	–	1:0
**5 d**		4‐Cl	4‐Cl	50^[a]^	**6 d**	22	2.3 : 1
**5 e**		2,4‐diCl	H	74	**6 e**	11	6.7 : 1
**5 f**		2,4‐diCl	4‐Cl	41	**6 f**	37	1:0.9
**5 g**		4‐CH_3_	H	5	**6 g**	59	1 : 11.5
**5 h**		4‐CF_3_	H	4	**6 h**	59	1 : 16
**5 i**		4‐OCH_3_	H	27	**6 i**	50	1 : 1.84
**5 j**		3,4‐diOCH_3_	H	51	**6 j**	23	2.2 : 1

**Triazole (X=N, Y=CH)**							
**5 k**		4‐Cl	H	77	**6 c**	–	1:0
**5 l**		NHBoc	H	84	**6 k**	9	9.3 : 1

**Tetrazole (X=Y=N)**							
**5 m**		4‐Cl	H	–	**6 c**	23	0 : 1

[a] The compound was then recrystallised from CH_3_CN to give 0.108 g, 26 %.

Electron‐withdrawing chloro substituents led to a preference for the azole substitution product (e. g., **5 c**, **5 d** and **5 e**), whereas the unsubstituted derivative or introduction of electron donating groups gave either exclusively or predominantly the elimination alkene product (e. g., **6 a**, **6 g**, **6 h** and **6 i**). The ratio of substitution to elimination product may be rationalised by the effects of these substituents on the acidity of the α‐proton, however this effect was not observed for the dimethoxy substituted compound, with the imidazole product (**5 j**) predominating.

For the extended derivatives only the triazole derivatives were prepared, as the triazole is the most commonly used azole in antifungals and generally triazoles have greater CYP selectivity than imidazoles owing to reduced basicity.^26^ The Boc protected triazole derivative **5 l** was prepared in order to then react, after Boc deprotection, with arylsulfonyl chlorides to extend the compounds to explore additional binding interactions/fit within the CYP51 active site.

The Boc protected triazole derivative **5 l** was successfully deprotected with trifluoroacetic acid in CH_2_Cl_2_ to give the triflate amine salt **7** in 82 % yield. Attempts to react the amine **7** with the arylsulfonyl chlorides (**8**, R=F or R=Cl), using either excess Et_3_N in CH_2_Cl_2_ or pyridine as base and solvent, were unsuccessful.

**Scheme 2:**
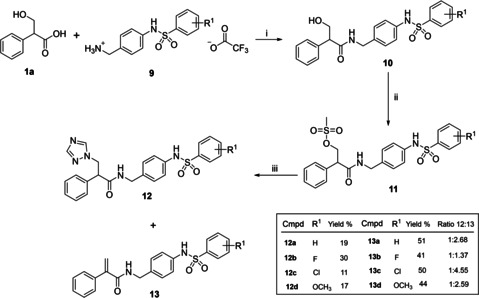
i) DCC, HOBt, EtOAc, 0 °C 30 min then RT o/n; ii) MsCl, Et_3_N, CH_2_Cl_2_, o/n; iii) a) K_2_CO_3_, triazole, CH_3_CN, 45 °C, 1 h; b) **11**, 70 °C, 1–24 h.

Therefore the aryl sulfonyl extension was introduced at an earlier stage with the (4‐(arylsulfonamido)phenyl)methanaminium 2,2,2‐trifluoroacetate derivatives (**9**) prepared as previously described^27^ and coupled with tropic acid (**1**). For these extended compounds low yields were obtained when using B(OMe)_3_ as the coupling reagent, however DCC/HOBt provided acceptable yields (67–87 %; Scheme 2). The resulting N‐(4‐(arylsulfonamido)benzyl)‐3‐hydroxy‐2‐phenylpropanamides (**10**) were then mesylated (**11**) and converted to the triazole derivatives (**12**) as previously described. Reaction with the triazolate anion resulted in a mixture of two products, the required triazole (**12**) and the alkene elimination product (**13**; Scheme 2), which was the major product for all derivatives.

### Antifungal susceptibility testing

The susceptibilities of the SC5314 and CA14*C. albicans* (azole sensitive) strains to all novel azole derivatives (**5**, **7** and **12**) were determined (Table 3) using the standardized CLSI M27‐S4 broth dilution method.^28^

**Table 3:** MIC values for compounds against *C. albicans* SC5314 and CA14 at 48 hours.

Cmpd				MIC [μg/mL] (μM)		clog*P* ^[a]^
	R^1^	R^2^	X	SC5314	CA14	
**5 b**	4‐F	H	CH	8 (24.7)	4 (12.4)	3.14
**5 c**	4‐Cl	H	CH	8 (23.5)	4 (11.8)	3.54
**5 d**	4‐Cl	Cl	CH	0.125 (0.33)	0.125 (0.33)	4.1
**5 e**	2,4‐diCl	H	CH	1 (2.67)	1 (2.67)	4.1
**5 f**	2,4‐diCl	Cl	CH	<0.03 (<0.07)	<0.03 (<0.07)	4.66
**5 g**	4‐CH_3_	H	CH	8 (25.1)	8 (25.1)	3.47
**5 h**	4‐CF_3_	H	CH	4 (10.7)	4 (10.7)	3.91
**5 i**	4‐OCH_3_	H	CH	8 (23.9)	8 (23.9)	2.86
**5 j**	3,4‐diOCH_3_	H	CH	>16 (>43.8)	>16 (>43.8)	2.73
**5 k**	4‐Cl	H	N	8 (23.5)	8 (23.5)	3.05
**7**	–	–	–	>16 (>36.8)	>16 (>36.8)	1.69
**12 a**	H	–	–	4 (8.67)	4 (8.67)	2.9
**12 b**	4‐F	–	–	4 (8.34)	2 (4.17)	3.06
**12 c**	4‐Cl	–	–	1 (2.02)	1 (2.02)	3.46
**12 d**	4‐OCH3	–	–	8 (16.3)	4 (8.14)	2.77
Fluc				0.125 (0.41)	0.125 (0.41)	0.86

[a] clog*P* was determined using Crippen's fragmentation.^29^

With the exception of the dimethoxy derivative (**5 j**), all the short derivatives displayed antifungal activity against both *C. albicans* wild‐type strains SC5314 and CA14. However the chloro derivatives were the most effective with **5 d** (R^1^=4‐Cl, R^2^=Cl) comparable with the standard fluconazole (MIC 0.125 μg/mL against both strains) and **5 f** (R^1^=2,4‐diCl, R^2^=Cl) more effective than fluconazole with MIC<0.03 μg/mL against both strains. The introduction of a chloro group at R^2^ was clearly beneficial (**5 c** vs **5 d** and **5 e** vs **5 f**) as was the presence of two chloro substituents at R^1^ (**5 c** vs **5 e** and **5 d** vs **5 f**). Generally, the more lipophilic (clog*P*) the short derivative (**5**) the better the MIC observed (Table 3). The MIC obtained was comparable whether the azole group was an imidazole (**5 c**) or a triazole ring (**5 k**). The free amine (**7**) was ineffective against both *C. albicans* strains (MIC>16 μg/mL), while for the extended derivatives (**12**) a trend was observed between MIC and clog*P* with the more lipophilic derivatives more effective at inhibiting fungal growth, for example: **12 c**, R^1^=4‐Cl, clog*P* 3.46, MIC 1 μg/mL compared with **12 a**, R^1^=H, clog*P* 2.9, MIC 4 μg/mL (Table 3).

### Inhibition of CaCYP51 (IC_50_ determination)

**Figure 3:**
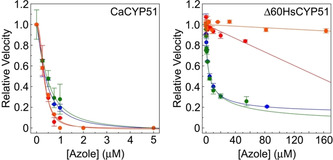
CYP51 azole inhibition profiles. Inhibition profiles for **5d** (•), **5f** (•), **12c** (•) and fluconazole (•) were determined in CYP51 reconstitution assays containing 1 μM CaCYP51 or 0.25 μM Δ60HsCYP51 with lanosterol as substrate. IC_50_ determinations were performed in duplicate with mean values shown along with standard deviations. Relative velocities of 1.00 correspond to an actual velocity of 0.911±0.141 min^−1^ for CaCYP51 and 8.16±2.84 min^−1^ for Δ60HsCYP51.

CYP51 reconstitution assays containing 1 μM CaCYP51 were performed as previously described.^30^ For CaCYP51, the concentrations of fluconazole and novel compounds (**5**, **7** and **12**) were varied from 0 to 10 μM. Exemplar IC_50_ profiles for fluconazole, **5 d**, **5 f** and **12 c** are shown in Figure 3. For a very tight binding inhibitor of CaCYP51 an IC_50_ value equal to half the enzyme concentration would be expected (∼0.5 μM). In the short series **5** the chloro derivatives **5 d** (R^1^=4‐Cl, R^2^=Cl) and **5 f** (R^1^=2,4‐diCl, R^2^=Cl) showed optimal inhibitory activity with IC_50_ 0.39 and 0.46 μM respectively compared with fluconazole IC_50_ 0.31 μM (Table 4). In addition, the 4‐methoxy derivative (**5 i**), showed promising inhibitory activity with IC_50_ of 0.91 μM. The free amine (**7**) showed weaker inhibitory activity (IC_50_ 1.96 μM). The extended derivatives (**12**) all showed very good inhibitory activity against CaCYP51 (IC_50_ 0.20–0.79 μM), with the halide derivatives **12 b** (R^1^=4‐F, IC_50_ 0.20 μM) and **12 c** (R^1^=4‐Cl, IC_50_ 0.33 μM) having similar activity to fluconazole (Table 4).

**Table 4:** Binding affinity (*K*_d_) and IC_50_ values for compounds against CaCYP51.

Cmpd				CaCYP51	*K*_d_
	R^1^	R^2^	X	IC_50_ [μM]^a^	[nM]
**5 b**	4‐F	H	CH	1.41	87±27
**5 c**	4‐Cl	H	CH	1.01	60±4
**5 d**	4‐Cl	Cl	CH	0.39	17±5
**5 e**	2,4‐diCl	H	CH	0.99	83±26
**5 f**	2,4‐diCl	Cl	CH	0.46	62±17
**5 g**	4‐CH_3_	H	CH	2.45	144±47
**5 h**	4‐CF_3_	H	CH	2.21	85±19
**5 i**	4‐OCH_3_	H	CH	0.91	55±10
**5 j**	3,4‐diOCH_3_	H	CH	6.15	–
**5 k**	4‐Cl	H	N	4.32	167±17
**7**	–	–	–	1.96	–
**12 a**	H	–	–	0.54	115±16
**12 b**	4‐F	–	–	0.20	55±27
**12 c**	4‐Cl	–	–	0.33	43±18
**12 d**	4‐OCH_3_	–	–	0.79	110±22
Fluc				0.31	41±13

[a] Curve‐fitted IC_50_ values (*v*_i_=*z*_o_+{*v*_o_/1+([I]/IC_50_)^n^ Mean *K*_d_ values of three replicates are shown along with associated standard deviations.

Previously IC_50_ values of 0.38 to 0.6, 0.2, 0.39 and 0.2 μM were obtained for fluconazole, voriconazole, itraconazole and posaconazole, respectively, using 1 μM CaCYP51 in CYP51 reconstitution assays.^18^, ^21^ Compounds **5 d** and **5 f** both had MIC values comparable with or lower than fluconazole against azole‐susceptible *C. albicans* strains and low IC_50_ values comparable with fluconazole (Table 4) against recombinant CaCYP51 indicating both compounds are good biochemical candidates for further study as antifungal agents. Compound **12 c**, whilst having a low IC_50_ value towards CaCYP51, had a MIC value that was eight times higher than that of fluconazole, suggesting a bioavailability problem within the *C. albicans* cells.

CYP51 reconstitution assays were also performed using 0.25 μM Δ60HsCYP51 in the presence of varying concentrations of compounds **5 d**, **5 f**, **12 c**, fluconazole, voriconazole and posaconazole and the inhibition profiles obtained are shown in Figure 3 and Figure S4 in the Supporting Information. The selectivity for CaCYP51 over the human homologue was relatively poor for compounds **5 d** and **5 f** with an only approximately eightfold difference in IC_50_ values (Table 5), exhibiting a similar selectivity as ketoconazole.^21^ This could limit the use of these two compounds as antifungal drugs. In contrast compound **12 c** exhibited high selectivity at 461‐fold (Table 5), which is similar to that observed with voriconazole (390‐fold) and better than the previously observed 175‐fold selectivity of itraconazole,^21^ thus suggesting this compound could be useful as an antifungal drug if uptake and bioavailability could be improved in *C. albicans* by further refinement of the chemical structure. However, compound **12 c** was less selective than posaconazole (615‐fold) and well behind the best compound fluconazole, which exhibited over 4000‐fold selectivity for CaCYP51 over the human homologue based on apparent IC_50_ values. By comparison the tetrazole VT‐1161 did not inhibit Δ60HsCYP51 activity at concentrations up to 50 μM and selectivity in excess of 2000‐fold was predicted.^31^

**Table 5:** Selectivity of compounds for CaCYP51 (Ca) over Δ60HsCYP51 (Hs) based on *K*_d_ and IC_50_.

Cmpd	*K*_d_ [nM]		Selectivity	IC_50_ [μM]		Selectivity
	Ca	Hs	Hs/Ca (fold)	Ca	Hs	Hs/Ca (fold)
**5 d**	17±5	45±13	2.6	0.39	3.37	8.6
**5 f**	62±17	74±13	1.2	0.46	3.73	8.1
**12 c**	43±18	923±509	21.5	0.33	152	461
fluconazole	41±13	38 460±4840	938	0.31	∼1327	4281
posaconazole	43±11	204±63	4.7	0.2^18^	123^[a]^	615
voriconazole	10±2^21^	2 290±120^21^	229	0.2^18^	78^[a]^	390
itraconazole	19±5^21^	92±7^21^	4.8	0.4^21^	70^21^	175
ketoconazole	12±3^21^	42±16^21^	3.5	0.5^21^	4.5^21^	9

Mean *K*_d_ values of three replicates are shown along with associated standard deviations. For IC_50_ determinations CYP51 assays contained 1 μM CaCYP51 (all azoles) or 0.25 μM Δ60HsCYP51 (**5 d**, **5 f**, **12 c** and fluconazole) or 0.4 μM Δ60HsCYP51 (posaconazole, voriconazole, itraconazole and ketoconazole). [a] See Figure S4.

### CaCYP51 ligand binding affinity

The absolute spectra of the purified CaCYP51 and Δ60HsCYP51 (c S1A) were typical for cytochromes P450 isolated primarily in the low spin state. Dithionite reduced carbon monoxide difference spectra (Figure S1B) produced the characteristic red‐shift of the Soret peak to ∼450 nm^32^ indicating the proteins were isolated in their native state and confirmed by the CYP51 reconstitution assays used to determine azole IC_50_ values.

**Figure 4:**
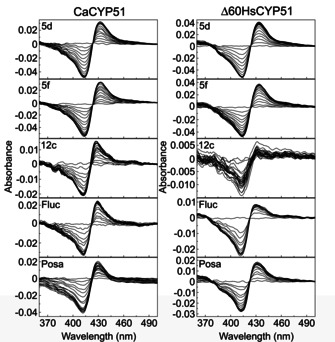
Azole binding difference spectra. Type II difference spectra obtained during the progressive titration of 3 μM native CaCYP51 and Δ60HsCYP51 with compounds **5 d**, **5 f**, **12 c**, fluconazole and posaconazole are shown. Each azole titration was performed in triplicate, although only one replicate is shown. The ligand saturation curves for these difference spectra are shown in Figure 5.

The novel short derivatives (**5**) and extended derivatives (**12**) with MIC<16 μg/mL and the standard, fluconazole, were then evaluated for CaCYP51 binding affinity (*K*_d_) by progressively titrating against CaCYP51. There were no signs of compound insolubility (visible or rising background absorbance) during the ligand titrations. Binding saturation curves were constructed from the absorbance difference (Δ*A*_peak‐trough_) derived from the difference spectra against the antifungal concentration for CaCYP51,^33^ enabling a direct comparison of binding type and affinity of novel compounds compared to fluconazole and posaconazole. Type II difference binding spectra were observed for all compounds titrated against CaCYP51 (Figure S2 and Figure 4) indicating the direct coordination of the imidazole or triazole nitrogen atom as the sixth axial ligand with the haem ferric ion of CaCYP51. Titration of **5 d**, **5 f**, **12 c**, and fluconazole with Δ60HsCYP51 also gave type II difference binding spectra, albeit the intensity (Δ*A*) obtained with **12 c** and fluconazole was smaller than the other two azoles (Figure 4).

**Figure 5:**
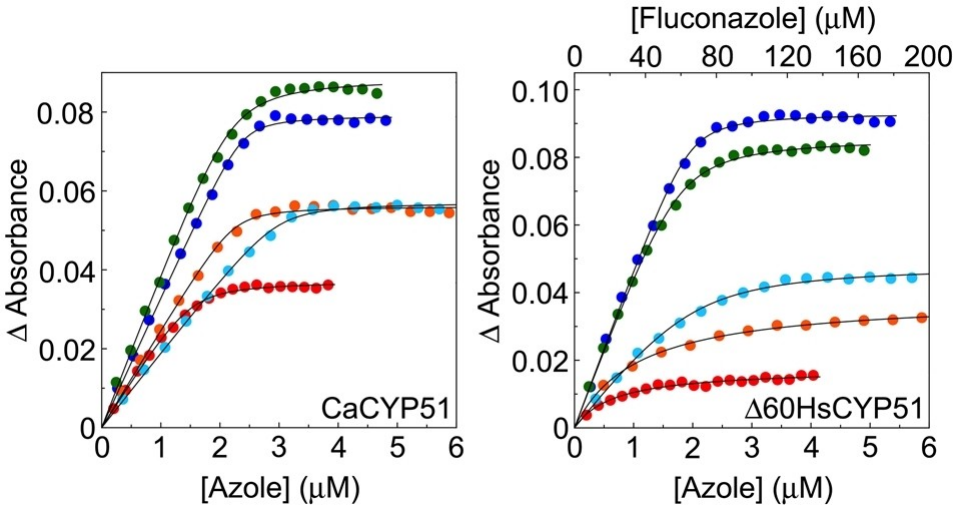
CYP51 azole saturation curves. Ligand binding saturation curves derived from the type II difference spectra in Figure 4 are shown for compounds **5d** (•), **5f** (•), **12c** (•), fluconazole (•) and posaconazole (•) with 3 μM native CYP51 protein. Each azole titration was performed in triplicate although only one replicate is shown.

The rearranged Morrison equation^34^ gave the best fit to the ligand saturation curves against CaCYP51 (Figure 5) indicating the selected compounds bound tightly to the purified CaCYP51 protein in free solution. Likewise the rearranged Morrison equation^34^ gave the best fit for binding **5 d** and **5 f** to Δ60HsCYP51, thus indicating tight binding in free solution (Figure 5). However, the Michaelis‐Menten equation best fit the binding of **12 c** and fluconazole to Δ60‐HsCYP51, indicating the binding was less tight (Figure 5). Previously Δ60HsCYP51 was shown to behave near identically to the full length HsCYP51 in terms of azole binding properties.^21^

In the short series tightest binding was observed for the chloro derivative **5 d** (R^1^=4‐Cl, R^2^=Cl, *K*_d_ 17±5 nM), which was slightly better than fluconazole (*K*_d_=41±13 nM). Good binding affinity was also observed for other chloro derivatives **5 c** (R^1^=4‐Cl, R^2^=H), **5 f** (R^1^=2,4‐diCl, R^2^=Cl) and the 4‐methoxy derivative **5 i** (R^1^=4‐OCH3, R^2^=H) with *K*_d_ of 60±4, 62±17 and 55±10 nM, respectively. In the extended series, the halide derivatives **12 b** (R^1^=4‐F, *K*_d_ 55±27 nM) and **12 c** (R^1^=4‐Cl, *K*_d_ 43±18 nM) showed good binding affinity comparable with fluconazole. The Morrison equation gave estimates of Kappi from the inhibition profiles with CaCYP51 of 160, 200 and 110 nM for compounds **5 d**, **5 f** and **12 c**, respectively, compared with 30 nM for fluconazole, confirming all four compounds bound tightly to CaCYP51.

Both **5 d** and **5 f** bound tightly to Δ60HsCYP51 in free solution with *K*_d_ values of 45 and 74 nM, respectively, whilst binding of **12 c** was less tight and more variable with a mean *K*_d_ of 923±509 nM (*n*=6; Table 5). Therefore, selectivity for CaCYP51 over the human homologue based on *K*_d_ values was poor for compounds **5 d** and **5 f** at 2.6‐ and 1.2‐fold, respectively. However, selectivity of compound **12 c** was higher at 21.5‐fold and was higher than posaconazole (4.7‐fold) and higher than previously observed with itraconazole and ketoconazole (Table 5). However, the selectivity of compound **12 c** based on *K*_d_ values was still substantially lower than those observed for voriconazole (229‐fold) and fluconazole (938‐fold).

### STEROL profiles

*C. albicans* strains (CA14 and SC5314) were grown in MOPS buffered RPMI in the presence of DMSO (untreated) or DMSO and antifungal (at half the MIC) for 18 hours, at 37 °C. Sterols were then extracted and analysed by GC‐MS and sterol profiles (% of the total sterol extracted) were determined (Table 6).

**Table 6:** Sterol composition (% of total sterols) of untreated and treated wild‐type *C. albicans* strains.

			Sterol composition [%]							
	Untreated (DMSO only)		Fluconazole 0.06 [μg/mL]		**5 d** 0.06 [μg/mL]		**5 f** 0.015 [μg/mL]		**12 c** 0.5 [μg/mL]	
	CA14	SC5314	CA14	SC5314	CA14	SC5314	CA14	SC5314	CA14	SC5314
ergosterol	76.9±3.8	77.2±3.8	63.8±3.4	72.8±3.3	7.8±6.5	2.7±2.3	4.3±0.5	9.1±5.5	3.5±1.3	24.9±5.5
diol^[a]^			0.2±0.4		8.2±6.0	17.6±7.1	7.5±4.6	11.7±1.4	9.2±1.2	7.2±5.2
lanosterol	3.8±0.6	5.2±5.0	19.6±1.5	14.5±1.7	40.4±2.2	41.0±5.5	43.6±1.8	38.6±3.6	43.6±1.5	34.5±2.9
eburicol		0.4±0.3	8.5±1.0	4.0±0.4	26.4±5.0	20.5±4.4	26.1±0.1	21.0±3.3	22.4±1.1	14.0±3.5
total 14α‐methylated sterols	3.8	5.6	31.4	22.2	90.0	95.8	77.2	89.3	93.3	70.6

[a] 14α‐Methyl ergosta‐8,24(28)‐dien‐3,6‐diol.

The sterol profiles showed an accumulation of 14α‐methylated sterols in both *C. albicans* strains treated with fluconazole, **5 d**, **5 f** and **12 c**. This confirms that the mechanism of action of **5 d**, **5 f** and **12 c**, like fluconazole, is the inhibition of sterol 14α‐demethylase (CYP51). The accumulation of 14α‐methylated sterols in the fungal membrane inhibits the growth of the *C. albicans*. In particular, the accumulation of 14α‐methyl ergosta‐8,24(28)‐dien‐3,6‐diol is believed to disrupt the fungal membrane in *Candida*, resulting in growth inhibition. Importantly, treatment with 0.06 μg/mL **5 d** resulted in a much higher accumulation of the “diol” sterol and concomitant depletion of ergosterol, indicating that **5 d** is more effective at inhibiting CYP51 activity than fluconazole.

### Molecular modelling

To investigate the binding modes of the short (**5**) and extended (**12**) azole derivatives, molecular dynamics simulations were run for 100 ns using the CaCYP51 crystal structure (PDB ID: 5FSA^22^) and representative short (**5 f**) and extended (**12 c**) azole derivative complexes, generated by using Molecular Operating Environment (MOE),^35^ and compared with fluconazole by using the Desmond programme of Maestro.^36^ All the compounds formed a coordination interaction between the imidazole or triazole N and the haem Fe.

**Figure 6:**
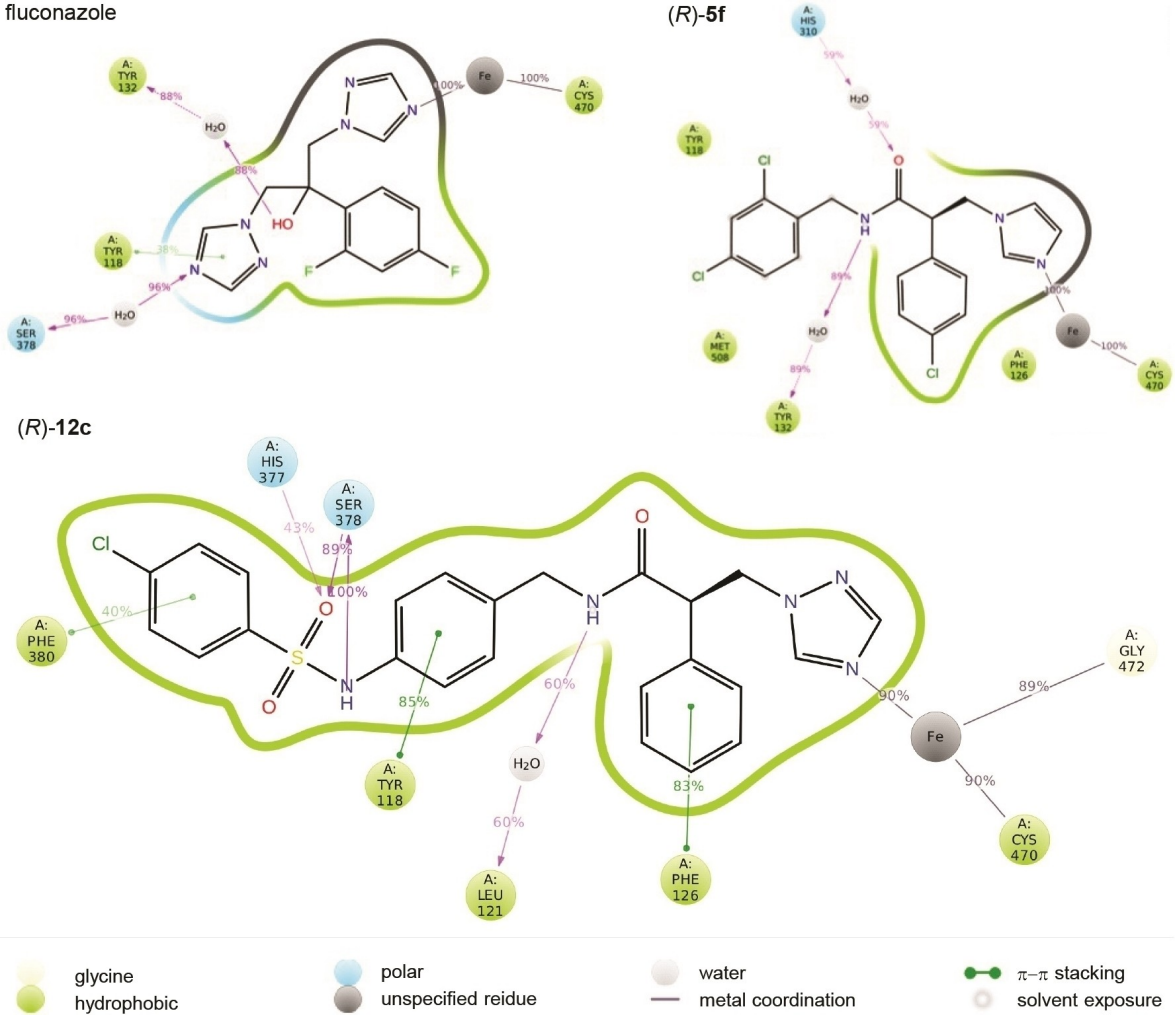
A schematic of detailed ligand atom interactions of fluconazole and representative (*R*)‐enantiomers of short derivative **5 f** and extended derivative **12 c** with the protein residues of wild‐type CaCYP51 active site. Interactions that occur more than 30.0 % of the simulation time in the selected trajectory (0 through 100 ns) are shown.

Different binding profiles were observed in the wild‐type CaCYP51 for the short derivatives (**5**) with a preference for the (*R*)‐enantiomers to form additional binding interactions within the ligand binding site, specifically water mediated H‐bonding interactions with His310 and Tyr132 and the amide heteroatoms, and hydrophobic interactions with Tyr118, Met508 and Phe126 (e. g., **5 f**, Figure 6), compared with fluconazole interactions that form π‐π stacking interactions with Tyr118 and triazole ring and water mediated H‐bonding interactions with Tyr132 and Ser378 and the hydroxy group and the heteroatom of the triazole ring respectively. The *S* enantiomers interacted primarily via hydrophic interactions, positioned to form π‐π stacking with Tyr118 and hydrophobic interactions with Phe126.

The *R* enantiomers of the extended compounds showed preferential binding with H‐bonding interactions between the sulfonamide group and Ser378 and His377, while the amide NH formed a water mediated interaction with Leu121. The three benzene rings formed π‐π stacking interactions with Phe126, Tyr118 and Phe380 (e. g., **12 c**, Figure 6). The *S* enantiomer formed H‐bonding interaction between the sulfonamide and Ser378 and the amide formed a water mediated interaction with Tyr132.

Several studies have shown that resistant *C. albicans* strains that have a double mutation CYP51 are considerably more resistant to fluconazole compared with a single mutation.^18^ Computational studies on the binding interaction of a representative mutant strain (Y132H+K143R) with the short (**5 f**) and extended (**12 c**) azole derivative complexes was performed and compared with fluconazole.

**Figure 7:**
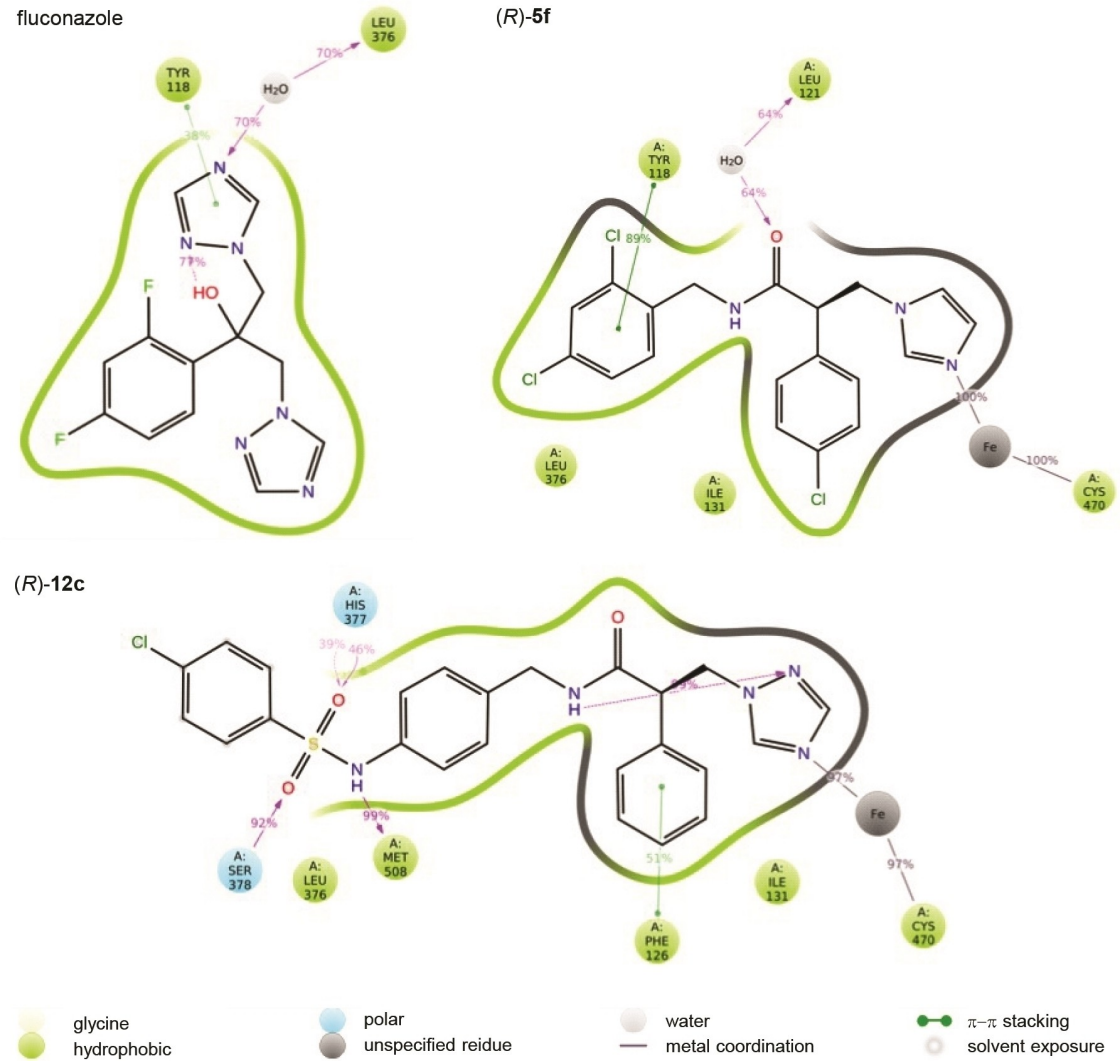
A schematic of detailed ligand atom interactions of fluconazole and representative (*R*)‐enantiomers of short derivative **5 f** and extended derivative **12 c** with the double mutant (Y132H+K143R) CaCYP51 protein. Interactions that occur more than 30.0 % of the simulation time in the selected trajectory (0–100 ns) are shown.

Of note in the binding of fluconazole with the double mutant (Y132H+K143R) CYP51 protein is the loss of haem binding and loss of the binding interaction with Tyr132, with the haem now forming a water mediated interaction with Leu376 and a π–π stacking bond with Tyr118 (Figure 7). (*R*)‐**5 f** retains the haem binding interaction through the imidazole nitrogen and although the interaction with Tyr132 and the NH of the amide is lost as a result of the Y132H mutation an interaction is still formed but through the O of the amide and Leu121 as well as a π–π stacking interaction with Tyr118 and hydrophobic interactions with Ile131 and Leu376. (*R*)‐**12 c** also retains the haem binding with the triazole N and importantly retains the H‐bonding interactions with His377, Ser378 and the amide as well as an additional H‐bonding interaction between NH of sulfonamide group and Met508, in the extension arm (Figure 7).

**Figure 8:**
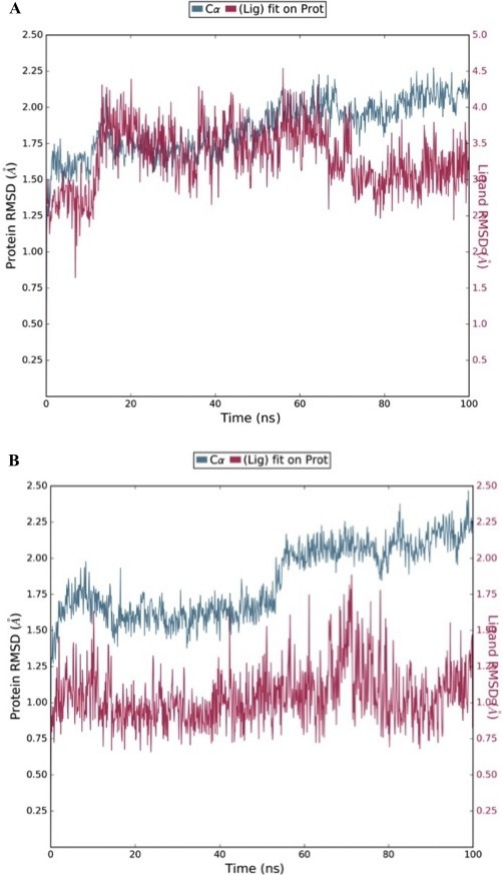
RMSD [Å] plot with respect to time in nanoseconds during 100 ns MD stimulation of A) (*R*)‐**5 f** and CYP51 (Y132H+K143R) complex and B) (*R*)‐**12 c** and CYP51(Y132H+K143R) complex.

For the (*R*)‐**5 f** and (*R*)‐**12 c** CYP51(Y132H+K143R) complexes, the mean Δ*G* (bind) was calculated^37^ from each frame from the point where the complex reached equilibrium to the final frame of the MD simulation with respect to RMSD (Figure 8).

The Δ*G* values indicate positioning within the mutant (Y132H +K143R) CaCYP51 was optimal with respect to fit of the two ligands. The (*R*)‐**12 c** complex, with Δ*G* of −69.86±5.46 kcal/mol would appear to have better binding affinity than (*R*)‐**5 f** with a Δ*G* of −46.35±3.73 kcal/mol; however, this Δ*G* calculation does not provide information with respect to haem binding and subsequently water mediated biotransformation. To determine this measurement of the distance from the azole N and the haem Fe before and after MD simulation needs to be determined.

In the wild‐type CYP51 complexes a relatively small shift is observed after MD stimulation (Table 7); however, a more significant shift is observed in the case of the double mutant (Y132H+K143R) CYP51 complexes (Table 7). For fluconazole a shift of 2.37 Å (pre‐MD) to 4.01 Å (post‐MD) reflects the loss of haem binding (Figure 7) and might explain the reduced effect of fluconazole against this mutant CaCYP51.^17^, ^18^ A less significant shift, compared with fluconazole, is observed with the (Y132H+K143R) CYP51 complexes of (*R*)‐**5 f** and (*R*)‐**12 c** from 2.39 and 2.33 Å (pre‐MD) to 3.08 and 3.11 Å (post‐MD), respectively (Table 7). This shift still allows binding with the haem, as seen in Figure 7, and the additional bonding interactions, in particular for (*R*)‐**12 c**, is reflected in the ΔG (bind) calculations (Figure 8).

**Table 7:** The distance between the N‐azole ring and the haem iron in the wild type/mutant CaCYP51 active site pre and post MD.

Compd	Wild type CaCYP51 [Å]		(Y132H+K143R) CaCYP51 [Å]	
	Pre‐MD	Post‐MD	Pre‐MD	Post‐MD
**5 f**	2.20	2.50	2.39	3.08
**12 c**	2.67	3.38	2.33	3.11
Fluc	2.74	2.28	2.37	4.10

**Figure 9:**
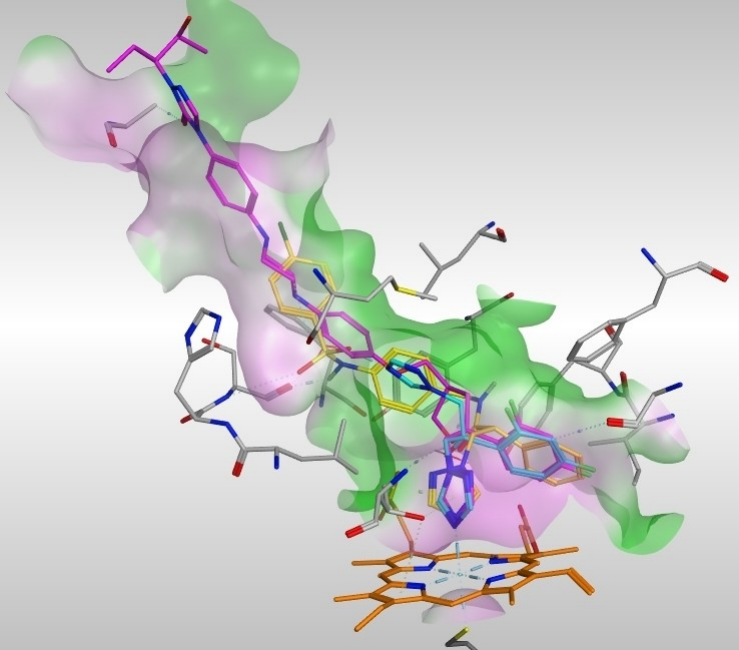
CaCYP51‐posaconazole (magenta) complex (PDB 5FSA) with posaconazole positioned along the hydrophobic active site cavity and above the haem (orange). Fluconazole (cyan) and extended derivative **12 c** (yellow) are aligned after MD simulations.

The placement of the azole derivatives in comparison with posaconazole and fluconazole in the CaCYP51 active site was visualised using MOE. Posaconazole (Figure 9, magenta) sits in a long hydrophobic channel with bonding primarily through multiple hydrophobic interactions with just one H‐bonding interaction observed between Ala61 and the carbonyl oxygen of the 1,2,4‐triazol‐5(4*H*)‐one ring. The optimal placement of fluconazole (Figure 9, cyan) was obtained after molecular dynamics simulations and, as expected, occupies a smaller area of the ligand binding channel compared with posaconazole with consequently fewer binding interactions. The short azole derivatives (**5**) mimic the positioning of fluconazole, whereas the more extended derivatives (e. g., **12 c**, yellow, Figure 9) sit between posaconazole and fluconazole, however importantly they form additional binding interactions with residues in the access channel compared with posaconazole, primarily through the sulfonamide group and benzene rings.

## Conclusion

Two series of novel azole derivatives, short and extended derivatives, have been designed, synthesised and investigated for CYP51 inhibitory activity, binding affinity and MIC against *C. albicans* strains. The short derivatives were more potent against the *C. albicans* strains (e. g., **5 f**, MIC<0.03 μg/mL, **12 c**, MIC 1 μg/mL, fluconazole 0.125 μg/mL) but both displayed comparable enzyme binding and inhibition (**5 f** *K*_d_ 62±17 nM, IC_50_ 0.46 μM; **12 c** *K*_d_ 43±18 nM, IC_50_ 0.33 μM, fluconazole *K*_d_ 41±13 nM, IC_50_ 0.31 μM). To determine whether any specific physicochemical factors might account for the difference in MIC, the physicochemical properties of the most promising prepared compounds and reference antifungal agents were calculated (Table 8). The clog*P* was determined using Crippen's fragmentation^29^ the molecular weight (MW), number of H‐bond acceptors (nON), H‐bond donors (nOHNH) rotatable bonds (nrot), along with the molecular volume (MV) and topological polar surface area (TPSA) were calculated using Molinspiration software.^38^ The number of violations (nviol) of Lipinskys is determined from the data presented (Table 8).

**Table 8:** Physiocochemical properties of selected derivatives and clinical antifungal agents.

Cmpd	MW	clog*P*	*n*_ON_/*n*_OHNH_	*n*_rot_	MV [Å^3^]	TPSA [Å^−2^]	*n*_viol_
**5 d**	374.264	4.1	4/1	6	316.96	46.92	0
**5 f**	408.709	4.66	4/1	9	414.51	46.92	0
**12 c**	495.981	3.46	8/2	9	414.51	105.98	0
fluconazole	306.271	0.87	7/1	5	248.96	81.66	0
voriconazole	349.311	2.59	6/1	5	285.11	76.73	0
itraconazole	*705.633*	*7.07*	*12*/1	11	607.90	104.73	3
posaconazole	*700.777*	*5.74*	*12*/1	12	623.35	115.72	3
oteseconazole	*527.394*	*5.2*	7/1	9	401.65	85.96	2

*n*_ON_=H‐bond acceptor; *n*_OHNH_=H‐bond donor; *n*_rot_=number of rotatable bonds; MV=molecular volume; TPSA=topological polar surface area; *n*_viol_=number of Lipinsky violations (violations are italicised).

The two series have a significantly increased clog*P*, although still within Lipinsky range, when compared with fluconazole but are similar to voriconazole and oteseconazole. The short series fits between fluconazole and voriconazole, while the extended series more closely resembles oteseconazole in all other physicochemical properties but, unlike the clinically described azoles, the extended series does not violate Lipinskys, showing more optimal drug like properties. There is a considerable range in all the physicochemical properties calculated for the clinically used potent azole antifungals, and the only clear difference observed with the two described series is for compound **12 c**, which has two H‐donors whereas all other compounds have one H‐donor.

The short series had poor selectivity for CaCYP51 over the human homologue based on *K*_d_ values, while the selectivity of the extended series, for example, compound **12 c**, was higher at 21.5‐fold and was higher than posaconazole (4.7‐fold) and higher than previously observed with itraconazole and ketoconazole, suggesting the extended series as optimal for further development. The extended series is better able to fill the binding site of CaCYP51 and forms additional binding interactions (H‐bonds with His377, Ser378, π–π stacking interactions with Phe126, Tyr118 and Phe380). Computational studies would suggest that the extended series (**12**) in particular may compensate for CYP51 mutations in resistant *C. albicans* strains through the formation of additional H‐bonding interactions, for example, with His277, Ser378 and Met508 in CaCYP51. The research described here will be developed further through computational studies to optimise binding interactions for CaCYP51 versus HsCYP51 and “design in” selectivity of extended azole inhibitors, while maintaining optimal drug like properties.

## Experimental Section

### Chemistry

All reagents and solvents were of general purpose or analytical grade and purchased from Sigma‐Aldrich, Fisher Scientific, Fluka, Alfa Aesar and Acros. Lanosterol and fluconazole were supplied by Sigma‐Aldrich. Ni^2+^‐NTA agarose affinity chromatography matrix was obtained from Qiagen. ^1^H and ^13^C NMR spectra were recorded with a Bruker Avance DPX500 spectrometer operating at 500 and 125 MHz, with Me_4_Si as internal standard. Mass spectra (HRMS) were determined by the Engineering and Physical Sciences Research Council National Mass Spectrometry Service Centre at Swansea University (Swansea, UK). Elemental analysis was performed by MEDAC Ltd (Chobham, Surrey, UK); HPLC (Method A, Cardiff University) was performed on a Shimadzu LC‐2030C Plus C18 Rapid Resolution 250×4.6 mm, 5 μm particle size using a 7–10 min gradient of water/methanol 5 : 95 (Method B, University of Bath) was performed on a Zorbax Eclipse Plus C18 Rapid Resolution 2.1×50 mm, 1.8 μm particle size using a 7.5 minute gradient method 5 : 95 water: methanol with 0.1 % formic acid as additive. Gradient column chromatography was performed with silica gel 60 (230‐400 mesh; Merck) and TLC was carried out on precoated silica plates (kiesel gel 60 F_254_, BDH). Compounds were visualised by illumination under UV light (254 nm) or by the use of vanillin stain followed by heating. Melting points were determined on an electrothermal instrument and are uncorrected. All solvents were dried prior to use and stored over 4 Å molecular sieves, under nitrogen. All the compounds were≥95 % pure.

**General procedure for the preparation of azoles (5 and 12) and alkene elimination products (6 and 13)**. To a stirred solution of azole (imidazole or triazole or tetrazole; 4 equiv) in dry CH_3_CN (2 mL/mmol of azole) was added potassium carbonate (4 equiv), and the mixture was heated for 1 h at 45 °C. After cooling to room temperature, mesylate (**4** or **11**; 1 equiv) was added and the reaction was heated at 70 °C for 4 h then stirred at room temperature overnight. The solvent was evaporated under vacuum and the residue was extracted with EtOAc (35 mL/mmol of mesylate), washed with brine (3×35 mL/mmol of mesylate) and water (3×35 mL/mmol of mesylate). The organic layer was dried (MgSO_4_) and evaporated under vacuum to give the crude product, which was purified by gradient column chromatography. Alkene (**6** or **13**) was eluted first with petroleum ether/EtOAc system, followed by the azole product (**5** or **12**) on changing the system to CH_2_Cl_2_/MeOH.

***N‐*** **Benzyl‐2‐phenylacrylamide (6 a, R^1^=R^2^=H)**. Prepared from 3‐(benzylamino)‐3‐oxo‐2‐phenylpropyl methanesulfonate (**4 a**; 0.48 g, 1.44 mmol) and purified by petroleum ether/EtOAc gradient column chromatography eluting with 70 : 30 *v*/*v*. Product was obtained as a white solid, yield 0.27 g (62 %); m.p. 78–80 °C; TLC (petroleum ether/EtOAc 1 : 1, *v*/*v*), *R*_f_=0.78; ^1^H NMR ([D_6_]DMSO): *δ* 8.74 (t, *J*=5.9 Hz, 1H, N*H*), 7.43 (m, 2H, Ar), 7.35 (m, 7H, Ar), 7.26 (m, 1H, Ar), 5.79 (s, 1H, C=C*Ha*Hb), 5.68 (s, 1H, C=CHa*Hb*), 4.40 (d, *J*=6.1 Hz, 2H, NHC*H_2_*); ^13^C NMR ([D_6_]DMSO): *δ* 168.70 (C, C=O), 145.65 (C, *C*=CH_2_), 140.07 (C, Ar), 137.18 (C, Ar), 128.79 (2×CH, Ar), 128.77 (2×CH, Ar), 128.57 (CH, Ar), 127.61 (2×CH, Ar), 127.52 (2×CH, Ar), 127.21 (CH, Ar), 118.17 (C=*C*H_2_), 42.81 (NH*C*H_2_); HRMS (ESI), *m/z* calcd for C_16_H_16_NO ([*M*+H]^+^), 238.1257; found: 238.1226.

***N*** **‐(4‐Fluorobenzyl)‐3‐(1*H*‐imidazol‐1‐yl)‐2‐phenylpropanamide (5 b, R^1^=4‐F, R^2^=H) and** ***N*** **‐(4‐fluorobenzyl)‐2‐phenylacrylamide (6 b, R^1^=4‐F, R^2^=H)**. Prepared from 3‐((4‐fluorobenzyl)amino)‐3‐oxo‐2‐phenylpropyl methanesulfonate (**4 b**; 0.70 g, 1.98 mmol) and purified by gradient column chromatography eluting the alkene (**6 b**) with petroleum ether/EtOAc 70 : 30 *v*/*v*, followed by the imidazole (**5 b**) with CH_2_Cl_2_/MeOH 90 : 10 *v*/*v. N‐(4‐Fluorobenzyl)‐3‐(1H‐imidazol‐1‐yl)‐2‐phenylpropanamide (**5 b**)* was obtained as a cream solid, yield 0.22 g (34 %); m.p. 100–102 °C; TLC (petroleum ether/EtOAc 1 : 1 *v*/*v*); *R*_f_=0.0; ^1^H NMR ([D_6_]DMSO): *δ* 8.61 (t, *J*=5.9 Hz, 1H, N*H*), 7.53 (brs, 1H, imid), 7.40 (d, *J*=7.05 Hz, 2H, Ar), 7.34 (t, *J*=7.4 Hz, 2H, Ar), 7.29 (t, *J*=7.2 Hz, 1H, Ar), 7.11 (brs, 1H, imid), 7.03 (m, 4H, Ar), 6.88 (brs, 1H, imid), 4.63 (dd, *J*=9.7, 13.4 Hz, 1H, CHC*Ha*Hb), 4.29 (dd, *J*=6.4, 15.3 Hz, 1H, NHC*Ha*Hb), 4.24 (dd, *J*=5.7, 13.4 Hz, CHCHa*Hb*), 4.10 (dd, *J*=5.5, 15.2 Hz, NHCHa*Hb*), 4.02 (dd, *J*=5.8, 9.6 Hz, C*H*CHaHb); ^13^C NMR ([D_6_]DMSO): *δ* 171.06 (C, C=O), 162.52 and 160.60 (C,C−F), 137.94 (C, Ar), 135.63 (C, Ar), 130.15 (CH, imid), 130.09 (CH, imid), 129.38 (CH, Ar), 129.32 (CH, Ar), 128.93 (2×CH, Ar), 128.27 (2×CH, Ar), 127.86 (CH, Ar), 115.66 (CH, imid), 115.40 (CH, Ar), 115.23 (CH, Ar), 53.33 (*C*HCH_2_imid), 48.88 (CH*C*H_2_imid), 41.80 (NH*C*H_2_); LRMS (ES, *m/z*): 324.15 [C_19_H_18_FN_3_O+H]^+^; HRMS (ESI), *m/z* calcd for C_19_H_19_FN_3_O ([*M*+H]^+^), 324.1507; found: 324.1507; HPLC (Method A): 98.0 %, *t*_R_=4.87 min. *N‐(4‐Fluorobenzyl)‐2‐phenylacrylamide (**6 b**)* was obtained as a white solid, yield 0.25 g (39 %); m.p. 108–110 °C; TLC (petroleum ether/EtOAc 1 : 1 *v*/*v*); *R*_f_=0.79; ^1^H NMR ([D_6_]DMSO): *δ* 8.74 (t, *J*=5.4 Hz, 1H, N*H*), 7.43 (d, *J*=7.7 Hz, 2H, Ar), 7.36 (m, 5H, Ar), 7.17 (t, *J*=8.9 Hz, 2H, Ar), 5.79 (s, 1H, C=C*Ha*Hb), 5.69 (s, 1H, C=CHa*Hb*), 4.38 (d, *J*=6.0 Hz, 2H, NHC*H_2_*); ^13^C NMR ([D_6_]DMSO): *δ* 168.68 (C, C=O), 162.60 and 160.67 (C, C−F), 145.60 (C, Ar), 137.16 (C, Ar), 136.27 (C, *C*=CH_2_), 129.67 (CH, Ar), 129.61 (CH, Ar), 128.79 (2×CH, Ar), 128.58 (CH, Ar), 127.53 (2×CH, Ar), 118.29 (C=*C*H_2_), 115.57 (CH, Ar), 115.40 (CH, Ar), 42.16 (NH*C*H_2_); elemental analysis calcd (%) for C_16_H_14_FNO (255.2911): C 75.28, H 5.53, N 5.48; found: C 75.34, H 5.32, N 5.55.

***N*** **‐(4‐Chlorobenzyl)‐3‐(1*H*‐imidazol‐1‐yl)‐2‐phenylpropanamide (5 c, R^1^=4‐Cl, R^2^=H)**. Prepared from 3‐((4‐chlorobenzyl)amino)‐3‐oxo‐2‐phenylpropyl methanesulfonate (**4 c**; 0.38 g, 1.0 mmol) and purified by gradient column chromatography eluting the imidazole (**5 c**) with CH_2_Cl_2_/MeOH 90 : 10 *v*/*v*. Product was obtained as a cream solid, yield 0.20 g (56 %); m.p. 136–138 °C; TLC (petroleum ether/EtOAc 1 : 1 *v*/*v*), *R*_f_=0.0; ^1^H NMR ([D_6_]DMSO): *δ* 8.67 (t, *J*=5.8 Hz, 1H, NH), 7.52 (s, 1H, imid), 7.40 (m, 2H, Ar), 7.35 (m, 2H, Ar), 7.29 (m, 3H, Ar), 7.11 (s, 1H, imid), 6.96 (d, *J*=8.2 Hz, 2H, Ar), 6.88 (s, 1H, imid), 4.63 (dd, *J*=9.9, 13.3 Hz, 1H, CHC*Ha*Hb), 4.30 (dd, *J*=6.4, 15.5 Hz, 1H, NHC*Ha*Hb), 4.23 (dd, *J*=5.6, 13.4 Hz, CHCHa*Hb*), 4.10 (dd, *J*=5.4, 15.5 Hz, NHCHa*Hb*), 4.03 (dd, *J*=5.7, 9.7 Hz, C*H*CHaHb); ^13^C NMR ([D_6_]DMSO): *δ* 171.2 (C, C=O), 138.5 (C, Ar), 137.9 (C, Ar), 131.7 (C, C−Cl), 129.2 (3×CH, Ar (2) and imid (1)), 129.0 (2×CH, Ar), 128.6 (3×CH, Ar (2) and imid (1)), 128.3 (2×CH, Ar), 127.9 (CH, Ar), 120.0 (CH, imid), 53.3 (*CH*CH_2_imid), 50.8 (CH*C*H_2_imid), 41.8 (NH*C*H_2_); elemental analysis calcd (%) for C_19_H_18_ClN_3_O**⋅**0.1H_2_O (341.6215): C 66.80, H 5.37, N 12.30; found: C 66.44, H 5.07, N 12.39; HPLC (Method A): 99.0 %, *t*_R_=4.94 min.

***N*** **‐(4‐Chlorobenzyl)‐2‐(4‐chlorophenyl)‐3(1*H*‐imidazol‐1‐yl)propanamide (5 d, R^1^=R^2^=4‐Cl) and** ***N*** **‐(4‐chlorobenzyl)‐2‐(4‐chlorophenyl)acrylamide (6 d, R^1^=R^2^=4‐Cl)**. Prepared from 3‐((4‐chlorobenzyl)amino)‐2‐(4‐chlorophenyl)‐3‐oxopropyl methanesulfonate (**4 d**; 0.44 g, 1.09 mmol) and purified by gradient column chromatography eluting the alkene (**6 d**) with petroleum ether/EtOAc 70 : 30 *v*/*v*, followed by the imidazole (**5 d**) with CH_2_Cl_2_/MeOH 90 : 10 *v*/*v*. Imidazole **5 d** was further purified by recrystallization from CH_3_CN. *N‐(4‐Chlorobenzyl)‐2‐(4‐chlorophenyl)‐3(1H‐imidazol‐1‐yl)propanamide (**5 d**)* was obtained as a brown solid, yield 0.11 g (26 %); m.p. 160–162 °C; TLC (petroleum ether/EtOAc 1 : 1 *v*/*v*); *R*_f_=0.0; ^1^H NMR ([D_6_]DMSO): *δ* 8.66 (t, *J*=5.9 Hz, 1H, N*H*), 7.50 (s, 1H, imid.), 7.40 (m, 4H, Ar), 7.29 (d, *J*=8.5 Hz, 2H, Ar), 7.09 (s, 1H, imid.), 6.98 (d, *J*=8.5 Hz, 2H, Ar), 6.86 (s, 1H, imid.), 4.59 (dd, *J*=9.4, 13.5 Hz, 1H, CHC*Ha*Hb), 4.29 (dd, *J*=6.4, 15.4 Hz, 1H, NHC*Ha*Hb), 4.24 (dd, *J*=6.1, 13.5 Hz, 1H, CHCHa*Hb*), 4.10 (dd, *J*=5.4, 15.5 Hz, 1H, NHCHa*Hb*), 4.05 (dd, *J*=6.1, 9.4 Hz, 1H, C*H*CHaHb); ^13^C NMR ([D_6_]DMSO): *δ* 170.78 (C, C=O), 138.41 (C, Ar), 138.06 (CH, imid), 136.78 (C, Ar), 132.59 (C, C−Cl), 131.75 (C, C−Cl), 130.16 (2×CH, Ar), 129.23 (2×CH, Ar), 128.92 (2×CH, Ar), 128.68 (CH, imid), 128.57 (2×CH, Ar), 119.95 (CH, imid), 52.59 (*C*HCH_2‐_imid), 48.66 (CH*C*H_2_imid), 41.85 (NH*C*H_2_); elemental analysis calcd (%) for C_19_H_17_Cl_2_N_3_O (374.2688): C 60.97, H 4.58, N 11.22; found: C 60.99, H 4.50, N 11.20; HPLC (Method A): 96.3 %, *t*_R_=2.53 min. *N‐(4‐Chlorobenzyl)‐2‐(4‐chlorophenyl)acrylamide (**6 d**)* was obtained as an off‐white solid, yield 0.09 g (22 %); m.p. 108–110 °C; TLC (petroleum ether/EtOAc 1 : 1 *v*/*v*); *R*_f_=0.73; ^1^H NMR ([D_6_]DMSO): *δ* 8.80 (t, *J*=5.9 Hz, 1H, N*H*), 7.45 (m, 4H, Ar), 7.41 (d, *J*=8.5 Hz, 2H, Ar), 7.33 (d, *J*=8.5 Hz, 2H, Ar), 5.84 (s, 1H, C=C*Ha*Hb), 5.76 (s, 1H, C=CHa*Hb*), 4.37 (d, *J*=6.1 Hz, 2H, NHC*H_2_*); ^13^C NMR ([D_6_]DMSO): *δ* 168.27 (C, C=O), 144.20 (C, *C*=CH_2_), 139.02 (C, Ar), 135.98 (C, C−Cl), 133.27 (C, C−Cl), 131.80 (C, Ar), 129.55 (2×CH, Ar), 129.46 (2×CH, Ar), 128.80 (2×CH, Ar), 128.74 (2×CH, Ar), 119.40 (C=*C*H_2_), 42.26 (NH*C*H_2_); elemental analysis calcd (%) for C_16_H_13_Cl_2_NO (306.1908): C 62.76, H 4.28, N 4.57; found: C 63.07, H 4.30, N 4.61.

***N*** **‐(2,4‐Dichlorobenzyl)‐3‐(1*H*‐imidazol‐1‐yl)‐2‐phenylpropanamide (5 e, R^1^=2,4‐Cl, R^2^=H) and** ***N*** **‐(2,4‐dichlorobenzyl)‐2‐phenylacrylamide (6 e, R^1^=2,4‐Cl, R^2^=H)**. Prepared from 3‐((2,4‐dichlorobenzyl)amino)‐3‐oxo‐2‐phenylpropyl methanesulfonate (**4 e**; 0.5 g, 1.24 mmol) and purified by gradient column chromatography eluting the alkene (**6 e**) with petroleum ether/EtOAc 80 : 20 *v*/*v*, followed by the imidazole (**5 e**) with CH_2_Cl_2_/MeOH 90 : 10 *v*/*v. N‐(2,4‐Dichlorobenzyl)‐3‐(1H‐imidazol‐1‐yl)‐2‐phenylpropanamide (**5 e**)* was obtained as a cream solid, yield 0.34 g (74 %); m.p. 55–57 °C; TLC (petroleum ether/EtOAc 1 : 1 *v*/*v*); *R*_f_=0.0; ^1^H NMR ([D_6_]DMSO): *δ* 8.68 (t, *J*=5.8 Hz, 1H, N*H*), 7.56 (d, *J*=2.1 Hz, 1H, Ar), 7.54 (brs, 1H, imid), 7.41 (d, *J*=7.2 Hz, 2H, Ar), 7.36 (t, *J*=7.4 Hz, 2H, Ar), 7.30 (t, *J*=7.2 Hz, 1H, Ar), 7.23 (dd, *J*=2.1, 8.4 Hz, 1H, Ar), 7.13 (brs, 1H, imid), 6.90 (brs, 1H, imid), 6.75 (d, *J*=8.4 Hz, 1H, Ar), 4.63 (dd, *J*=9.8, 13.4 Hz, 1H, CHC*Ha*Hb), 4.31 (dd, *J*=6.2, 16.0 Hz, 1H, NHC*Ha*Hb), 4.23 (dd, *J*=5.7, 13.4 Hz, 1H, CHCHa*Hb*), 4.16 (dd, *J*=5.4, 16.0 Hz, 1H, NHCHa*Hb*), 4.10 (dd, *J*=5.7, 9.8 Hz, 1H, C*H*CHaHb)); ^13^C NMR ([D_6_]DMSO): *δ* 171.32 (C, C=O), 137.72 (C, Ar), 135.52 (C, Ar), 133.34 (C, C−Cl), 132.63 (C, C−Cl), 130.12 (2×CH, Ar (1) and imid (1)), 128.97 (3×CH, Ar), 128.30 (2×CH, Ar), 127.94 (2×CH, Ar (1) and imid (1)), 127.51 (2×CH, Ar (1) and imid (1)), 53.21 (*C*HCH_2_imid), 48.86 (CH*C*H_2_imid), 40.01 (NH*C*H_2_); elemental analysis calcd (%) for C_19_H_17_Cl_2_N_3_O**⋅**0.2 H_2_O (377.87184): C 60.39, H 4.64, N 11.12; found: C 60.04, H 4.46, N 10.85; HPLC (Method A): 96.20 %, *t*_R_=3.20 min. *N‐(2,4‐Dichlorobenzyl)‐2‐phenylacrylamide (**6 e**)* was obtained as a cream solid, yield 0.05 g (11 %); m.p. 66–68 °C; TLC (petroleum ether/EtOAc 1 : 1 *v*/*v*), *R*_f_=0.85; ^1^H NMR ([D_6_]DMSO): *δ* 8.76 (t, *J*=5.8 Hz, 1H, N*H*), 7.63 (d, *J*=2.1 Hz, 1H, Ar), 7.47 (m, 3H, Ar), 7.34 (m, 5H, Ar), 5.83 (s, 1H, C=C*Ha*Hb), 5.75 (s, 1H, C=CHa*Hb*), 4.44 (d, *J*=5.9 Hz, 2H, NHC*H_2_*); ^13^C NMR ([D_6_]DMSO): *δ* 168.87 (C, C=O), 145.33 (C, *C*=CH_2_), 137.04 (C, Ar), 136.02 (C, C−Cl), 133.41 (C, C−Cl), 132.67 (C, Ar), 130.58 (CH, Ar), 129.06 (CH, Ar), 128.82 (2×CH, Ar), 128.63 (CH, Ar), 127.85 (CH, Ar), 127.58 (2×CH, Ar), 118.73 (C=*C*H_2_), 40.01 (NH*C*H_2_); LRMS (ESI, *m/z*): 308.0417 [C_16_H_13_^37^Cl_2_NO+H]^+^, 306.0447 [C_16_H_13_^35^Cl_2_NO+H]^+^, 158.98 [C_7_H_5_^35^Cl_2_]^+^; HRMS (ES), *m/z* calcd for C_16_H_14_^35^Cl_2_NO ([*M*+H]^+^), 306.0447; found: 306.0449; and calcd for C_16_H_14_^37^Cl_2_NO ([*M*+H]^+^), 308.0417; found: 308.0418.

**2‐(4‐Chlorophenyl)‐*N*‐(2,4‐dichlorobenzyl)‐3‐(1*H*‐imidazol‐1‐yl)propanamide (5 f, R^1^=2,4‐diCl, R^2^=Cl) and 2‐(4‐chlorophenyl)‐*N*‐(2,4‐dichlorobenzyl)acrylamide (6 f, R^1^=2,4‐diCl, R^2^=Cl)**. Prepared from 2‐(4‐chlorophenyl)‐3‐((2,4‐dichlorobenzyl)amino)‐3‐oxopropyl methanesulfonate (**4 f**; 0.54 g, 1.24 mmoL) and purified by gradient column chromatography eluting the alkene (**6 e**) with petroleum ether/EtOAc 60 : 40 *v*/*v*, followed by the imidazole (**5 e**) with CH_2_Cl_2_/MeOH 90 : 10 *v*/*v. 2‐(4‐Chlorophenyl)‐N‐(2,4‐dichlorobenzyl)‐3‐(1H‐imidazol‐1‐yl)propanamide (**5 f**)* was obtained as a white solid, yield 0.21 g (41 %); m.p. 148–150 °C; TLC (petroleum ether/EtOAc 1 : 1 *v*/*v*); *R*_f_=0.0; ^1^H NMR ([D_6_]DMSO): *δ* 8.69 (t, *J*=5.7 Hz, 1H, N*H*), 7.56 (d, *J*=2.2 Hz, 1H, Ar), 7.50 (brs, 1H, imid), 7.41 (m, 4H, Ar), 7.26 (dd, *J*=2.1, 8.3 Hz, 1H, Ar), 7.10 (brs, 1H, imid), 6.88 (brs, 1H, imid), 6.79 (d, *J*=8.4 Hz, 1H, Ar), 4.58 (dd, *J*=9.5, 13.4 Hz, 1H, CHC*Ha*Hb), 4.30 (dd, *J*=6.1, 15.9 Hz, 1H, NHC*Ha*Hb), 4.23 (dd, *J*=6.0, 13.5 Hz, 1H, CHCHa*Hb*), 4.16 (dd, *J*=5.5, 15.9 Hz, 1H, NHCHa*Hb*), 4.10 (dd, *J*=6.0, 9.5 Hz, 1H, C*H*CHaHb); ^13^C NMR ([D_6_]DMSO): *δ* 170.96 (C, C=O), 138.02 (CH, imid), 136.64 (C, Ar), 135.41 (C, Ar), 133.40 (C, C−Cl), 132,69 (C, C−Cl), 132.63 (C, C−Cl), 130.24 (CH, Ar), 130.19 (2×CH, Ar), 129.00 (CH, Ar), 128.94 (2×CH, Ar), 128.74 (CH, imid), 127.57 (CH, Ar), 119.92 (CH, imidazole), 52.47 (*C*HCH_2_), 48.68 (CH*C*H_2_), 40.58 (NH*C*H_2_); HRMS (ESI), *m/z* calcd for C_19_H_17_Cl_3_N_3_O ([*M*+H]^+^), 408.0438; found: 408.0432; HPLC (Method A): 99.6 %, *t*_R_=4.56 min. *2‐(4‐Chlorophenyl)‐N‐(2,4‐dichlorobenzyl)acrylamide (**6 f**)* was obtained as a white solid, yield 0.19 g (37 %); m.p. 104–106 °C; TLC (petroleum ether/EtOAc 1 : 1 *v*/*v*); *R*_f_=0.87; ^1^H NMR ([D_6_]DMSO): *δ* 8.81 (t, *J*=5.8 Hz, 1H, N*H*), 7.62 (d, *J*=2.1 Hz, 1H, Ar), 7.46 (dd, *J*=8.9, 16.3 Hz, 5H, Ar), 7.39 (d, *J*=8.3 Hz, 1H, Ar), 5.88 (s, 1H, C=C*Ha*Hb), 5.80 (s, 1H, C=CHa*Hb*), 4.43 (d, *J*=5.9 Hz, 2H, NHC*H_2_*); ^13^C NMR ([D_6_]DMSO): *δ* 168.43 (C, C=O), 143.97 (C, *C*=CH_2_), 135.91 (C, Ar), 135.88 (C, C−Cl), 133.41 (C, C−Cl), 133.30 (C, C−Cl), 132.69 (C, Ar), 130.60 (CH, Ar), 129.49 (2×CH, Ar), 129.07 (CH, Ar), 128.81 (2×CH, Ar), 127.86 (CH, Ar), 119.69 (C=*C*H_2_), 40.49 (NH*C*H_2_); HRMS (ESI), *m/z* calcd for C_16_H_12_Cl_3_NONa ([*M*+Na]^+^), 361.9882; found: 361.9880.

**3‐(1*H*‐Imidazol‐1‐yl)‐*N*‐(4‐methylbenzyl)‐2‐phenylpropanamide (5 g, R^1^=4‐CH_3_, R^2^=H) and** ***N*** **‐(4‐methylbenzyl)‐2‐phenylacrylamide (6 g, R^1^=4‐CH_3_, R^2^=H)**. Prepared from 3‐((4‐methylbenzyl)amino)‐3‐oxo‐2‐phenylpropyl methanesulfonate (**4 g**; 0.7 g, 2.0 mmol) and purified by gradient column chromatography eluting the alkene (**6 g**) with petroleum ether/EtOAc 70 : 30 *v*/*v*, followed by the imidazole (**5 g**) with CH_2_Cl_2_/MeOH 90 : 10 *v*/*v. 3‐(1H‐Imidazol‐1‐yl)‐N‐(4‐methylbenzyl)‐2‐phenylpropanamide (**5 g**)* was obtained as a cream solid, yield 0.03 g (5 %); m.p. 134–136 °C; TLC (petroleum ether/EtOAc 1 : 1 *v*/*v*), *R*_f_=0.0; ^1^H NMR ([D_6_]acetone): *δ* 7.72 (brs, 1H, N*H*), 7.52 (brs, 1H, imid.), 7.44 (d, *J*=7.0 Hz, 2H, Ar), 7.32 (m, 3H, Ar), 7.11 (brs, 1H, imid.), 7.04 (d, *J*=7.9 Hz, 2H, Ar), 6.95 (d, *J*=8.0 Hz, 2H, Ar), 6.91 (brs, 1H, imid.), 4.76 (dd, *J*=9.3, 13.6 Hz, 1H, CHC*Ha*Hb), 4.37 (dd, *J*=6.2, 14.8 Hz, 1H, NHC*Ha*Hb), 4.28 (dd, *J*=5.7, 13.6 Hz, 1H, CHCHa*Hb*), 4.23 (dd, *J*=5.6, 15.0 Hz, 1H, NHCHa*Hb*), 4.09 (dd, *J*=5.7, 9.3 Hz, 1H, C*H*CHaHb), 2.27 (s, 3H, C*H_3_*); ^13^C NMR ([D_6_]acetone): *δ* 170.50 (C, C=O), 137.77 (C, Ar), 136.18 (C, Ar), 136.01 (C, Ar), 128.80 (2×CH, Ar), 128.55 (3×CH, Ar (2) and imid (1)), 128.39 (CH, imid), 127.98 (3×CH, Ar (2) and imid (1)), 127.48 (2×CH, Ar), 127.18 (2×CH, Ar), 54.00 (*C*HCH_2_imid), 49.20 (CH*C*H_2_imid), 42.24 (NH*C*H_2_) 20.10 (*C*H_3_); HRMS (ESI), *m/z* calcd for C_20_H_22_N_3_O ([*M*+H]^+^), 320.1786; found: 320.1757; HPLC (Method A): 95.5 %, *t*_R_=2.49 min. *N‐(4‐Methylbenzyl)‐2‐phenylacrylamide (**6 g**)* was obtained as an off‐white solid, yield 0.38 g (59 %); m.p. 92–94 °C; TLC (petroleum ether/EtOAc 1 : 1 *v*/*v*), *R*_f_=0.78; ^1^H NMR ([D_6_]DMSO): *δ* 8.69 (t, *J*=6.0 Hz, 1H, N*H*), 7.43 (m, 2H, Ar), 7.35 (m, 3H, Ar), 7.20 (d, *J*=8.0 Hz, 2H, Ar), 7.15 (d, *J*=7.9 Hz, 2H, Ar), 5.78 (s, 1H, C=C*Ha*Hb), 5.66 (s, 1H, C=CHa*Hb*), 4.35 (d, *J*=6.1 Hz, 2H, NHC*H_2_*), 2.29 (s, 3H, C*H_3_*); ^13^C NMR ([D_6_]DMSO): *δ* 168.63 (C, C=O), 145.68 (C, *C*=CH_2_), 137.19 (C, Ar), 137.04 (C, Ar), 136.23 (C, Ar), 129.31 (2×CH, Ar),128.78 (2×CH, Ar), 128.55 (CH, Ar), 127.63 (2×CH, Ar), 127.50 (2×CH, Ar), 118.06 (C=*C*H_2_), 42.54 (NH*C*H_2_), 21.14 (*C*H_3_); elemental analysis calcd (%) for C_17_H_17_NO (251.3274): C 81.24, H 6.82, N 5.57; found: C 81.35, H 7.06, N 5.53.

**3‐(1*H*‐Imidazol‐1‐yl)‐2‐phenyl‐*N*‐(4‐(trifluoromethyl)benzyl)propanamide (5 h, R^1^=4‐CF_3_, R^2^=H) and 2‐phenyl‐*N*‐(4‐(trifluoromethyl)benzyl)acrylamide (6 h, R^1^=4‐CF_3_, R^2^=H)**. Prepared from 3‐oxo‐2‐phenyl‐3‐((4‐(trifluoromethyl)benzyl)amino)propyl methanesulfonate (**4 h**; 0.58 g, 1.44 mmol) and purified by gradient column chromatography eluting the alkene (**6 h**) with petroleum ether/EtOAc 70 : 30 *v*/*v*, followed by the imidazole (**5 h**) with CH_2_Cl_2_/MeOH 90 : 10 *v*/*v. 3‐(1H‐Imidazol‐1‐yl)‐N‐(4‐(trifluoromethyl)benzyl)‐2‐phenylpropanamide (**5 h**)* was obtained as a brown amorphous solid, yield 0.02 g (4 %); TLC (petroleum ether/EtOAc 1 : 1 *v*/*v*); *R*_f_=0.0; ^1^H NMR ([D_6_]acetone): *δ* 7.87 (brs, 1H, N*H*), 7.50 (m, 4H, Ar (3) and imid. (1)), 7.33 (m, 2H, Ar (1) and imid. (1)), 7.21 (m, 4H, Ar (3) and imid. (1)), 7.11 (m, 2H, Ar), 4.67 (dd, *J*=9.8, 12.8 Hz, 1H, CHC*Ha*Hb), 4.38 (dd, *J*=6.2, 15.6 Hz, 1H, NHC*Ha*Hb), 4.22 (d, *J*=5.3 Hz, 1H, CHCHa*Hb*), 4.20 (t, *J*=5.2 Hz, 1H, NHCHa*Hb*), 4.05 (dd, *J*=5.3, 8.9 Hz, 1H, C*H*CHaHb); ^13^C NMR ([D_6_]acetone): *δ* 170.85 (C, C=O), 143.94 (C, Ar), 137.47 (C, Ar), 128.64 (3×CH, Ar), 128.31 (CH, imid), 128.28 (C, Ar), 128.25 (CH, imid), 127.99 (3×CH, Ar), 127.63 (CH, Ar), 127.60 (2×CH, Ar), 127.49 (CH, imid), 125.07 & 125.04 (CF_3_), 53.82 (*C*HCH_2_imid), 49.89 (CH*C*H_2_imid), 42.03 (NH*C*H_2_); ^19^F NMR ([D_6_]acetone): *δ* −62.87; HRMS (ESI), *m/z* calcd for C_20_H_19_F_3_N_3_O ([*M*+H]^+^), 374.1506; found: 374.1475; HPLC (Method A): 97.7 %, *t*_R_=3.47 min. *2‐Phenyl‐N‐(4‐(trifluoromethyl)benzyl)acrylamide (**6 h**)* was obtained as an off‐white solid, yield 0.32 g (59 %); m.p. 88–90 °C; TLC (petroleum ether/EtOAc 1 : 1 *v*/*v*); *R*_f_=0.74; ^1^H NMR ([D_6_]DMSO): *δ* 8.83 (t, *J*=6.0 Hz, 1H, N*H*), 7.72 (d, *J*=8.1 Hz, 2H, Ar), 7.54 (d, *J*=8.0 Hz, 2H, Ar), 7.43 (d, *J*=6.8 Hz, 2H, Ar), 7.36 (m, 3H, Ar), 5.81 (s, 1H, C=C*Ha*Hb), 5.73 (s, 1H, C=CHa*Hb*), 4.48 (d, *J*=6.1 Hz, 2H, NHC*H_2_*); ^13^C NMR ([D_6_]DMSO): *δ* 168.81 (C, C=O), 145.46 (C, *=C=*), 144.97 (C, Ar), 137.10 (C, Ar), 128.81 (2×CH, Ar), 128.61 (CH, Ar), 128.30 (3×CH, Ar), 127.80 (C, Ar), 127.57 (3×CH, Ar), 125.69 & 125.66 (CF_3_), 118.60 (C=*C*H_2_), 42.55 (NH*C*H_2_); ^19^F NMR ([D_6_]DMSO): *δ* −60.78; elemental analysis calcd (%) for C_17_H_14_F_3_NO**⋅**0.1 H_2_O (307.10042): C 66.49, H 4.66, N 4.56; found: C 66.43, H 4.75, N 4.48.

**3‐(1*H*‐Imidazol‐1‐yl)‐*N*‐(4‐methoxybenzyl)‐2‐phenylpropanamide (5 i, R^1^=4‐OCH_3_, R^2^=H) and** ***N*** **‐(4‐methoxybenzyl)‐2‐phenylacrylamide (6 i, R^1^=4‐OCH_3_, R^2^=H)**. Prepared from 3‐((4‐methoxybenzyl)amino)‐3‐oxo‐2‐phenylpropyl methanesulfonate (**4 i**; 0.67 g, 1.85 mmol) and purified by gradient column chromatography eluting the alkene (**6 i**) with petroleum ether/EtOAc 60 : 40 *v*/*v*, followed by the imidazole (**5 i**) with CH_2_Cl_2_/MeOH 90 : 10 *v*/*v. 3‐(1H‐Imidazol‐1‐yl)‐N‐(4‐methoxybenzyl)‐2‐phenylpropanamide (**5 i**)* was obtained as a brown solid, yield 0.17 g (27 %); m.p. 102–104 °C; TLC (petroleum ether/EtOAc 1 : 1 *v*/*v*); *R*_f_=0.0; ^1^H NMR ([D_6_]DMSO): *δ* 8.52 (t, *J=*5.8 Hz, 1H, N*H*), 7.53 (brs, 1H, imid), 7.39 (d, *J*=7.2 Hz, 2H, Ar), 7.34 (t, *J*=7.4 Hz, 2H, Ar), 7.28 (t, *J*=7.2 Hz, 1H, Ar), 7.09 (brs, 1H, imid), 6.93 (d, *J*=8.6 Hz, 2H, Ar), 6.87 (brs, 1H, imid), 6.79 (d, *J*=8.6 Hz, 1H, Ar), 4.62 (dd, *J*=9.6, 13.4 Hz, 1H, CHC*Ha*Hb), 4.24 (ϕt, *J*=5.6, 4.3 Hz, 1H, NHC*Ha*Hb), 4.22 (t, *J*=5.8 Hz, 1H, CHCHa*Hb*), 4.06 (dd, *J*=5.4, 14.9 Hz, 1H, NHCHa*Hb*), 4.00 (dd, *J*=5.8, 9.5 Hz, 1H, C*H*CHaHb), 3.71 (s, 3H, C*H_3_*); ^13^C NMR ([D_6_]DMSO): *δ* 170.89 (C, C=O), 158.64 (C, *C*‐OCH_3_), 138.05 (C, Ar), 131.30 (C, Ar), 128.90 (2×CH, Ar (1) and imid (1)), 128.77 (2×CH, Ar (1) and imid (1)), 128.28 (2×CH, Ar (1) and imid (1)), 127.81 (CH, Ar), 114.05 (2×CH, Ar), 55.52 (O*C*H_3_), 53.31 (*C*HCH_2_imid), 48.91 (CH*C*H_2_imid), 41.99 (NH*C*H_2_); LRMS (ES+TOF, *m/z*): 336.17 [C_20_H_21_N_3_O_2_+H]^+^; HRMS (ES+TOF), *m/z* calcd for C_20_H_22_N_3_O_2_ ([*M*+H]^+^), 336.1712; found: 336.1715; HPLC (Method A): 95.9 %, *t*_R_=5.07 min. *N‐(4‐Methoxybenzyl)‐2‐phenylacrylamide (**6 i**)* was obtained as an off‐white solid, yield 0.31 g (50 %); m.p. 100–102 °C; TLC (petroleum ether/EtOAc 1 : 1 *v*/*v*); *R*_f_=0.64; ^1^H NMR ([D_6_]DMSO): *δ* 8.66 (t, *J*=5.9 Hz, 1H, N*H*), 7.43 (d, *J*=4.7 Hz, 2H, Ar), 7.36 (m, 3H, Ar), 7.25 (d, *J*=8.7 Hz, 2H, Ar), 6.91 (d, *J*=8.7 Hz, 2H, Ar), 5.77 (s, 1H, C=C*Ha*Hb), 5.65 (s, 1H, C=CHa*Hb*), 4.33 (d, *J*=6.1 Hz, 2H, NHC*H_2_*), 3.75 (s, 3H, C*H_3_*); ^13^C NMR ([D_6_]DMSO): *δ* 168.58 (C, C=O), 158.66 (C, *C*‐OCH_3_), 145.72 (C, *C*=CH_2_), 137.22 (C, Ar), 132.02 (C, Ar), 129.00 (2×CH, Ar), 128.78 (2×CH, Ar), 128.54 (CH, Ar), 127.50 (2×CH, Ar), 118.04 (C=*C*H_2_), 114.18 (2×CH, Ar), 55.53 (*C*H_3_), 42.27 (NH*C*H_2_); elemental analysis calcd (%) for C_17_H_17_NO_2_ (267.3268): C 76.38, H 6.41, N 5.24; found: C 76.35, H 6.30, N 5.13.

***N*** **‐(3,4‐Dimethoxybenzyl)‐3‐(1*H*‐imidazol‐1‐yl)‐2‐phenylpropanamide (5 j, R^1^=3,4‐diOCH_3_, R^2^=H) and** ***N*** **‐(3,4‐dimethoxybenzyl)‐2‐phenylacrylamide (6 j, R^1^=3,4‐diOCH_3_, R^2^=H)**. Prepared from 3‐((3,4‐dimethoxybenzyl)amino)‐3‐oxo‐2‐phenylpropyl methanesulfonate (**4 j**; 0.52 g, 1.30 mmol) and purified by gradient column chromatography eluting the alkene (**6 j**) with petroleum ether/EtOAc 60 : 40 *v*/*v*, followed by the imidazole (**5 j**) with CH_2_Cl_2_/MeOH 90 : 10 *v*/*v. N‐(3,4‐Dimethoxybenzyl)‐3‐(1H‐imidazol‐1‐yl)‐2‐phenylpropanamide (**5 j**)* was obtained as a pale yellow oil, yield 0.25 g (51 %); TLC (petroleum ether/EtOAc 1 : 1 *v*/*v*); *R*_f_=0.0; ^1^H NMR ([D_6_]DMSO): *δ* 8.55 (t, *J*=5.8 Hz, 1H, N*H*), 7.53 (brs, 1H, imid), 7.41 (d, *J*=7.2 Hz, 2H, Ar), 7.34 (t, *J*=7.4 Hz, 2H, Ar), 7.28 (t, *J*=7.3 Hz, 1H, Ar), 7.09 (brs, 1H, imid), 6.85 (brs, 1H, imid), 6.80 (d, *J*=8.2 Hz, 1H, Ar), 6.60 (d, *J*=1.8 Hz, 1H, Ar), 6.56 (dd, *J*=1.8, 8.2 Hz, 1H, Ar), 4.64 (dd, *J*=9.6, 13.5 Hz, 1H, CHC*Ha*Hb), 4.23 (dd, *J*=7.5, 13.5 Hz, 1H, CHCHa*Hb*), 4.16 (d, *J*=5.8 Hz, 2H, NHC*HaHb*), 4.01 (dd, *J*=5.7, 9.5 Hz, 1H, C*H*CHaHb), 3.70 (s, 3H, C*H_3_*), 3.58 (s, 3H, C*H_3_*); ^13^C NMR ([D_6_]DMSO): *δ* 170.93 (C, C=O), 149.10 (C, *C*OCH_3_), 148.17 (C, *C*OCH_3_), 138.16 (C, Ar), 131.88 (C, Ar), 128.93 (3×CH, Ar (2) and imid (1)), 128.52 (CH, imid), 128.27 (3×CH, Ar (2) and imid (1)), 127.81 (CH, Ar), 119.55 (CH, Ar), 112.05 (CH, Ar), 111.14 (CH, Ar), 56.02 (O*C*H_3_), 55.67 (O*C*H_3_), 53.35 (*C*HCH_2_imid), 48.80 (CH*C*H_2_imid), 42.21 (NH*C*H_2_); LRMS (ES+TOF, *m/z*): 366.18 [C_21_H_23_N_3_O_3_ + H]^+^; HRMS (ES+ TOF), *m/z* calcd for C_21_H_24_N_3_O_3_ ([*M*+H]^+^), 366.1818; found: 366.1826; HPLC (Method A): 99.9 %, *t*_R_=4.81 min. *N‐(3,4‐Dimethoxybenzyl)‐2‐phenylacrylamide (**6 j**)* was obtained as white solid, yield 0.09 g (23 %); m.p. 102–104 °C; TLC (petroleum ether/EtOAc 1 : 1 *v*/*v*); *R*_f_=0.64; ^1^H NMR ([D_6_]DMSO): *δ* 8.66 (t, *J*=6.0 Hz, 1H, N*H*), 7.44 (m, 2H, Ar), 7.36 (m, 3H, Ar), 6.92 (m, 2H, Ar), 6.84 (dd, *J*=1.9, 8.2 Hz, 1H, Ar), 5.78 (s, 1H, C=C*Ha*Hb), 5.65 (s, 1H, C=CHa*Hb*), 4.33 (d, *J*=6.1 Hz, 2H, NHC*H_2_*), 3.75 (s, 3H, C*H_3_*), 3.74 (s, 3H, C*H_3_*); ^13^C NMR ([D_6_]DMSO): *δ* 168.68 (C, C=O), 149.13 (C, *C*‐OCH_3_), 148.21 (C, *C*‐OCH_3_), 145.75 (C, *C*=CH_2_), 137.19 (C, Ar), 132.52 (C, Ar), 128.79 (2×CH, Ar), 128.57 (CH, Ar), 127.45 (2×CH, Ar), 119.74 (CH, Ar), 117.87 (C=*C*H_2_), 112.24 (CH, Ar), 111.69 (CH, Ar), 56.05 (*C*H_3_), 55.86 (*C*H_3_), 42.54 (NH*C*H_2_); elemental analysis calcd (%) for C_18_H_19_NO_3_ (299.3530): C 72.71, H 6.44, N 4.71; found: C 72.59, H 6.46, N 4.82.

***N*** **‐(4‐Chlorobenzyl)‐2‐phenyl‐3‐(1*H*‐1,2,4‐triazol‐1‐yl)propanamide (5 k, R^1^=4‐Cl, R^2^=H)**. Prepared from 3‐((4‐chlorobenzyl)amino)‐3‐oxo‐2‐phenylpropyl methanesulfonate (**4 c**; 0.59 g, 1.61 mmol) and purified by gradient column chromatography eluting the triazole (**5 k**) with petroleum ether/EtOAc 10 : 90 *v*/*v*. Product was obtained as a white solid, yield 0.22 g (40 %); m.p. 113–115 °C; TLC (petroleum ether/EtOAc 1 : 1 *v*/*v*); *R*_f_=0.0; ^1^H NMR ([D_6_]DMSO): *δ* 8.72 (t, *J*=6.0 Hz, 1H, N*H*), 8.32 (s, 1H, triaz), 7.99 (s, 1H, triaz), 7.20 (m, 5H, Ar), 7.28 (d, *J*=8.4 Hz, 2H, Ar), 6.97 (d, *J*=8.5 Hz, 2H, Ar), 4.83 (dd, *J*=9.2, 13.8 Hz, 1H, CHC*Ha*Hb), 4.44 (dd, *J*=6.5, 13.5 Hz, 1H, CHCHa*Hb*), 4.27 (m, 2H, C*H*CHaHb+NHC*Ha*Hb), 4.08 (dd, *J*=5.5, 15.6 Hz, NHCHa*Hb*); ^13^C NMR ([D_6_]DMSO): *δ* 170.74 (C, C=O), 151.99 (CH, triaz), 145.08 (CH, triaz), 138.58 (C, Ar), 137.94 (C, C−Cl), 137.47 (C, C−Cl), 131.68 (C, Ar), 129.9.36 (CH, Ar), 129.07 (2×CH, Ar), 129.02 (2×CH, Ar), 128.78 (CH, Ar), 128.54 (2×CH, Ar), 128.23 (CH, Ar), 51.56 (*CH*CH_2_triaz), 51.10 (CH*C*H_2_triaz), 41.72 (NH*C*H_2_); elemental analysis calcd (%) for C_18_H_17_ClN_4_O (340.8115): C 63.44; H, 5.03; N, 16.43 ; found: C 63.22; H, 5.01; N, 16.33; HPLC (Method A): 99.7 %, *t*_R_=4.91 min.

***tert‐*** **Butyl (4‐((2‐phenyl‐3‐(1*H*‐1,2,4‐triazol‐1‐yl)propanamido)methyl)phenyl)carbamate (5 l, R^1^=NHBoc, R^2^=H) and** ***tert*** **‐butyl (4‐((2‐phenylacrylamido)methyl)phenyl)carbamate (6 l, R^1^=NHBoc, R^2^=H)**. Prepared from 3‐((4‐((*tert*‐butoxycarbonyl)amino)benzyl)amino)‐3‐oxo‐2‐phenylpropyl methanesulfonate (**4 k**; 0.62 g, 1.39 mmol) and purified by gradient column chromatography eluting the alkene (**6 l**) with petroleum ether/EtOAc 60 : 40 *v*/*v*, followed by the imidazole (**5 l**) with CH_2_Cl_2_/MeOH 90 : 10 *v*/*v. tert‐Butyl (4‐((2‐phenyl‐3‐(1H‐1,2,4‐triazol‐1‐yl)propanamido)methyl)phenyl)carbamate (**5 l**)* was obtained as a pale yellow oil, yield 0.49 g (84 %); TLC (petroleum ether/EtOAc 1 : 1 *v*/*v*); *R*_f_=0.0; ^1^H NMR (CDCl_3_): *δ* 7.80 (s, 1H, triazole), 7.76 (s, 1H, triazole), 7.18 (m, 7H, Ar), 6.81 (d, *J*=8.5 Hz, 2H, Ar), 6.61 (s, 1H, N*H*), 6.10 (t, *J*=5.6 Hz, 1H, N*H*CHaHb), 4.83 (dd, *J*=8.7, 13.6 Hz, 1H, CHC*Ha*Hb), 4.23 (ddd, *J*=6.1, 14.7, 21.0 Hz, 2H, CHCHa*Hb* and NHC*Ha*Hb), 4.11 (dd, *J*=5.5, 9.2, 14.8 Hz, 1H, NHCHa*Hb*), 3.97 (dd, *J*=6.2, 8.7 Hz, 1H, C*H*CHaHb), 1.39 (s, 9H, C(C*H_3_*)_*3*_); ^13^C NMR (CDCl_3_): *δ* 170.26 (C, C=O), 152.79 (C, C=O), 151.99 (CH, triaz), 144.21 (CH, triaz), 137.77 (C, Ar), 135.96 (C, Ar), 132.12 (C, Ar), 129.25 (2×CH, Ar), 128.35 (CH, Ar), 128.14 (3×CH, Ar), 127.81 (2×CH, Ar), 118.75 (CH, Ar), 80.60 (*C*(CH_3_)_3_), 54.71 (*C*HCH_2_), 52.05 (CH*C*H_2_), 42.15 (NH*C*H_2_), 28.33 (C(*C*H_3_)_3_); HRMS (ESI), *m/z* calcd for C_23_H_28_N_5_O_3_ ([*M*+H]^+^), 422.2218; found: 422.2187; HPLC (Method A): 99.7 %, *t*_R_=4.82 min. *tert‐Butyl (4‐((2‐phenylacrylamido)methyl)phenyl)carbamate (**6 l**)* was obtained as white solid, yield 0.06 g (9 %); m.p. 136–138 °C; TLC (petroleum ether/EtOAc 1 : 1 *v*/*v*); *R*_f_=0.7; ^1^H NMR (CDCl_3_): *δ* 7.38 (m, 5H, Ar), 7.33 (d, *J*=8.5 Hz, 2H, Ar), 7.21 (d, *J*=8.5 Hz, 2H, Ar), 6.53 (s, 1H, N*H*), 6.21 (d, *J*=1.3 Hz, 1H, C=C*Ha*Hb), 5.97 (brs, 1H, N*H*), 5.65 (d, *J*=1.3 Hz, 1H, C=CHa*Hb*), 4.49 (d, *J*=5.6 Hz, 2H, NHC*H_2_*), 1.53 (s, 9H, C(C*H_3_*)_*3*_); ^13^C NMR (CDCl_3_): *δ* 167.07 (C, C=O), 152.73 (C, C=O), 144.61 (C, *C*=CHaHb), 137.77 (C, Ar), 136.97 (C, Ar), 132.60 (C, Ar), 128.77 (2×CH, Ar), 128.57 (CH, Ar), 128.50 (3×CH, Ar), 128.82 (2×CH, Ar), 122.64 (C=*C*H_2_), 118.78 (CH, Ar), 80.64 (*C*(CH_3_)_3_), 43.46 (NH*C*H_2_), 28.34 (C(*C*H_3_)_3_); HRMS (ESI), *m/z* calcd for C_21_H_25_N_2_O_3_ ([*M*+H]^+^), 353.1876; found: 353.1860.

***N*** **‐(4‐Chlorobenzyl)‐2‐phenylacrylamide (6 c, R^1^=4‐Cl, R^2^=H)**. Prepared from 3‐((4‐chlorobenzyl)amino)‐3‐oxo‐2‐phenylpropyl methanesulfonate (**4 c**; 0.38 g, 1.03 mmol) and purified by gradient column chromatography eluting the alkene (**6 c**) with petroleum ether/EtOAc 70 : 30 *v*/*v*. Product was obtained as a white solid, yield 0.09 g (23 %); m.p. 118–120 °C; TLC (petroleum ether/EtOAc 1 : 1 *v*/*v*); *R*_f_=0.75; ^1^H NMR ([D_6_]DMSO): *δ* 8.76 (t, *J*=5.9 Hz, 1H, N*H*), 7.38 (m, 9H, Ar), 5.79 (s, 1H, C=C*Ha*Hb), 5.69 (s, 1H, C=CHa*Hb*), 4.37 (d, *J*=6.1 Hz, 2H, NHC*H_2_*; ^13^C NMR ([D_6_]DMSO): *δ* 168.72 (C, C=O), 145.53 (C, *C*=CH_2_), 139.13 (C, Ar), 137.12 (C, Ar), 131.77 (C, C−Cl), 129.54 (2×CH, Ar), 128.80 (2×CH, Ar), 128.73 (2×CH, Ar), 128.59 (CH, Ar), 127.54 (2×CH, Ar), 118.40 (C=*C*H_2_), 42.22 (NH*C*H_2_); elemental analysis calcd (%) for C_16_H_14_ClNO (271.7457): C 70.72, H 5.19, N 5.15; found: C 70.44, H 5.16, N 5.04.

**2‐Phenyl‐*N*‐(4‐(phenylsulfonamido)benzyl)‐3‐(1*H*‐1,2,4‐triazol‐1‐yl)propanamide (12 a, R^1^=H) and 2‐phenyl‐*N*‐(4‐(phenylsulfonamido)benzyl) acrylamide (13 a, R^1^=H)**. Prepared from 3‐oxo‐2‐phenyl‐3‐((4‐(phenylsulfonamido)benzyl)amino)propyl methanesulfonate (**11 a**; 0.32 g, 0.65 mmol) and purified by gradient column chromatography eluting the alkene (**13 a**) with petroleum ether/EtOAc 40 : 60 *v*/*v*, followed by the triazole (**12 a**) with CH_2_Cl_2_/MeOH 90 : 10 *v*/*v. 2‐Phenyl‐N‐(4‐(phenylsulfonamido)benzyl)‐3‐(1H‐1,2,4‐triazol‐1‐yl)propanamide (**12 a**)* was obtained as a white solid, yield 0.06 g (19 %); m.p. 96–98 °C; TLC (petroleum ether/EtOAc 1 : 1 *v*/*v*); *R*_f_=0.0; ^1^H NMR ([D_6_]DMSO): *δ* 10.20 (brs, 1H, N*H*SO_2_), 8.58 (t, *J*=5.9 Hz, 1H, N*H*), 8.30 (s, 1H, triazole), 7.92 (s, 1H, triazole), 7.73 (d, *J*=7.1 Hz, 2H, Ar), 7.61 (t, *J*=7.4 Hz, 1H, Ar), 7.54 (t, *J*=7.5 Hz, 2H, Ar), 7.31 (m, 5H, Ar), 6.93 (d, *J*=8.6 Hz, 2H, Ar), 6.79 (d, *J*=8.6 Hz, 2H, Ar), 4.80 (dd, *J*=9.1, 13.5 Hz, 1H, CHC*Ha*Hb), 4.42 (dd, *J*=6.6, 13.5 Hz, 1H, CHCHa*Hb*), 4.18 (ddd, *J*=6.5, 15.4, 20.9 Hz, 2H, C*H*CHaHb+NHC*Ha*Hb), 3.97 (dd, *J*=5.8, 15.4 Hz, 1H, NHCHa*Hb*); ^13^C NMR ([D_6_]DMSO): *δ* 170.59 (C, C=O), 151.89 (CH, triazole), 145.63 (CH, triazole), 139.93 (C, Ar), 137.50 (C, Ar), 136.68 (C, Ar), 135.23 (C, Ar), 133.33 (CH, Ar), 129.70 (2×CH, Ar), 128.96 (2×CH, Ar), 128.21 (2×CH, Ar), 128.05 (2×CH, Ar), 127.91 (CH, Ar), 127.09 (2×CH, Ar), 120.51 (2×CH, Ar), 51.52 (*C*HCH_2_), 51.08 (CH*C*H_2_), 41.78 (NH*C*H_2_); HRMS (ESI), *m/z* calcd for C_24_H_24_N_5_O_3_S ([*M*+H]^+^), 462.1594; found: 462.1613; HPLC (Method A): 99.9 %, *t*_R=_4.68 min. *2‐Phenyl‐N‐(4‐(phenylsulfonamido)benzyl) acrylamide (**13 a**)* was obtained as white waxy solid, yield 0.15 g (51 %); TLC (petroleum ether/EtOAc 1 : 1 *v*/*v*); *R*_f_=0.46; ^1^H NMR ([D_6_]DMSO): *δ* 10.25 (brs, 1H, N*H*SO_2_), 8.63 (t, *J*=6.0 Hz, 1H, N*H*), 7.77 (d, *J*=7.1 Hz, 2H, Ar), 7.60 (t, *J*=7.1 Hz, 1H, Ar), 7.54 (t, *J*=7.45 Hz, 2H, Ar), 7.36 (m, 5H, Ar), 7.16 (d, *J*=8.6 Hz, 2H, Ar), 7.06 (d, *J*=8.6 Hz, 2H, Ar), 5.76 (s, 1H, C=C*Ha*Hb), 5.64 (s, 1H, C=CHa*Hb*), 4.27 (d, *J*=6.1 Hz, 2H, NHC*H_2_*); ^13^C NMR ([D_6_]DMSO): *δ* 168.65 (C, C=O), 145.56 (C, *C*=CH_2_), 140.00 (C, Ar), 137.13 (C, Ar), 136.71 (C, Ar), 135.84 (C, Ar), 133.33 (CH, Ar), 129.71 (2×CH, Ar), 128.77 (2×CH, Ar), 128.55 (CH, Ar), 128.42 (2×CH, Ar), 127.49 (2×CH, Ar), 127.11 (2×CH, Ar), 120.69 (2×CH, Ar), 118.18 (C=*C*H_2_), 42.21 (NH*C*H_2_); HRMS (ESI), *m/z* calcd for C_22_H_21_N_2_O_3_S ([*M*+H]^+^), 393.1267; found: 393.1255.

***N*** **‐(4‐((4‐Fluorophenyl)sulfonamido)benzyl)‐2‐phenyl‐3‐(1*H*‐1,2,4‐triazol‐1‐yl) propanamide (12 b, R^1^=4‐F) and** ***N*** **‐(4‐((4‐fluorophenyl)sulfonamido)benzyl)‐2‐phenylacrylamide (13 b, R^1^=4‐F)**. Prepared from *3‐oxo‐2‐phenyl‐3‐((4‐(4‐fluorophenylsulfonamido)benzyl)amino)propyl methanesulfonate (**11 b***; 0.31 g, 0.62 mmol) and purified by gradient column chromatography eluting the alkene (**13 b**) with petroleum ether/EtOAc 50 : 50 *v*/*v*, followed by the triazole (**12 b**) with CH_2_Cl_2_/MeOH 90 : 10 *v*/*v. N‐(4‐((3‐Fluorophenyl)sulfonamido)benzyl)‐2‐phenyl‐3‐(1H‐1,2,4‐triazol‐1‐yl) propanamide (**12 b**)* was obtained as a white solid, yield 0.09 g (30 %); m.p. 78–80 °C; TLC (petroleum ether/EtOAc 1 : 1 *v*/*v*); *R*_f_=0.0; ^1^H NMR ([D_6_]DMSO): *δ* 10.21 (brs, 1H, N*H*SO_2_), 8.59 (t, *J*=5.9 Hz, 1H, N*H*), 8.31 (s, 1H, triazole), 7.93 (s, 1H, triazole), 7.79 (dd, *J*=5.2, 8.9 Hz, 2H, Ar), 7.34 (m, 7H, Ar), 6.93 (d, *J*=8.5 Hz, 2H, Ar), 6.81 (d, *J*=8.5 Hz, 2H, Ar), 4.81 (dd, *J*=9.1, 13.5 Hz, 1H, CHC*Ha*Hb), 4.43 (dd, *J*=6.6, 13.5 Hz, 1H, CHCHa*Hb*), 4.19 (m, 2H, C*H*CHaHb+NHC*Ha*Hb), 3.99 (dd, *J*=5.4, 15.4 Hz, 1H, NHCHa*Hb*); ^13^C NMR ([D_6_]DMSO): *δ* 170.60 (C, C=O), 165.73 (C, Ar), 163.73 (C, Ar), 151.90 (CH, triazole), 143.33 (CH, triazole), 137.51 (C, Ar), 136.49 (C, Ar), 135.48 (C, Ar), 130.21 (CH, Ar), 130.13 (CH, Ar), 128.96 (2×CH, Ar), 128.22 (2×CH, Ar), 128.08 (2×CH, Ar), 127.90 (CH, Ar), 120.76 (2×CH, Ar), 117.00 (CH, Ar), 116.82 (CH, Ar), 51.53 (*C*HCH_2_), 51.09 (*C*HCH_2_), 41.78 (NH*C*H_2_); elemental analysis calcd (%) for C_24_H_22_FN_5_O_3_S**⋅**0.1 H_2_O (481.32942): C 59.89, H 4.65, N 14.55; found: C 59.52, H 4.49, N 14.26; HPLC (Method A): 99.9 %, *t*_R_=4.69 min. *N‐(4‐((4‐Fluorophenyl)sulfonamido)benzyl)‐2‐phenylacrylamide (**13 b**)* was obtained as a white solid, yield 0.12 g (41 %); m.p. 136–138 °C; TLC (petroleum ether/EtOAc 1 : 1 *v*/*v*); *R*_f_=0.5; ^1^H NMR ([D_6_]DMSO): *δ* 10.27 (brs, 1H, N*H*SO_2_), 8.64 (t, *J*=6.1 Hz, 1H, N*H*), 7.81 (dd, *J*=5.2, 9.0 Hz, 2H, Ar), 7.36 (m, 7H, Ar), 7.17 (d, *J*=8.6 Hz, 2H, Ar), 7.06 (d, *J*=8.6 Hz, 2H, Ar), 5.76 (s, 1H, C=C*Ha*Hb), 5.64 (s, 1H, C=CHa*Hb*), 4.28 (d, *J*=6.1 Hz, 2H, NHC*H_2_*); ^13^C NMR ([D_6_]DMSO): *δ* 168.65 (C, C=O), 165.73 (C, Ar), 163.73 (C, Ar), 145.56 (C, *C*=CH_2_), 137.13 (C, Ar), 136.50 (C, Ar), 136.09 (C, Ar), 130.22 (CH, Ar), 130.15 (CH, Ar), 128.76 (2×CH, Ar), 128.54 (CH, Ar), 128.47 (2×CH, Ar), 127.49 (2×CH, Ar), 120.93 (2×CH, Ar), 118.20 (C=*C*H_2_), 117.02 (CH, Ar), 116.84 (CH, Ar), 42.20 (NH*C*H_2_); HRMS (ESI), *m/z* calcd for C_22_H_20_FN_2_O_3_S ([*M*+H]^+^), 411.1173; found: 411.1173.

***N*** **‐(4‐((4‐Chlorophenyl)sulfonamido)benzyl)‐2‐phenyl‐3‐(1*H*‐1,2,4‐triazol‐1‐yl) propanamide (12 c, R^1^=4‐Cl) and** ***N*** **‐(4‐((4‐chlorophenyl)sulfonamido)benzyl)‐2‐phenylacrylamide (13 c, R^1^=4‐Cl)**. Prepared from *3‐((4‐((4‐chlorophenyl)sulfonamido)benzyl)amino)‐3‐oxo‐2‐phenylpropyl methanesulfonate (**11 c***; 0.20 g, 0.41 mmol) and purified by gradient column chromatography eluting the alkene (**13 c**) with petroleum ether/EtOAc 50 : 50 *v*/*v*, followed by the triazole (**12 c**) with CH_2_Cl_2_/MeOH 90 : 10 *v*/*v. N‐(4‐((4‐Chlorophenyl)sulfonamido)benzyl)‐2‐phenyl‐3‐(1H‐1,2,4‐triazol‐1‐yl) propanamide (**12 c**)* was obtained as an off‐white solid, yield 0.02 g (11 %); m.p. 122–124 °C; TLC (petroleum ether/EtOAc 1 : 1 *v*/*v*); *R*_f_=0.0; ^1^H NMR ([D_6_]DMSO): *δ* 10.27 (brs, 1H, N*H*SO_2_), 8.59 (t, *J*=5.9 Hz, 1H, N*H*), 8.31 (s, 1H, triazole), 7.92 (s, 1H, triazole), 7.72 (d, *J*=8.8 Hz, 2H, Ar), 7.63 (d, *J*=8.8 Hz, 2H, Ar), 7.30 (m, 5H, Ar), 6.92 (d, *J*=8.6 Hz, 2H, Ar), 6.81 (d, *J*=8.6 Hz, 2H, Ar), 4.81 (dd, *J*=9.1, 13.5 Hz, 1H, CHC*Ha*Hb), 4.43 (dd, *J*=6.6, 13.5 Hz, 1H, CHCHa*Hb*), 4.19 (m, 2H, C*H*CHaHb+NHC*Ha*Hb), 3.99 (dd, *J*=5.4, 15.4 Hz, 1H, NHCHa*Hb*); ^13^C NMR ([D_6_]DMSO): *δ* 170.60 (C, C=O), 151.89 (CH, triazole), 143.81 (CH, triazole), 138.76 (C, Ar), 138.21 (C, C−Cl), 137.51 (C, Ar), 136.33 (C, Ar), 135.61 (C, Ar), 129.89 (2×CH, Ar), 129.07 (2×CH, Ar), 128.96 (2×CH, Ar), 128.22 (2×CH, Ar), 128.12 (2×CH, Ar), 127.90 (CH, Ar), 120.85 (2×CH, Ar), 51.52 (*C*HCH_2_), 51.09 (CH*C*H_2_), 41.78 (NH*C*H_2_); HRMS (ESI), *m/z* calcd for C_24_H_23_ClN_5_O_3_S ([*M*+H]^+^), 455.1119; found: 455.1143; HPLC (Method A): 99.9 %, *t*_R_=4.75 min. *N‐(4‐((3‐Chlorophenyl)sulfonamido)benzyl)‐2‐phenylacrylamide (**13 c**)* was obtained as a white solid, yield 0.10 g (50 %); m.p. 164–166 °C; TLC (petroleum ether/EtOAc 1 : 1 *v*/*v*); *R*_f_=0.73; ^1^H NMR ([D_6_]DMSO): *δ* 10.33 (brs, 1H, N*H*SO_2_), 8.64 (t, *J*=6.1 Hz, 1H, N*H*), 7.75 (d, *J*=8.8 Hz, 2H, Ar), 7.63 (d, *J*=8.8 Hz, 2H, Ar), 7.36 (m, 5H, Ar), 7.18 (d, *J*=8.5 Hz, 2H, Ar), 7.05 (d, *J*=8.5 Hz, 2H, Ar), 5.76 (s, 1H, C=C*Ha*Hb), 5.64 (s, 1H, C=CHa*Hb*), 4.28 (d, *J*=6.1 Hz, 2H, NHC*H_2_*); ^13^C NMR ([D_6_]DMSO): *δ* 168.67 (C, C=O), 145.55 (C, *C*=CH_2_), 138.81 (C, Ar), 138.22 (C, C−Cl), 137.12 (C, Ar), 136.20 (C, Ar), 129.90 (2×CH, Ar), 129.08 (2×CH, Ar), 128.77 (2×CH, Ar), 128.55 (CH, Ar), 128.51 (2×CH, Ar), 127.49 (2×CH, Ar), 121.02 (2×CH, Ar), 118.20 (C=*C*H_2_), 42.21 (NH*C*H_2_); HRMS (ESI), *m/z* calcd for C_22_H_20_ClN_2_O_3_S ([*M*+H]^+^), 427.0884; found: 427.0872.

**2‐Phenyl‐*N*‐(4‐(4‐methoxyphenylsulfonamido)benzyl)‐3‐(1*H*‐1,2,4‐triazol‐1‐yl)propanamide (12 d, R^1^=4‐OCH_3_) and 2‐phenyl‐*N*‐(4‐(4‐methoxyphenylsulfonamido)benzyl)acrylamide (13 d, R^1^=4‐OCH_3_)**. Prepared from *3‐((4‐((4‐methoxyphenyl)sulfonamido)benzyl)amino)‐3‐oxo‐2‐phenylpropyl methanesulfonate (**11 d***; 0.40 g, 0.77 mmol) and purified by gradient column chromatography eluting the alkene (**13 d**) with petroleum ether/EtOAc 40 : 60 *v*/*v*, followed by the triazole (**12 d**) with CH_2_Cl_2_/MeOH 90 : 10, *v*/*v. N‐(4‐((4‐Methoxyphenyl)sulfonamido)benzyl)‐2‐phenyl‐3‐(1H‐1,2,4‐triazol‐1‐yl) propanamide (**12 d**)* was obtained as a white solid, yield 0.07 g (17 %); m.p. 160–162 °C; TLC (petroleum ether/EtOAc 1 : 1 *v*/*v*); *R*_f_=0.0; ^1^H NMR ([D_6_]DMSO): *δ* 10.05 (brs, 1H, N*H*SO_2_), 8.58 (t, *J*=5.9 Hz, 1H, N*H*), 8.31 (s, 1H, triazole), 7.92 (s, 1H, triazole), 7.67 (d, *J*=9.0 Hz, 2H, Ar), 7.31 (m, 5H, Ar), 7.05 (d, *J*=9.0 Hz, 2H, Ar), 6.93 (d, *J*=8.5 Hz, 2H, Ar), 6.79 (d, *J*=8.5 Hz, 2H, Ar), 4.81 (dd, *J*=9.0, 13.4 Hz, 1H, CHC*Ha*Hb), 4.43 (dd, *J*=6.6, 13.5 Hz, 1H, CHCHa*Hb*), 4.19 (m, 2H, C*H*CHaHb+NHC*Ha*Hb), 3.98 (dd, *J*=5.4, 15.4 Hz, 1H, NHCHa*Hb*), 3.80 (s, 3H, OCH_3_); ^13^C NMR ([D_6_]DMSO): *δ* 170.59 (C, C=O), 162.85 (C, *C*‐OCH_3_), 151.84 (CH, triazole), 145.04 (CH, triazole), 137.57 (CH, Ar), 136.97 (C, Ar), 135.00 (C, Ar), 131.74 (C, Ar), 129.31 (2×CH, Ar), 128.93 (2×CH, Ar), 128.22 (2×CH, Ar), 128.02 (2×CH, Ar), 128.87 (CH, Ar), 120.36 (2×CH, Ar), 114.81 (2×CH, Ar), 56.09 (O*C*H_3_), 51.58 (*C*HCH_2_), 51.18 (*C*HCH_2_), 41.87 (NH*C*H_2_); HRMS (ESI), *m/z* calcd for C_25_H_26_N_5_O_4_S ([*M*+H]^+^), 492.1706; found: 492.1695; HPLC (Method A): 99.99 %, *t*_R_=4.68 min. *N‐(4‐((4‐Methoxyphenyl)sulfonamido)benzyl)‐2‐phenylacrylamide (**13 d**)* was obtained as a white solid, yield 0.17 g (44 %); m.p. 98–100 °C; TLC (petroleum ether/EtOAc 1 : 1 *v*/*v*); *R*_f_=0.33; ^1^H NMR ([D_6_]DMSO): *δ* 10.11 (brs, 1H, N*H*SO_2_), 8.63 (t, *J*=6.1 Hz, 1H, N*H*), 7.69 (d, *J*=9.0 Hz, 2H, Ar), 7.38 (m, 5H, Ar), 8.65 (d, *J*=8.7 Hz, 2H, Ar), 7.05 (d, *J*=8.8 Hz, 4H, Ar), 5.76 (s, 1H, C=C*Ha*Hb), 5.64 (s, 1H, C=CHa*Hb*), 4.27 (d, *J*=6.1 Hz, 2H, NHC*H_2_*), 3.79 (s, 3H, OC*H_3_*); ^13^C NMR ([D_6_]DMSO): *δ* 168.65 (C, C=O), 162.84 (C, *C*‐OCH_3_), 145.56 (C, *C*=CH_2_), 136.97 (C, Ar), 135.57 (C, Ar), 131.64 (C, Ar), 129.34 (2×CH, Ar), 128.76 (2×CH, Ar), 128.54 (CH, Ar), 128.38 (2×CH, Ar), 127.49 (2×CH, Ar), 120.43 (2×CH, Ar), 118.16 (C=*C*H_2_), 114.82 (2×CH, Ar), 56.07 (O*C*H_3_), 42.21 (NH*C*H_2_); HRMS (ESI), *m/z* calcd for C_23_H_23_N_2_O_4_S ([*M*+H]^+^), 423.1379; found: 423.1368.

## Computational methods

*Molecular modelling and docking*. Docking studies were performed using the MOE program^35^ and CaCYP51 (PDB ID: 5FSA^22^) to generate pdb files of the CaCYP51 crystal structure and representative short (**5 f**) and extended (**12 c**) azole derivative complexes. All minimisations were performed with MOE until a RMSD gradient of 0.01 Kcal/mol/A with the MMFF94 forcefield and partial charges were automatically calculated. The charge of the haem iron at physiological pH was set to 3^+^ (geometry d^2^sp^3^) through the atom manager in MOE. The Alpha Triangle placement was chosen to determine the poses, refinement of the results was done using the MMFF94 forcefield, and rescoring of the refined results using the London Δ*G* scoring function was applied. The output database dock file was created with different poses for each ligand and arranged according to the final score function (*S*), which is the score of the last stage that was not set to zero.

*Molecular dynamics simulation*. Molecular dynamics simulations were run on the wild‐type/mutant [Y132H+K143R] CaCYP51 proteins in complex with fluconazole and the *R* and *S* enantiomers of **5 f** and **12 c**. PDB files were first optimised with protein preparation wizard in Maestro,^36^ version 11.8.012 by assigning bond orders, adding hydrogen, and correcting incorrect bond types. A default quick relaxation protocol was used to minimise the MD systems with the Desmond programme.^36^ In Desmond, the volume of space in which the simulation takes place, the global cell, is built up by regular 3D simulation boxes, which was utilised as part of this system for protein interactions. The orthorhombic water box allowed for a 10 Å buffer region between protein atoms and box sides. Overlapping water molecules were deleted, and the systems were neutralised with Na^+^ ions and salt concentration 0.15 M. Force‐field parameters for the complexes were assigned using the OPLS_2005 forcefield, that is, a 100 ns molecular dynamic run in the NPT ensemble (*T*=300 K) at a constant pressure of 1 bar. Energy and trajectory atomic coordinate data were recorded at each 1.2 ns.

*Binding affinity (ΔG) calculations*. Prime/MMGBAS,^37^ available in Schrödinger Prime suite,^36^ was used to calculate the binding free energy of the ligands with CaCYP51.

Δ*G* (bind)=E_complex (minimised)−(E_ligand (minimised)+E_receptor (minimised))

The mean Δ*G* (bind) was calculated from each frame from the point where the complex reached equilibrium to the final frame of the MD stimulation.

## Biological assays

*Recombinant CYP51 protein studies. Candida albicans* CYP51 (CaCYP51) and truncated human CYP51 (Δ60HsCYP51) proteins were expressed in *Escherichia coli*, isolated and purified as previously described.^18^, ^21^ The Δ60HsCYP51 protein was shown to have near identical azole binding properties to the full‐length HsCYP51.^21^ CYP51 concentrations were determined by dithionite‐reduced carbon monoxide difference spectroscopy^39^ using an extinction coefficient for the red‐shifted soret peak at ∼450 nm of 91 mM^−1^ cm^−1^.^32^ Absolute spectra for both CYP51 enzymes between 300 and 700 nm were also determined.^40^ Azole antifungal compounds were progressively titrated against 3 μM native (azole titratable) CYP51 as previously described^21^ and the type II absorbance difference spectra between 500 and 350 nm measured after each incremental addition of azole. All azole ligand binding determinations were performed in triplicate, except the binding of compound **12 c** with Δ60HsCYP51 which was performed six times. CYP51 protein samples were diluted with 0.1 M Tris**⋅**HCl (pH 8.1) and 25 % (*w*/*v*) glycerol to the required concentration.

*Susceptibility testing of C. albicans strains*. Minimum Inhibitory Concentration (MIC) determinations were performed according to recommendations outlined in the Clinical and Laboratory Standards Institute (CLSI) document M27‐S4;^28^ this includes testing in RPMI‐1640 with 0.165 M MOPS as the buffer (pH 7.0), an inoculum size of 1–5×10^4^ cells mL^−1^, and incubation at 37 °C for 48 h. The MICs were measured as the lowest concentrations of each antifungal agent that resulted in an 80 % reduction in turbidity as compared with a drug‐free, growth control well. Stock solutions of each agent were prepared in DMSO. Further dilutions were made in RPMI‐1640, and the final concentration of DMSO was 1 % (*v*/*v*). The final testing concentrations for all compounds ranged from 0.03–16 μg mL^−1^.

*CYP51 reconstitution assays*. IC_50_ values were determined for individual azole compounds^18^, ^30^ using lanosterol as substrate. Azoles were introduced using 2.5 μL of stock solutions in DMSO. CaCYP51 assays contained 1 μM CaCYP51 and 2 μM *Homo sapiens* cytochrome P450 reductase (HsCPR – UniProtKB accession number P16435) and were incubated for 20 min at 37 °C. HsCYP51 assays contained 0.25 μM Δ60HsCYP51 and 1 μM HsCPR and were incubated for 10 min at 37 °C. Each IC_50_ experiment was performed in duplicate. IC_50_ is defined here as the inhibitory concentration of compound that causes a 50 % reduction in observed enzyme activity under the stated assay conditions.

*Dissociation constant* (*K*_d_). The *K*_d_ for each CYP51‐azole complex was determined by non‐linear regression (Levenberg‐Marquardt algorithm) using a rearrangement of the Morrison equation for tight ligand binding.^34^ Where ligand binding was weaker, the Michaelis‐Menten equation was used to fit the data (Figure S5). *K*_d_ values were determined for each of the three replicate titrations per azole compound and then mean *K*_d_ values and standard deviations calculated.

*Sterol profile analysis of C. albicans strains*. Sterol extractions were performed on cells grown in 10 mL of MOPS buffered (0.165 M) RPMI‐1640, pH 7.0 containing either DMSO alone (1 % *v*/*v*), or DMSO containing antifungal (final concentration 0.06 μg mL^−1^ fluconazole, 0.06 μg mL^−1^ **5 d**, 0.015 μg mL^−1^ **5 f** or 0.5 μg mL^−1^ **12 c**). The culture medium was then innoculted with *C. albicans*, (either CA14 or SC5314), to a final density of 5×10^4^ cells mL^−1^ and the cultures grown at 37 °C, 180 rpm, for 18 h. Cells were pelleted and washed with ddH_2_O and non‐saponifiable lipids were extracted using methanolic KOH as reported previously.^41^ Samples were dried in a vacuum centrifuge and were derivatised by the addition of 100 μL 90 % BSTFA / 10 % TMCS (Sigma), 200 μL anhydrous pyridine (Sigma) and heating for 2 h at 80 °C. TMS‐derivatised sterols were analysed and identified by using GC/MS (Thermo 1300 GC coupled to a Thermo ISQ mass spectrometer, Thermo Scientific) with reference to retention times and fragmentation spectra for known standards. GC/MS data files were analysed using Xcalibur software (Thermo Scientific) to determine sterol profiles for all isolates and to integrate peak areas.

## Conflict of interest

The authors declare no conflict of interest.

## Supporting information

As a service to our authors and readers, this journal provides supporting information supplied by the authors. Such materials are peer reviewed and may be re‐organized for online delivery, but are not copy‐edited or typeset. Technical support issues arising from supporting information (other than missing files) should be addressed to the authors.

Supplementary
