# Supplementary material for: Small‐Molecule Inhibitors Targeting Sterol 14α‐Demethylase (CYP51): Synthesis, Molecular Modelling and Evaluation Against Candida albicans
Source: ChemMedChem. 2020 Jun 22;15(14):1294–309. doi: 10.1002/cmdc.202000250 (PMC7496091; doi:10.1002/cmdc.202000250)
Supplement: Supplementary file 1 — Supplementary [file CMDC-15-1294-s001.pdf]

ChemMedChem

Supporting Information

Small-Molecule Inhibitors Targeting Sterol 14α-Demethylase (CYP51): Synthesis, Molecular Modelling and Evaluation Against *Candida albicans*

Faizah A. Binjubair, Josie E. Parker, Andrew G. Warrilow, Kalika Puri, Peter J. Braidley, Esra Tatar, Steven L. Kelly, Diane E. Kelly, and Claire Simons*

Supporting Information

S2	**Figure S1**. *Spectral characterization of CaCYP51 and Δ60HsCYP51*
S3-S4	**Figure S2**. *CaCYP51 type II azole binding difference spectra*
S5	**Figure S3**. *CaCYP51 azole saturation curves*
S6	**Figure S4**. *Δ60HsCYP51 azole inhibition profiles*
S7	**Figure S5.** A schematic of detailed ligand atom interactions of representative (*S*)-enantiomers of short derivative **5f** and extended derivative **12c** with the protein residues of wild-type CaCYP51 active site. Interactions that occur more than 30.0% of the simulation time in the selected trajectory (0 through 100 ns) are shown.
S8-S19	Procedures and characterisation of synthesised compounds
S20-S27	HPLC traces of final **5** and **12** series compounds
S27	References

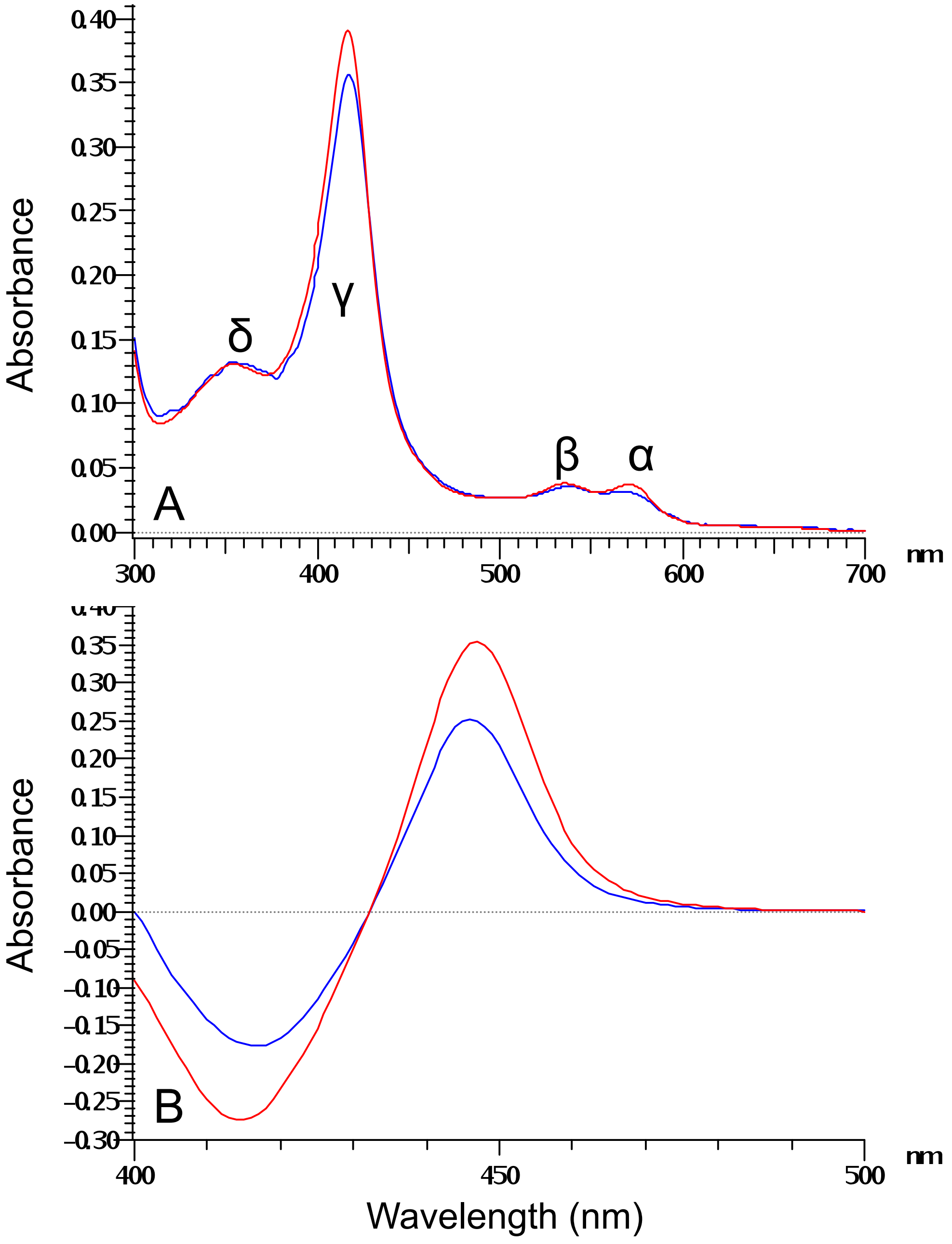

Figure S1. *Spectral characterization of CaCYP51 and Δ60HsCYP51*. Absolute spectra (A) for ten-fold dilutions of purified CaCYP51 (blue line) and Δ60HsCYP51 (red line) are shown along with the dithionite-reduced carbon monoxide difference spectra (B).

A

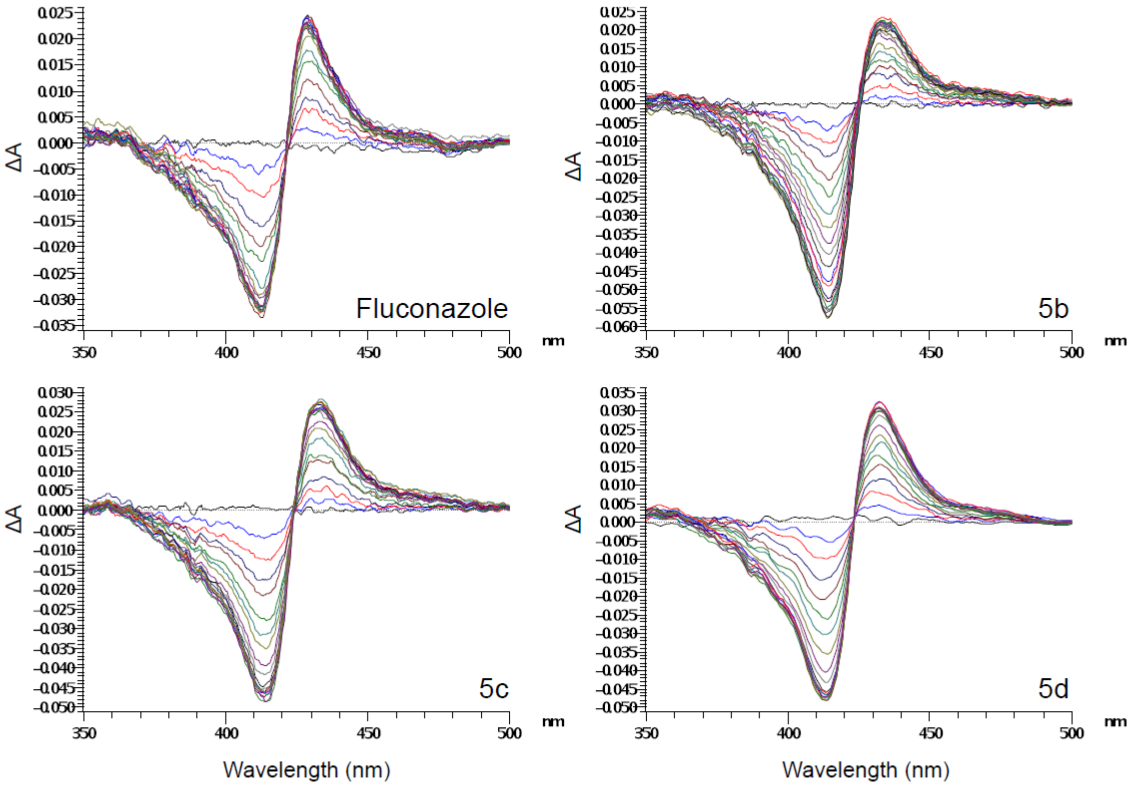

ΔA
Fluconazole
5b
5c
5d
nm
350
400
450
500
Wavelength (nm)

B

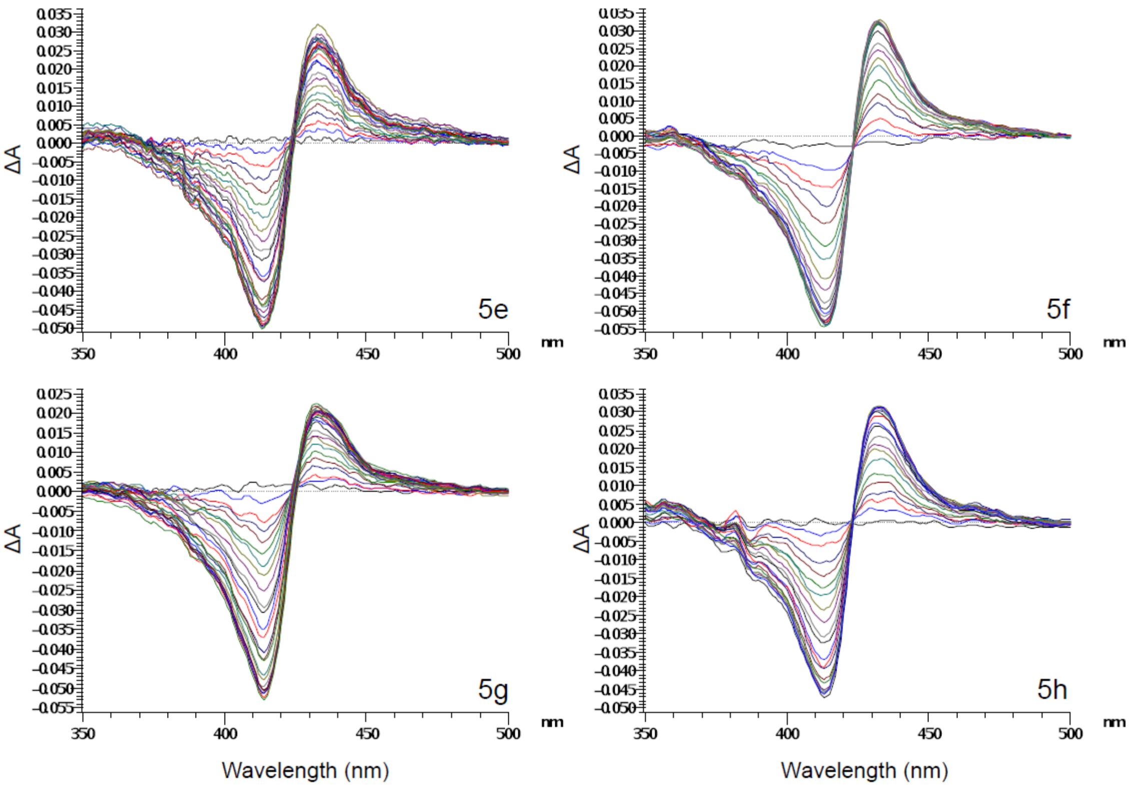

ΔA
5e
5f
5g
5h
nm
350
400
450
500
Wavelength (nm)

C

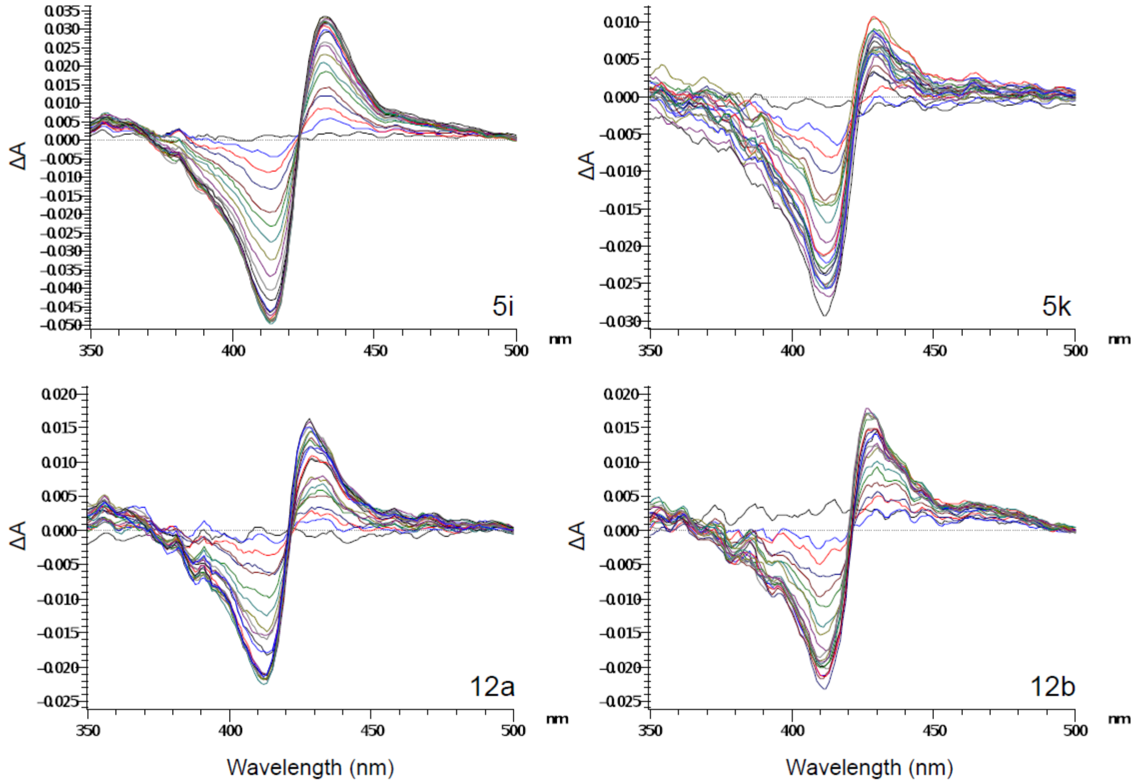

D

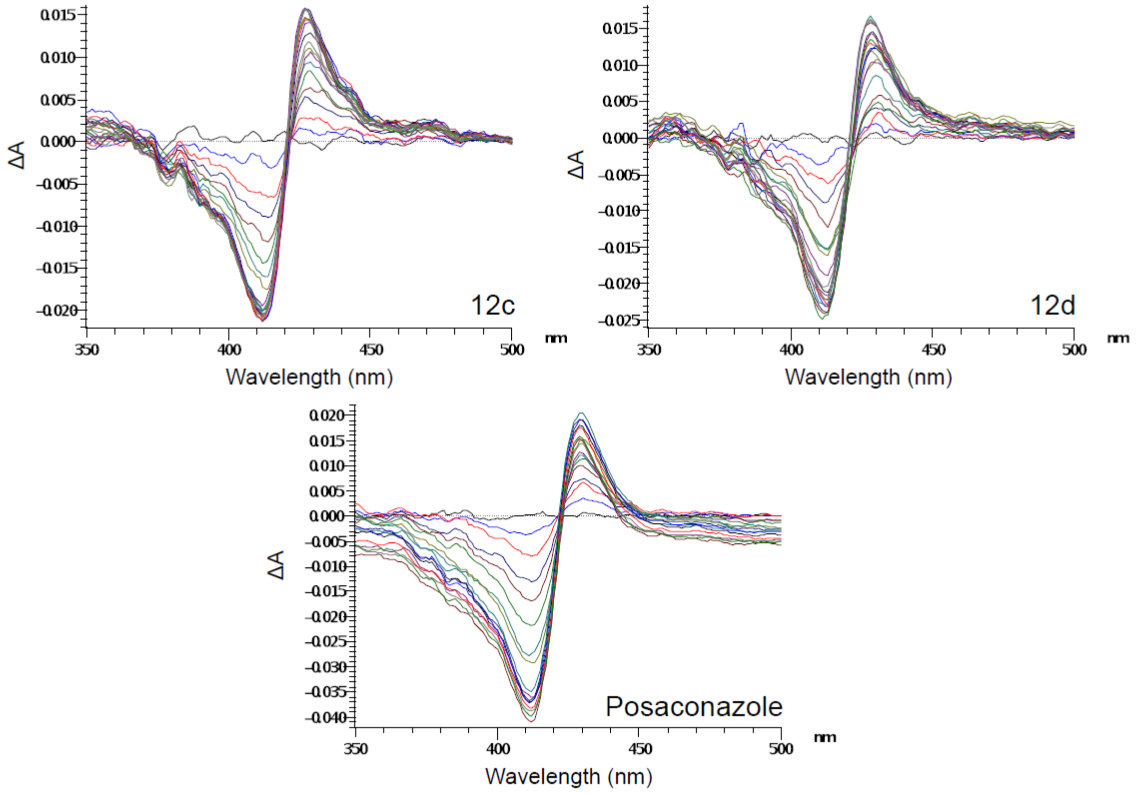

Figure S2. *CaCYP51 type II azole binding difference spectra*. Type II difference spectra are shown for the binding of *Panel A* - fluconazole, **5b**, **5c** and **5d**; *Panel B* - **5e**, **5f**, **5g** and **5h**; *Panel C* - **5i**, **5k**, **12a** and **12b**; *Panel D* - **12c**, **12d** and posaconazole, with 3 μM native CaCYP51. Each azole titration was performed in triplicate although only one replicate is shown.

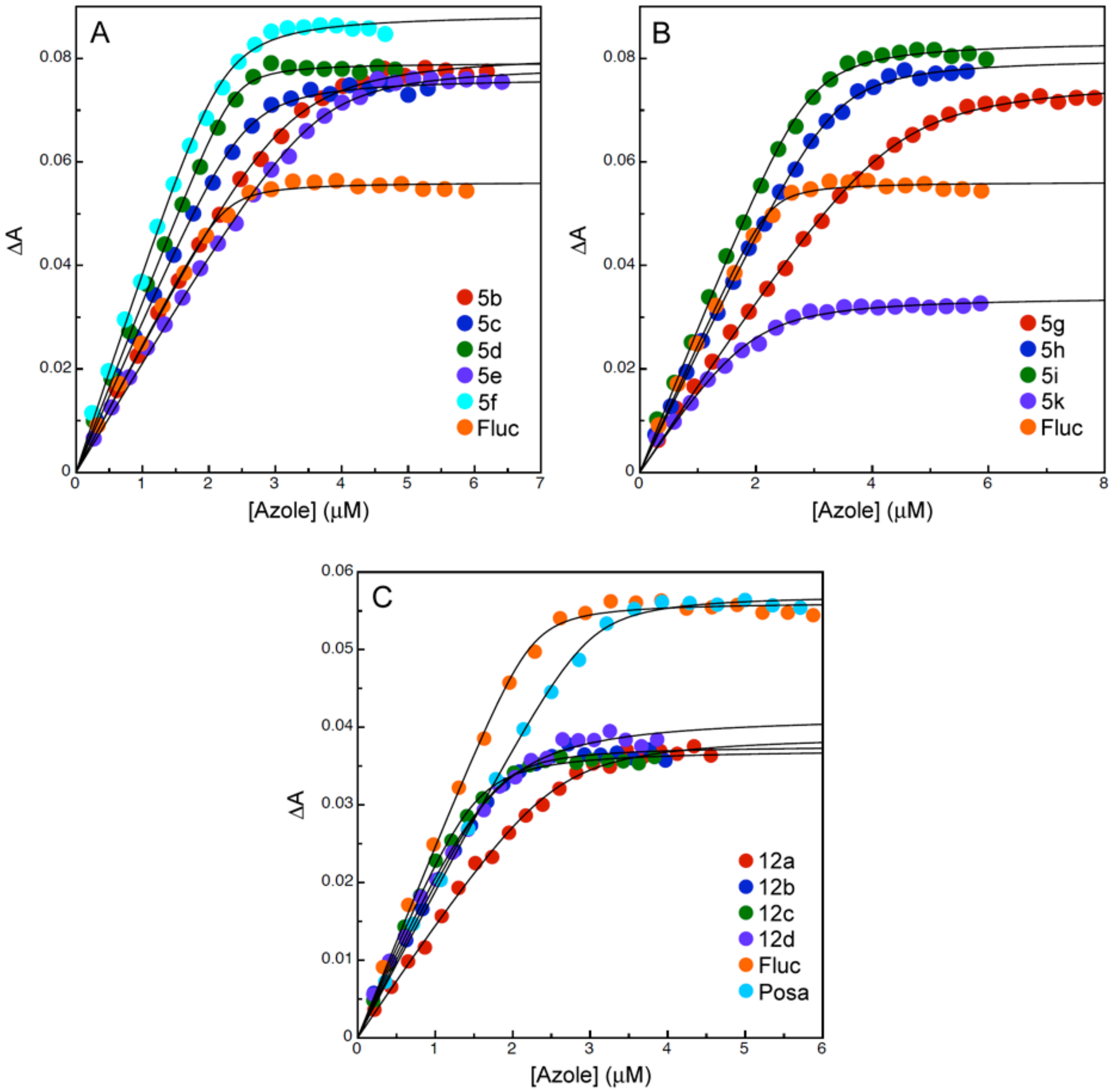

Figure S3. *CaCYP51 azole saturation curves*. Azole ligand binding saturation curves derived from the type II difference spectra for *Panel A* - fluconazole (●), **5b** (●), **5c** (●), **5d** (●) **5e** (●) and **5f** (●); *Panel B* - fluconazole (●), **5g** (●), **5h** (●), **5i** (●) and **5k** (●); *Panel C* - fluconazole (●), **12a** (●), **12b** (●), **12c** (●), **12d** (●) and posaconazole (●), with 3 μM native CaCYP51. Each azole titration was performed in triplicate although only one replicate is shown.

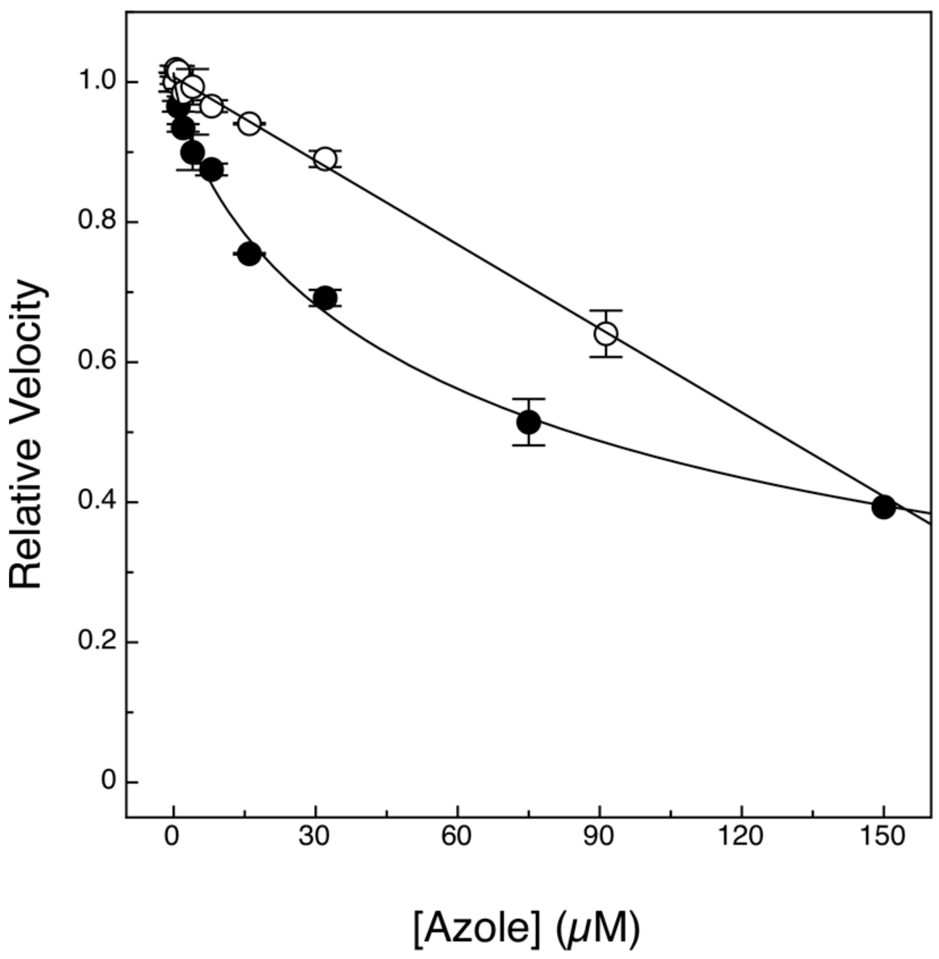

Figure S4. *Δ60HsCYP51 azole inhibition profiles*. Inhibition profiles for voriconazole (●) and posaconazole (○) were determined using CYP51 reconstitution assays containing 0.25 μM Δ60HsCYP51, 1 μM HsCPR and lanosterol as substrate. IC_{50} determinations were performed in duplicate with mean values shown along with standard deviations. A relative velocity of 1.00 corresponds to an actual velocity of 8.16 ±2.84 min^{-1}.

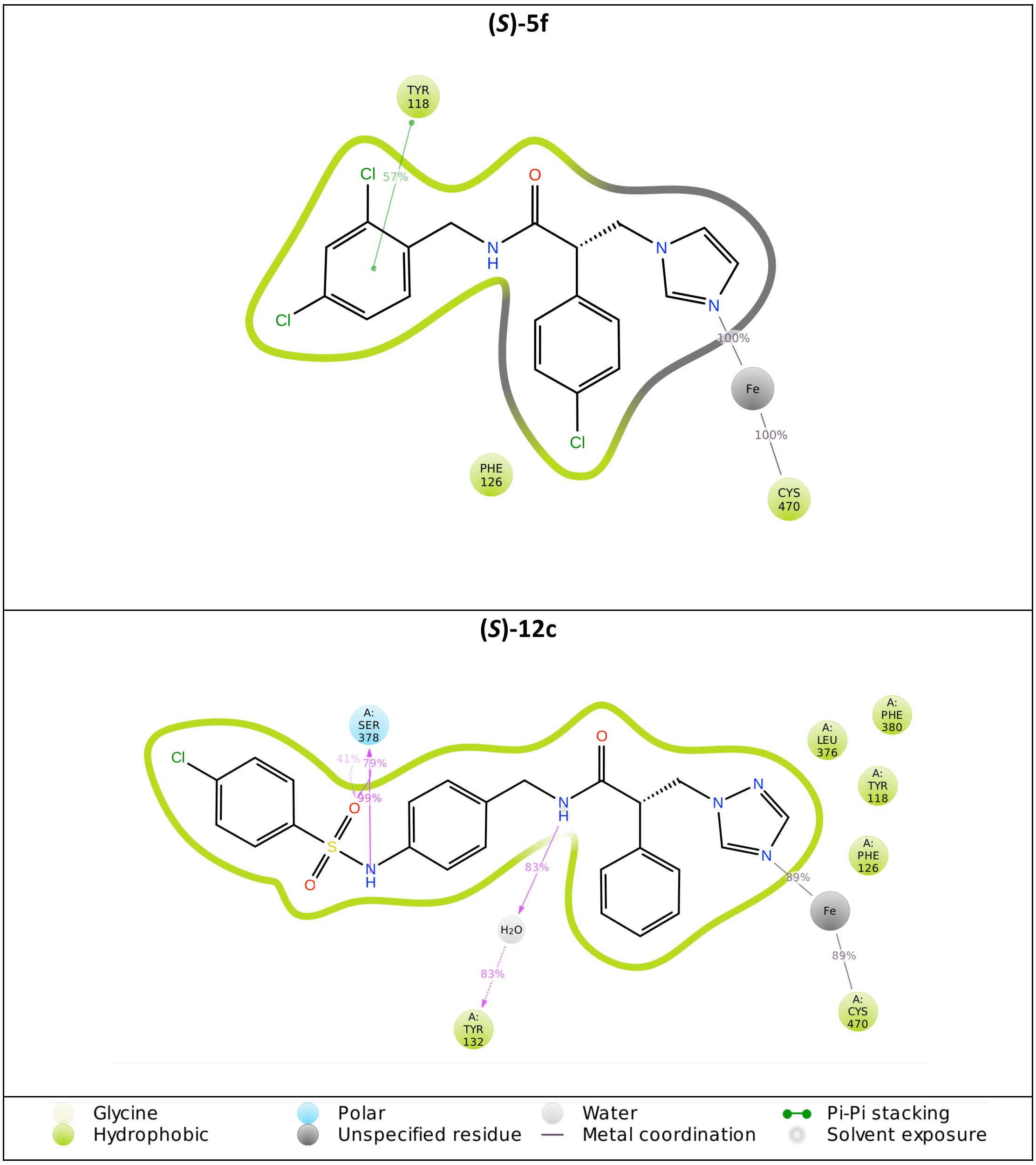

Figure S5. A schematic of detailed ligand atom interactions of representative (*S*)-enantiomers of short derivative **5f** and extended derivative **12c** with the protein residues of wild-type CaCYP51 active site. Interactions that occur more than 30.0% of the simulation time in the selected trajectory (0 through 100 ns) are shown.

.

Procedures and characterisation of synthesised compounds:

General procedure for the preparation of amides (3).

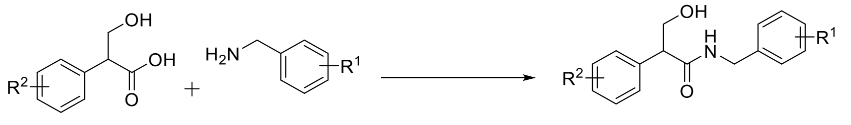

Where R²: a= 4-H
b= 4-Cl

Where R¹: a= 4-H, **b**= 4-F, **c**= 4-Cl, **d**= 2,4-di-Cl, **e**= 4-CH_3, **f**= 4-CF_3, **g**= 4-OCH_3, **h**= 4-di-OCH_3, **i**= 4-NHBoc

To a suspension of acid (**1**) (5 mmol) in dry CPME (15 mL) and activated 4Å molecular sieves (~ 1 mg/mmol) was added benzylamine (**2**) (5.5 mmol) followed by $B(OMe)_3$ (1.14 mL, 10 mmol). The resulting mixture was heated at 100 °C overnight. Upon completion, the reaction mixture was cooled to room temperature and diluted with acetone (10 mL) and H_2O (1 mL). Amberlite IRA743 (0.5 g), Amberlyst 15 (0.5 g) and Amberlyst A26 (OH) (0.5 g) resins were added and the resulting suspension was stirred for 2 h. After disappearance of any remaining starting materials (monitored by TLC), anhydrous $MgSO_4$ was added to the mixture. The reaction mixture was filtered, and the residue washed with acetone (2 x 10 mL), and the combined filtrates concentrated under reduced pressure to give the product, which was washed with Et_2O/petroleum ether to remove any remaining CPME.

***N*-Benzyl-3-hydroxy-2-phenylpropanamide (3a, R^1 = R^2 = H).** Prepared from tropic acid (**1a**, R^2 = H) and benzylamine (**2a**, R^1 = H). Product obtained as an off-white solid, yield 1.25 g (98 %). M.p. 108-110 °C (117- 119 °C lit [1]). TLC (petroleum ether-EtOAc 1:1 v/v), R*f* = 0.28. ^{1}H NMR (DMSO-d_6): *δ* 8.53 (t, *J* = 5.9 Hz, 1H, NH), 7.26 (m, 10H, Ar), 4.86 (t, *J* = 5.2 Hz, 1H, O*H*), 4.31 (dd, *J* = 6.0, 15.3 Hz, 1H, NHC*Ha*Hb), 4.24 (dd, *J* = 5.8, 15.3 Hz, 1H, NHCHa*Hb*), 4.00 (ddd, *J* = 5.6, 9.2, 15.4 Hz, CHC*Ha*Hb), 3.68 (dd, *J* = 5.5, 9.0 Hz, C*H*CHaHb), 3.55 (pentet, *J* = 5.1 Hz, CHCHa*Hb*). ^{13}C-NMR (DMSO-d_6): *δ* 172.18 (C, C=O), 139.92 (C, Ar), 138.90 (C, Ar), 128.65 (2 x CH, Ar), 128.63 (2 x CH, Ar), 128.41 (2 x CH, Ar), 127.50 (2 x CH, Ar), 127.20 (CH, Ar), 127.11 (CH, Ar), 63.83 (*C*H_2OH), 54.99 (*C*HCH_2OH), 42.46 (NH*C*H_2).

N*-(4-Fluorobenzyl)-3-hydroxy-2-phenylpropanamide (3b, R^1 = 4-F, R^2 = H)*. Prepared from tropic acid (**1a**, R^2 = H) and 4-fluorobenzylamine (**2b**, R^1 = 4-F). Product obtained as a cream solid, yield 1.22 g (89 %). M.p. 114-116 °C. TLC (petroleum ether-EtOAc 1:1 v/v), R*f* = 0.21. ^{1}H NMR (DMSO-d_6): *δ* 8.54 (t, *J* = 5.6 Hz, 1H, N*H*), 7.31 (m, 4H, Ar), 7.23 (m, 3H, Ar), 7.09 (t, *J* = 8.8 Hz, 2H, Ar), 4.87 (brs, 1H, OH), 4.31 (dd, *J* = 6.1, 15.3 Hz, 1H, NHC*Ha*Hb), 4.22 (dd, *J* = 5.7, 15.3 Hz, 1H, NHCHa*Hb*), 4.00 (t, *J* = 9.0 Hz, 1H, CHC*Ha*Hb), 3.67 (dd, *J* = 5.6, 8.8 Hz, 1H, C*H*CHaHb), 3.56 (dd, *J* = 5.6, 9.0 Hz, 1H, CHCHa*Hb*). ^{13}C-NMR (DMSO-d_6): *δ* 172.24 (C, C=O), 162.52 and 160.60 (C, C-F), 138.82 (C, Ar), 136.14 (C, Ar), 129.47 (CH, Ar), 129.41 (CH, Ar), 128.67 (2 x CH, Ar), 128.40 (2 x CH, Ar), 127.23 (CH, Ar), 115.42 (CH, Ar), 115.25 (CH, Ar), 63.84 (CH*C*H_2OH), 55.01 (*C*HCH_2OH), 41.79 (NH*C*H_2). LRMS (ES+TOF, *m/z*)*:* 274.13 $[C_{16}H_{16}FNO_2 + H]^+$, 179.02 $[C_{10}H_{13}NO_2]^+$. HRMS (ES+TOF) *m/z.* calcd for $C_{16}H_{17}FNO_2$ ($[M + H]^+$), 274.1243; found, 274.1250.

***N*-(4-Chlorobenzyl)-3-hydroxy-2-phenylpropanamide (3c, R^1 = 4-Cl, R^2 = H)**. Prepared from tropic acid (**1a**, R^2 = H) and 4-chlorobenzylamine (**2c**, R^1 = 4-Cl). Product obtained as a cream solid, yield 1.19 g (85 %). M.p. 135-136 °C. TLC (petroleum ether-EtOAc 1:1 v/v), R*f* = 0.42. ^{1}H NMR (DMSO-d_6): *δ* 8.59 (t, *J* = 6.0 Hz, 1H, N*H*), 7.40 (s, 2H, Ar), 7.30 (m, 5H, Ar), 7.22 (m, 2H, Ar), 4.30 (dd, *J* = 6.1, 15.5 Hz, 1H, NHC*Ha*Hb), 4.21 (dd, *J* = 5.8, 15.5 Hz, 1H, NHCHa*Hb*), 3.99 (t, *J* = 9.6 Hz, 1H, O*H*), 3.86 (m, 2H, CHC*HaHb*), 3.67 (dd, *J* = 5.5, 9.1 Hz, 1H, C*H*CHaHb). ^{13}C-NMR (DMSO-d_6): *δ* 172.29 (C, C=O), 139.07 (C, Ar), 138.87 (C, C-Cl), 138.77 (C, C-Cl), 131.65 (C, Ar), 130.26 (CH, Ar), 129.36 (2 x CH, Ar), 128.65 (2 x CH, Ar), 128.56 (CH, Ar), 128.39 (CH, Ar), 127.24 (CH, Ar), 127.08

(CH, Ar), 64.32 (*C*H_2OH), 54.99 (*C*HCH_2OH), 43.63 (NH*C*H_2). Anal. Calcd for $C_{16}H_{16}ClNO_2$•$0.2H_2O$ (293.3630): C, 65.51%; H, 5.63%; N, 4.77 %. Found: C, 65.36%; H, 5.47%; N, 4.84 %.

***N*-(4-Chlorobenzyl)-2-(4-chlorophenyl)-3-hydroxypropanamide (3d, R^1 = 4-Cl, R^2 = Cl).** Prepared from 2-(4-chlorophenyl)-3-hydroxy propanoic acid [2] (**1b**, R^2 = Cl) and 4-chlorobenzylamine (**2c**, R^1 = 4-Cl). Product obtained as a cream solid, yield 1.62 g (100 %). M.p. 126-128 °C. TLC (petroleum ether-EtOAc 1:1 v/v), R*f* = 0.3. ^{1}H NMR (DMSO-d_6): *δ* 8.64 (t, *J* = 5.9 Hz, 1H, N*H*), 7.40 (m, 6H, Ar), 7.26 (d, *J* = 8.5 Hz, 2H, Ar), 4.95 (brs, 1H, O*H*), 4.35 (dd, *J* = 6.1, 15.5 Hz, 1H, NHC*Ha*Hb), 4.26 (dd, *J* = 5.8, 15.5 Hz, 1H, NHCHa*Hb*), 4.00 (t, *J* = 8.9 Hz, 1H, CHC*Ha*Hb), 3.73 (dd, *J* = 5.9, 8.6 Hz, 1H, C*H*CHaHb), 3.59 (dd, *J* = 8.3, 14.5 Hz, 1H, CHCHa*Hb*). ^{13}C-NMR (DMSO-d_6): *δ* 171.92 (C, C=O), 158.93 (C, Ar), 137.76 (C, Ar), 131.95 (C, C-Cl), 131.72 (C, C-Cl), 130.28 (2 x CH, Ar), 129.39 (2 x CH, Ar), 128.64 (2 x CH, Ar), 128.61 (2 x CH, Ar), 63.68 (CH*C*H_2OH), 54.21 (*C*HCH_2OH), 41.87 (NH*C*H_2). LRMS (ES+TOF, *m/z*): 326.05 [$C_{16}H_{15}{}^{37}Cl_2NO_2$ + H]$^+$, 324.06 [$C_{16}H_{15}{}^{35}Cl_2NO_2$ + H]$^+$, 157.03 [$C_8H_8{}^{35}ClO$ + H]$^+$. HRMS (ES+TOF), *m/z*. calcd for $C_{16}H_{16}Cl_2NO_2$ ([M + H]$^+$), 324.0558; found, 324.0555.

***N*-(2,4-Dichlorobenzyl)-3-hydroxy-2-phenylpropanamide (3e, R^1 = 2,4-diCl, R^2 = H).** Prepared from tropic acid (**1a**, R^2 = H) and 2,4-chlorobenzylamine (**2d**, R^1 = 2,4-diCl). Product obtained as a brown solid, yield 1.62 g (100 %). M.p. 78-80 °C. TLC (petroleum ether-EtOAc 1:1 v/v), R*f* = 0.47. ^{1}H NMR (DMSO-d_6): *δ* 8.61 (t, *J* = 5.8 Hz, 1H, N*H*), 7.57 (d, *J* = 2.0 Hz, 1H, Ar), 7.32 (m, 5H, Ar), 7.25 (m, 2H, Ar), 4.90 (t, *J* = 7.1 Hz, 1H, OH), 4.33 (dd, *J* = 5.9, 16.1 Hz, 1H, NHC*Ha*Hb), 4.27 (dd, *J* = 5.8, 16.1 Hz, 1H, NHCHa*Hb*), 3.99 (ddd, *J* = 5.6, 9.7, 15.1 Hz, 1H, CHC*Ha*Hb), 3.72 (dd, *J* = 5.5, 9.1 Hz, 1H, C*H*CHaHb), 3.56 (d dd, *J* = 1.2, 5.1, 10.0 Hz, 1H, CHCHa*Hb*). ^{13}C-NMR (DMSO-d_6): *δ* 172.54 (C, C=O), 158.58 (C, Ar), 136.07 (C, Ar), 133.32 (C, C-Cl), 132.57 (C, C-Cl), 130.38 (CH, Ar), 128.94 (CH, Ar), 128.70 (2 x CH, Ar), 128.42 (2 x CH, Ar), 127.58 (CH, Ar), 127.31 (CH, Ar), 63.79 (CH*C*H_2OH), 54.95 (*C*HCH_2OH), 39.98 (NH*C*H_2). LRMS (ES+TOF, *m/z*): 326.05 [$C_{16}H_{15}{}^{37}Cl_2NO_2$ + H]$^+$, 324.06 [$C_{16}H_{15}{}^{35}Cl_2NO_2$ + H]$^+$, 159.03 [$C_7H_5{}^{37}Cl_2$ + H]$^+$, 157.03 [$C_7H_5{}^{35}Cl_2$ + H]$^+$. HRMS (ES+TOF), *m/z*. calcd for $C_{16}H_{16}Cl_2NO_2$ ([M + H]$^+$), 324.0558; found, 324.0562.

***N*-(2,4-Dichlorobenzyl)-2-(4-chlorophenyl)-3-hydroxypropanamide (3f, R^1 = 2,4-diCl, R^2 = Cl).** Prepared from 2-(4-chlorophenyl)-3-hydroxy propanoic acid [2] (**1b**, R^2 = Cl) and 2,4-dichlorobenzylamine (**2d**, R^1 = 2,4-diCl). Product obtained as a white solid, yield 0.71 g (40 %). M.p. 106-108 °C. TLC (petroleum ether-EtOAc 1:1 v/v), R*f* = 0.3. ^{1}H NMR (DMSO-d_6): *δ* 8.63 (t, *J* = 5.8 Hz, 1H, N*H*), 7.58 (d, *J* = 2.1 Hz, 1H, Ar), 7.36 (m, 5H, Ar), 7.25 (d, *J* = 8.4 Hz, 1H, Ar), 4.94 (t, *J* = 5.1 Hz, 1H, O*H*), 4.30 (m, 2H, NHC*HaHb*), 3.94 (ddd, *J* = 5.5, 8.9, 15.6 Hz, 1H, CHC*Ha*Hb), 3.73 (dd, *J* = 5.8, 8.8 Hz, 1H, C*H*CHaHb), 3.55 (pentet, *J* = 5.2 Hz, 1H, CHCHa*Hb*).^{13}C-NMR (DMSO-d_6): *δ* 172.13 (C, C=O), 137.57 (C, Ar), 135.95 (C, Ar), 133.37 (C, C-Cl), 132.63 (C, C-Cl), 131.99 (C, C-Cl), 130.45 (CH, Ar), 130.30 (2 x CH, Ar), 128.99 (CH, Ar), 128.66 (2 x CH, Ar), 127.65 (CH, Ar), 63.67 (CH*C*H_2OH), 54.12 (*C*HCH_2OH), 40.12 (NH*C*H_2). Anal. Calcd for $C_{16}H_{14}Cl_3NO_2$ (358.6469): C, 53.58%; H, 3.93%; N, 3.90%. Found: C, 53.72%; H, 4.06%; N, 3.85%.

3-Hydroxy-*N*-(4-methylbenzyl)-2-phenylpropanamide (3g, R^1 = 4-CH_3, R^2 = H). Prepared from tropic acid (**1a**, R^2 = H) and 4-methylbenzylamine (**2e**, R^1 = 4-CH_3). Product obtained as a cream solid, yield 1.35 g (100 %). M.p. 96-98 °C. TLC (petroleum ether-EtOAc 1:1 v/v), R*f* = 0.29. ^{1}H NMR (DMSO-d_6): *δ* 8.47 (t, *J* = 5.9 Hz, 1H, N*H*), 7.28 (m, 5H, Ar), 7.06 (m, 3H, Ar), 6.53 (s, 1H, Ar), 4.84 (t, *J* = 5.2 Hz, 1H, OH), 4.25 (dd, *J* = 6.0, 15.1 Hz, 1H, NHC*Ha*Hb), 4.19 (dd, *J* = 5.9, 15.1 Hz, 1H, NHCHa*Hb*), 3.99 (ddd, *J* = 5.6, 9.1, 15.5 Hz, 1H, CHC*Ha*Hb), 3.66 (dd, *J* = 5.5, 9.0 Hz, 1H, C*H*CHaHb), 3.54 (pentet, *J* = 5.1 Hz, 1H, CHCHa*Hb*), 2.25 (s, 3H, C*H_3*). ^{13}C-NMR (DMSO-d_6): *δ* 172.09 (C, C=O), 138.95 (C, Ar), 136.87 (C, *C*-CH_3), 136.13 (C, Ar), 129.18 (2 x CH, Ar), 128.63 (2 x CH, Ar), 128.41 (2 x CH, Ar), 127.51 (2 X CH, Ar), 127.18 (CH, Ar), 63.81 (CH*C*H_2OH), 54.97 (*C*HCH_2OH), 42.21 (NH*C*H_2), 21.09 (*C*H_3). HRMS (ESI), *m/z*. calcd for $C_{17}H_{20}NO_2$ ([M + H]$^+$), 270.1499; found, 270.1489.

3-Hydroxy-*N*-(4-trifluoromethylbenzyl)-2-phenylpropanamide (3h, R^1 = 4-CF_3, R^2 = H). Prepared from tropic acid (**1a**, R^2 = H) and 4-

trifluoromethylbenzylamine (**2f**, R^1 = 4-CF_3). Further purification by extraction with EtOAc (50 mL) washing with 1M aqueous HCl (2 x 25 mL), water (25 mL) and dried over ($MgSO_4$) before concentration under vacuum to give pure amine as a cream solid, yield 0.54 g (34 %). M.p. 104-106 °C. TLC (petroleum ether-EtOAc 1:1 v/v), R*f* = 0.20. ^{1}H NMR (DMSO-d_6): *δ* 8.66 (t, *J* = 6.0 Hz, 1H, N*H*),), 7.62 (d, *J* = 8.1 Hz, 2H, Ar), 7.40 (d, *J* = 8.0 Hz, 2H, Ar), 7.28 (m, 5H, Ar), 4.81 (brs, 1H, O*H*), 4.40 (dd, *J* = 6.1, 15.9 Hz, 1H, NHC*Ha*Hb), 4.31 (dd, *J* = 5.8, 15.8 Hz, 1H, NHCHa*Hb*), 4.00 (t, *J* = 9.6 Hz, 1H, CHC*Ha*Hb), 3.69 (dd, *J* = 5.5, 9.1 Hz, 1H, C*H*CHaHb), 3.55 (dd, *J* = 5.5, 10.1 Hz, 1H, CHCHa*Hb*). ^{13}C-NMR (DMSO-d_6): *δ* 172.43 (C, C=O), 144.97 (C, Ar), 138.71 (C, Ar), 128.69 (2 x CH, Ar), 128.40 (3 x CH, Ar), 128.12 (3 x CH, Ar), 127.70 (C, Ar), 127.27 (CH, Ar), 125.50 & 125.47 (C, C-F_3), 63.79 (CH*C*H_2OH), 55.02 (*C*HCH_2OH), 42.13 (NH*C*H_2). ^{19}F-NMR (DMSO-d_6): *δ* -60.80. Anal. Calcd for $C_{17}H_{16}F_3NO_2$•$0.2H_2O$ (326.91714): C, 62.46%; H, 5.12%; N, 4.28%. Found: C, 62.31%; H, 4.88%; N, 3.93%.

3-Hydroxy-*N*-(4-methoxybenzyl)-2-phenylpropanamide (3i, R^1 = 4-OCH$_3$, R^2 = H). Prepared from tropic acid (**1a**, R^2 = H) and 4-methoxybenzylamine (**2g**, R^1 = 4-OCH_3). Product obtained as a white solid, yield 1.40 g (98 %). M.p. 126-128 °C. TLC (petroleum ether-EtOAc 1:1 v/v), R*f* = 0.36. ^{1}H NMR (DMSO-d_6): *δ* 8.45 (t, *J* = 5.8 Hz, 1H, N*H*), 7.32 (m, 4H, Ar), 7.23 (m, 1H, Ar), 7.12 (d, *J* = 8.7 Hz, 2H, Ar), 6.83 (d, *J* = 8.7 Hz, 2H, Ar), 4.67 (dd, *J* = 8.8, 9.7 Hz, 1H, OH), 4.25 (dd, *J* = 6.0, 15.0 Hz, 1H, NHC*Ha*Hb), 4.17 (dd, *J* = 5.8, 15.0 Hz, 1H, NHCHa*Hb*), 4.00 (dd, *J* = 9.2, 9.9 Hz, 1H, CHC*Ha*Hb), 3.72 (s, 3H, C*H$_3$*), 3.57 (dd, *J* = 5.5, 8.9 Hz, 1H, C*H*CHaHb), 3.55 (dd, *J* = 5.5, 10.1 Hz, 1H, CHCHa*Hb*). ^{13}C-NMR (DMSO-d_6): *δ* 172.06 (C, C=O), 158.61 (C, C-OCH_3), 138.97 (C, Ar), 131.83 (C, Ar), 128.86 (2 x CH, Ar), 128.63 (2 x CH, Ar), 128.42 (2 x CH, Ar), 127.17 (CH, Ar), 114.07 (2 x CH, Ar), 63.87 (CH*C*H_2OH), 55.51 (O*C*H_3), 54.97 (*C*HCH_2OH), 41.95 (NH*C*H_2). Anal. Calcd for $C_{17}H_{19}NO_3$ (285.3420): C, 71.56%; H, 6.71%; N, 4.91%. Found: C, 71.57%; H, 6.68%; N, 5.08%.

***N*-(3,4-Dimethoxybenzyl)-3-hydroxy-2-phenylpropanamide (3j, R^1 = 3,4-diOCH$_3$, R^2 = H**). Prepared from tropic acid (**1a**, R^2 = H) and 3,4-dimethoxybenzylamine (**2h**, R^1 = 3,4-OCH_3). Product obtained as a yellow solid, yield 1.05 g (83 %). M.p. 116-118 °C. TLC (petroleum ether-EtOAc 1:1 v/v), R*f* = 0.16. ^{1}H NMR (DMSO-d_6): *δ* 8.49 (t, *J* = 5.9 Hz, 1H, N*H*), 7.35 (d, *J* = 7.5 Hz, 2H, Ar), 7.30 (t, *J* = 7.5 Hz, 2H, Ar), 7.23 (t, *J* = 7.1 Hz, 1H, Ar), 6.83 (d, *J* = 8.1 Hz, 1H, Ar), 6.72 (d, *J* = 3.2 Hz, 1H, Ar), 6.54 (s, 1H, Ar), 4.86 (t, *J* = 5.1 Hz, 1H, OH), 4.23 (m, 2H, NHC*HaHb*), 4.02 (ddd, *J* = 5.6, 9.6, 15.0 Hz, 1H, CHC*Ha*Hb), 3.76 (dd, *J* = 5.3, 14.9 Hz, 1H, C*H*CHaHb), 3.70 (s, 3H, OC*H$_3$*), 3.68 (φt, *J* = 3.7, 5.5 Hz, 1H, CHCHa*Hb*), 3.58 (s, 3H, OC*H$_3$*). ^{13}C-NMR (DMSO-d_6): *δ* 172.12 (C, C=O), 149.11 (C, *C*OCH_3), 148.04 (C, *C*OCH_3), 138.96 (C, Ar), 132.43 (C, Ar), 128.66 (2 x CH, Ar), 128.41 (2 x CH, Ar), 127.19 (CH, Ar), 119.39 (CH, Ar), 112.11 (CH, Ar), 111.13 (CH, Ar), 63.83 (CH*C*H_2OH), 56.03 (O*C*H_3), 55.63 (O*C*H_3), 55.04 (*C*HCH_2OH), 42.02 (NH*C*H_2). LRMS (ES+TOF, *m/z*): 316.15 [$C_{18}H_{21}NO_4$ + H]$^+$. HRMS (ES+TOF), *m/z.* calcd for $C_{18}H_{22}NO_4$ ([M + H]$^+$), 316.1549; found, 316.1544.

***tert*-Butyl(4-((3-hydroxy-2-phenylpropanamido)methyl)phenyl) carbamate (3k, R^1 = 4-NHBoc, R^2 = H).** Prepared from tropic acid (**1a**, R^2 = H, 0.59 g, 3.54 mmoL)) and *tert*-butyl (4-(aminomethyl)phenyl)carbamate [3] (**2i**, R^1 = 4-NHBoc). Product obtained as a yellow solid, yield 1.03 g (79 %). M.p. 118-120 °C. TLC (petroleum ether-EtOAc 1:1 v/v), R*f* = 0.2. ^{1}H NMR (DMSO-d_6): *δ* 9.25 (brs, 1H, NH), 8.45 (t, *J* = 5.9 Hz, 1H, N*H*), 7.27 (m, 6H, Ar), 7.23 (m, 1H, Ar), 7.05 (d, *J* = 8.6 Hz, 2H, Ar), 4.83 (t, *J* = 5.2 Hz, 1H, O*H*), 4.22 (dd, *J* = 6.0, 15.2 Hz, 1H, NHC*Ha*Hb), 4.15 (dd, *J* = 5.8, 15.1 Hz, 1H, NHCHa*Hb*), 3.99 (ddd, *J* = 5.6, 9.9, 29.5 Hz, 1H, CHC*Ha*Hb), 3.65 (dd, *J* = 5.5, 9.0 Hz, 1H, C*H*CHaHb), 3.53 (pentet, *J* = 5.1 Hz, 1H, CHCHa*Hb*), 1.46 (s, 9H, C(C*H$_3$*)$_3$). ^{13}C-NMR (DMSO-d_6): *δ* 172.06 (C, C=O), 153.22 (C, C=O), 138.95 (C, Ar), 138.59 (C, Ar), 133.39 (C, Ar), 129.91 (CH, Ar), 128.63 (2 x CH, Ar), 128.40 (2 x CH, Ar), 127.85 (2 x CH, Ar), 127.17 (CH, Ar), 118.37 (CH, Ar), 79.37 (*C*(CH_3)$_3$), 63.81 (*C*H_2OH), 54.96 (*C*HCH_2OH), 42.01 (NH*C*H_2), 28.60 (C(*C*H_3)$_3$). HRMS (ESI), *m/z.* calcd for $C_{21}H_{27}N_2O_4$ ([M + H]$^+$), 371.1990; found, 371.1965.

General procedure for the preparation of mesylates (4) and (11).

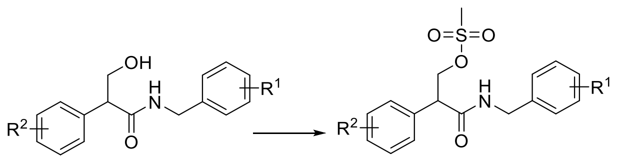

3 **4**

Where R²: a= 4-H **Where R¹: a**= 4-H **f**= 4-CF_3
b= 4-Cl **b**= 4-F **g**= 4-OCH_3
c= 4-Cl **d**= 4-di-OCH_3
d= 2,4-di-Cl **i**= 4-NHBoc
e= 4-CH_3

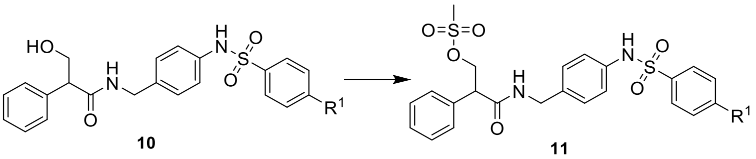

10 **11**

To an ice-cooled suspension of alcohol (**3** or **10**) (1 eq) in dry CH_2Cl_2 (2 mL/mmol), was added Et_3N (1.5 eq) followed by methane sulfonyl chloride (2.5 eq) dropwise. The reaction was stirred at 0 °C for 1 h then at room temperature overnight. The reaction was diluted with CH_2Cl_2 (10 mL/mmol), washed with 1% aqueous HCl (10 mL/mmol), water (2 x 10 mL/mmol), dried ($MgSO_4$) and evaporated under vacuum. The product was purified by petroleum ether – EtOAc gradient column chromatography.

3-(Benzylamino)-3-oxo-2-phenylpropyl methanesulfonate (4a, R^1 = R^2 = H). Prepared from *N*-benzyl-3-hydroxy-2-phenylpropanamide (**3a**) (1.21 g, 4.73 mmol) and purified by petroleum ether – EtOAc gradient column chromatography eluting with 50:50 v/v. Product obtained as a white solid, yield 0.53 g (35 %). M.p. 78-80 °C. TLC (petroleum ether-EtOAc 1:1 v/v), R*f* = 0.71. ^{1}H NMR (DMSO-d_6): *δ* 8.77 (t, *J* = 5.9 Hz, 1H, N*H*), 7.28 (m, 10H, Ar), 4.74 (t, *J* = 9.6 Hz, 1H, CHC*Ha*Hb), 4.34 (dd d, *J* = 5.7, 9.5; 6.0 Hz, 2H, CHCHa*Hb* + NHC*Ha*Hb), 4.26 (dd, *J* = 5.8, 15.3 Hz, 1H, NHCHa*Hb*), 4.03 (dd, *J* = 5.6, 9.4 Hz, 1H, C*H*CHaHb), 3.14 (s, 3H, C*H*$_3$). ^{13}C-NMR (DMSO-d_6): *δ* 170.18 (C, C=O), 139.48 (C, Ar), 136.28 (C, Ar), 129.06 (2 x CH, Ar), 128.71 (2 x CH, Ar), 128.46 (2 x CH, Ar), 128.13 (CH, Ar), 127.50 (2 x CH, Ar), 127.27 (CH, Ar), 71.14 (*C*H_2OMs), 50.84 (*C*HCH_2OMs), 42.60 (NHCH_2), 37.02 (*C*H_3). Anal. Calcd for $C_{17}H_{19}NO_4S$ (333.4014): C, 61.24%; H, 5.74%; N, 4.20%. Found: C, 61.32%; H, 5.73%; N, 4.15%.

3-((4-Fluorobenzyl)amino)-3-oxo-2-phenylpropyl methanesulfonate (4b, R^1 = 4-F, R^2 = H). Prepared from *N*-(4-fluorobenzyl)-3-hydroxy-2-phenylpropanamide (**3b**) (1.17 g, 4.27 mmol) and purified by petroleum ether – EtOAc gradient column chromatography eluting with 50:50 v/v. Product obtained as an off-white solid, yield 0.75 g (50 %). M.p. 110-112 °C. TLC (petroleum ether-EtOAc 1:1 v/v), R*f* = 0.67. ^{1}H NMR (DMSO-d_6): *δ* 8.78 (t, *J* = 5.6 Hz, 1H, N*H*), 7.38 (m, 4H, Ar), 7.32 (m, 1H, Ar), 7.22 (dd, *J* = 5.6, 8.5 Hz, 2H, Ar), 7.09 (t, *J* = 8.8 Hz, 2H, Ar), 4.74 (t, *J* = 9.6 Hz, 1H, CHC*Ha*Hb), 4.37 (dd, *J* = 5.6, 9.6 Hz, 1H, CHCHa*Hb*), 4.33 (dd, *J* = 6.0, 15.3 Hz, 1H, NHC*Ha*Hb), 4.24 (dd, *J* = 5.6, 15.2 Hz, 1H, NHCHa*Hb*), 4.03 (dd, *J* = 5.6, 9.6 Hz, 1H, C*H*CHaHb), 3.15 (s, 3H, C*H*$_3$). ^{13}C-NMR (DMSO-d_6): *δ* 170.21 (C, C=O), 162.59 and 160.66 (C, C-F), 136.23 (C, Ar), 135.69 (C, Ar), 129.53 (CH, Ar), 129.46 (CH, Ar),129.08 (2 x CH, Ar), 128.46 (2 x CH, Ar), 128.15 (CH, Ar), 115.50 (CH, Ar), 115.33 (CH, Ar), 71.13 (*C*H_2OMs), 50.86 (*C*HCH_2OMs), 41.94 (NHCH_2), 37.05 (*C*H_3). LRMS (ESI, *m/z*)*:* 352.10 [$C_{17}H_{18}FNO_4S$ + H]$^+$. HRMS (ESI), *m/z.* calcd for $C_{17}H_{19}FNO_4S$ ([M + H]$^+$), 352.1013; found, 352.1016.

3-((4-Chlorobenzyl)amino)-3-oxo-2-phenylpropyl methanesulfonate (4c, R^1 = 4-Cl, R^2 = H). Prepared from *N*-(4-chlorobenzyl)-3-hydroxy-2-phenylpropanamide (**3c**) (0.54 g, 1.86 mmol) and purified by petroleum ether – EtOAc gradient column chromatography eluting with 60:40 v/v. Product obtained as a white solid, yield 0.48 g (70 %). M.p. 110-112 °C. TLC (petroleum ether-EtOAc 1:1 v/v), R*f* = 0.73. ^{1}H NMR (DMSO-d_6): *δ* 8.84 (t, *J* = 5.9 Hz, 1H, N*H*), 7.37 (m, 4H, Ar), 7.33 (m, 3H, Ar), 7.19 (d, *J* = 8.5 Hz, 2H, Ar), 4.73 (t, *J* = 9.6 Hz, 1H, C*H*CHaHb), 4.37 (dd, *J* = 5.7, 9.5 Hz, 1H, CHC*Ha*Hb), 4.32 (dd, *J* = 6.1, 15.6 Hz, 1H, NHC*Ha*Hb), 4.24 (dd, *J* = 5.8, 15.5 Hz, 1H, NHCHa*Hb*), 4.02 (dd, *J* = 5.7, 9.7 Hz, 1H, CHCHa*Hb*), 3.15 (s, 3H, CH_3). ^{13}C-NMR (DMSO-d_6): *δ* 170.3 (C, C=O), 138.6 (C, Ar),

136.1 (C, Ar), 131.8 (C, C-Cl), 129.9 (2 x CH, Ar), 129.4 (2 x CH, Ar), 128.8 (2 x CH, Ar), 128.5 (2 x CH, Ar), 128.2 (CH, Ar), 71.1 (*C*H_2OMs), 50.8 (*C*HCH_2OMs), 41.9 (NH*C*H_2), 37.0 (CH_3). Anal. Calcd for $C_{17}H_{18}ClNO_4S{\bullet}0.1H_2O$ (369.6486): C, 55.24%; H, 4.96%; N, 3.80%. Found: C, 54.89%; H, 5.12%; N, 3.84%.

3-((4-Chlorobenzyl)amino)-2-(4-chlorophenyl)-3-oxopropyl methanesulfonate (4d, R^1 = R^2 = 4-Cl). Prepared from *N*-(4-chlorobenzyl)-2-(4-chlorophenyl)-3-hydroxypropanamide (**3d**) (1.62 g, 5 mmol) and purified by petroleum ether – EtOAc gradient column chromatography eluting with 60:40 v/v. Product obtained as a brown solid, yield 0.45 g (23 %). M.p. 106-108 °C. TLC (petroleum ether-EtOAc 2:1 v/v), R*f* = 0.27. ^{1}H NMR (DMSO-d_6): *δ* 8.83 (dd, *J* = 5.0, 10.0 Hz, 1H, N*H*), 7.43 (m, 4H, Ar), 7.34 (ddd, *J* = 2.5, 4.4, 9.1 Hz, 2H, Ar), 7.20 (d, *J* = 8.5 Hz, 2H, Ar), 4.70 (t, *J* = 9.5 Hz, 1H, C*H*CHaHb), 4.37 (dd, *J* = 6.0, 9.6 Hz, 1H, CHC*Ha*Hb), 4.32 (dd, *J* = 6.1, 15.4 Hz, 1H, NHC*Ha*Hb), 4.24 (dd, *J* = 5.8, 15.4 Hz, 1H, NHCHa*Hb*), 4.04 (dd, *J* = 6.0, 9.3 Hz, 1H, CHCHa*Hb*), 3.36 (s, 3H, C*H_3*). ^{13}C-NMR (DMSO-d_6): *δ* 169.95 (C, C=O), 138.50 (C, Ar), 135.16 (C, Ar), 132.91 (C, C-Cl), 131.86 (C, C-Cl), 130.36 (2 x CH, Ar), 129.38 (2 x CH, Ar), 129.07 (2 x CH, Ar), 128.67 (2 x CH, Ar), 70.83 (CH*C*H_2OMs), 50.13 (*C*HCH_2OMs), 42.00 (NH*C*H_2), 37.04 (*C*H_3). Anal. Calcd for $C_{17}H_{17}Cl_2NO_4S$ (402.2916): C, 50.76%; H, 4.26%; N, 3.48%. Found: C, 51.09%; H, 4.27%; N, 3.74%.

3-((2,4-Dichlorobenzyl)amino)-3-oxo-2-phenylpropyl methanesulfonate (4e, R^1 = 2,4-diCl, R^2 = H). Prepared from *N*-(2,4-dichlorobenzyl)-3-hydroxy-2-phenylpropanamide (**3e**) (1.62 g, 4.99 mmol) and purified by petroleum ether – EtOAc gradient column chromatography eluting with 70:30 v/v. Product obtained as a cream solid, yield 1.25 g (62 %). M.p. 104-106 °C. TLC (petroleum ether-EtOAc 1:1 v/v), R*f* = 0.75. ^{1}H NMR (DMSO-d_6): *δ* 8.83 (t, *J* = 5.7 Hz, 1H, N*H*), 7.59 (d, *J* = 2.1 Hz, 1H, Ar), 7.38 (m, 4H, Ar), 7.33 (m, 1H, Ar), 7.31 (dd, *J* = 2.1, 8.3 Hz, 1H, Ar), 7.20 (d, *J* = 8.4 Hz, 1H, Ar), 4.72 (t, *J* = 9.6 Hz, 1H, CHC*Ha*Hb), 4.38 (dd, *J* = 3.4, 5.6 Hz, 1H, CHCHa*Hb*), 4.35 (φt, *J* = 3.7, 5.9 Hz, 1H, NHC*Ha*Hb), 4.29 (dd, *J* = 5.7, 15.9 Hz, 1H, NHCHa*Hb*), 4.07 (dd, *J* = 5.6, 9.7 Hz, 1H, C*H*CHaHb), 3.15 (s, 3H, C*H_3*). ^{13}C-NMR (DMSO-d_6): *δ* 170.47 (C, C=O), 136.01 (C, Ar), 135.65 (C, Ar), 133.51 (C, C-Cl), 132.77 (C, C-Cl), 130.45 (CH, Ar), 129.10 (3 x CH, Ar), 128.49 (2 x CH, Ar), 128.21 (CH, Ar), 127.59 (CH, Ar), 71.06 (CH*C*H_2OMs), 50.78 (*C*HCH_2OMs), 40.25 (NH*C*H_2), 37.05 (*C*H_3). Anal. Calcd for $C_{17}H_{17}Cl_2NO_4S$ (402.2916): C, 50.76%; H, 4.26%; N, 3.48%. Found: C, 50.90%; H, 4.02%; N, 3.55%.

2-(4-Chlorophenyl)-3-((2,4-dichlorobenzyl)amino)-3-oxopropyl methanesulfonate (4f, R^1 = 2,4-diCl, R^2 = Cl). Prepared from 2-(4-chlorophenyl)-*N*-(2,4-dichlorobenzyl)-3-hydroxypropanamide (**3f**) (0.6 g, 1.67 mmoL) and purified by petroleum ether – EtOAc gradient column chromatography eluting with 60:40 v/v. Product obtained as a white solid, yield 0.58 g (79 %). M.p. 108-110 °C. TLC (petroleum ether-EtOAc 2:1 v/v), R*f* = 0.83. ^{1}H NMR (DMSO-d_6): *δ* 8.84 (t, *J* = 5.8 Hz, 1H, N*H*), 7.60 (d, *J* = 2.1 Hz, 1H, Ar), 7.43 (dd, *J* = 8.7, 18.2 Hz, 1H, Ar), 7.33 (dd, *J* = 2.1, 8.3 Hz, 1H, Ar), 7.22 (d, *J* = 8.3 Hz, 1H, Ar), 4.68 (t, *J* = 9.5 Hz, 1H, CHC*Ha*Hb), 4.37 (dd, *J* = 3.4, 6.1 Hz, 1H, CHCHa*Hb*), 4.34 (φt, *J* = 3.5, 5.8 Hz, 1H, NHC*Ha*Hb), 4.29 (dd, *J* = 5.7, 15.8 Hz, 1H, NHCHa*Hb*), 4.08 (dd, *J* = 5.9, 9.4 Hz, 1H, C*H*CHaHb), 3.15 (s, 3H, C*H_3*). ^{13}C-NMR (DMSO-d_6): *δ* 170.10 (C, C=O), 135.53 (C, Ar), 135.00 (C, Ar), 133.55 (C, C-Cl), 132.94 (C, C-Cl), 132.84 (C, C-Cl), 130.56 (CH, Ar), 130.39 (2 x CH, Ar), 129.12 (CH, Ar), 129.07 (2 x CH, Ar), 127.65 (CH, Ar), 70.79 (CH*C*H_2OMs), 50.04 (*C*HCH_2OMs), 40.49 (NH*C*H_2), 37.03 (*C*H_3). HRMS (ESI), *m/z.* calcd for $C_{17}H_{17}Cl_3NO_4S$ ($[M + H]^+$), 437.9945; found, 437.9910.

3-((4-Methylbenzyl)amino)-3-oxo-2-phenylpropyl methanesulfonate (4g, R^1 = 4-CH_3, R^2 = H). Prepared from 3-hydroxy-*N*-(4-methybenzyl)-2-phenylpropanamide (**3g**) (1.30 g, 4.84 mmol) and purified by petroleum ether – EtOAc gradient column chromatography eluting with 50:50 v/v. Product obtained as an off-white solid, yield 0.98 g (58 %). M.p. 132-134 °C. TLC (petroleum ether-EtOAc 1:1 v/v), R*f* = 0.64. ^{1}H NMR (DMSO-d_6): *δ* 8.80 (t, *J* = 5.7 Hz, 1H, N*H*), 7.35 (m, 5H, Ar), 7.06 (dd, *J* = 8.2, 13.7 Hz, 4H, Ar), 7.73 (t, *J* = 9.6 Hz, 1H, CHC*Ha*Hb), 4.35 (dd, *J* = 5.7, 9.5 Hz, 1H, CHCHa*Hb*), 4.27 (dd, *J* = 5.9, 15.1 Hz, 1H, NHC*Ha*Hb), 4.21 (dd, *J* = 5.8, 15.1 Hz, 1H, NHCHa*Hb*), 4.01 (dd, *J* = 5.6, 9.6 Hz, 1H, C*H*CHaHb), 3.14 (s, 3H, SO_2C*H_3*), 2.25 (s, 3H, C*H_3*). ^{13}C-NMR (DMSO-d_6): *δ* 170.08 (C,

C=O), 136.42 (C, Ar), 136.32 (2 x C, Ar), 129.24 (2 x CH, Ar), 129.05 (2 x CH, Ar), 128.45 (2 x CH, Ar), 128.11 (CH, Ar), 127.49 (2 x CH, Ar), 71.14 (CH*C*H$_2$OMs), 50.83 (*C*HCH$_2$OMs), 42.34 (NH*C*H$_2$), 37.02 (SO$_2$*C*H$_3$), 21.08 (O*C*H$_3$). Anal. Calcd for $C_{18}H_{21}NO_4S$ (347.4282): C, 62.23%; H, 6.09%; N, 4.03%. Found: C, 62.45%; H, 5.96%; N, 4.18%.

3-Oxo-2-phenyl-3-((4-(trifluoromethyl)benzyl)amino)propyl methanesulfonate (4h, R^1 = 4-CF$_3$, R^2 = H). Prepared from 3-hydroxy-2-phenyl-*N*-(4-(trifluoromethyl)benzyl)propanamide (**3h**) (0.43 g, 1.33 mmol) and purified by petroleum ether – EtOAc gradient column chromatography eluting with 60:40 v/v. Product obtained as a cream solid, yield 0.24 g (45 %). M.p. 104-106 ^{o}C. TLC (petroleum ether-EtOAc 1:1 v/v), R*f* = 0.60. ^{1}H NMR (DMSO-d$_6$): *δ* 8.88 (t, *J* = 6.0 Hz, 1H, N*H*), 7.62 (d, *J* = 8.2 Hz, 2H, Ar), 7.36 (m, 7H, Ar), 4.73 (t, *J* = 9.5 Hz, 1H, CHC*Ha*Hb), 4.38 (m, 3H, CHCHa*Hb* + NHC*HaHb*), 4.04 (dd, *J* = 5.7, 9.7 Hz, 1H, C*H*CHaHb), 3.15 (s, 3H, C*H$_3$*). ^{13}C-NMR (DMSO-d$_6$): *δ* 170.45 (C, C=O), 144.51 (C, Ar), 136.20 (C, Ar), 129.11 (3 x CH, Ar), 128.46 (3 x CH, Ar), 128.20 (CH, Ar), 128.10 (2 x CH, Ar), 127.83 (C, Ar), 125.56 & 125.53 (CF$_3$), 71.08 (CH*C*H$_2$OMs), 50.87 (*C*HCH$_2$OMs), 42.24 (NH*C*H$_2$), 37.02 (*C*H$_3$). ^{19}F-NMR (DMSO-d$_6$): *δ* -60.83. Anal. Calcd for $C_{18}H_{18}F_3NO_4S$ (401.3997): C, 53.86%; H, 4.52%; N, 3.49%. Found: C, 53.83%; H, 4.65%; N, 3.39%.

3-((4-Methoxybenzyl)amino)-3-oxo-2-phenylpropyl methanesulfonate (4i, R^1 = 4-OCH$_3$, R^2 = H). Prepared from 3-hydroxy-*N*-(4-methoxybenzyl)-2-phenylpropanamide (**3i**) (1.33 g, 4.65 mmol) and purified by petroleum ether – EtOAc gradient column chromatography eluting with 60:40 v/v. Product obtained as a caramel coloured solid, yield 0.73 g (43 %). M.p. 94-96 ^{o}C. TLC (petroleum ether-EtOAc 1:1 v/v), R*f* = 0.54. ^{1}H NMR (DMSO-d$_6$): *δ* 8.68 (t, *J* = 5.7 Hz, 1H, N*H*), 7.37 (m, 4H, Ar), 7.33 (t, *J* = 6.9 Hz, 1H, Ar), 7.10 (d, *J* = 8.5 Hz, 2H, Ar), 6.83 (d, *J* = 8.5 Hz, 2H, Ar), 4.73 (t, *J* = 10.0 Hz, 1H, CHC*Ha*Hb), 4.35 (dd, *J* = 5.7, 9.5 Hz, 1H, CHCHa*Hb*), 4.26 (dd, *J* = 5.9, 14.9 Hz, 1H, NHC*Ha*Hb), 4.19 (dd, *J* = 5.7, 15.0 Hz, 1H, NHCHa*Hb*), 4.01 (dd, *J* = 5.7, 9.5 Hz, 1H, C*H*CHaHb), 3.72 (s, 3H, SO$_2$C*H$_3$*), 3.14 (s, 3H, C*H$_3$*). ^{13}C-NMR (DMSO-d$_6$): *δ* 170.01 (C, C=O), 158.70 (C, *C*-OCH$_3$), 136.35 (C, Ar), 131.38 (C, Ar), 129.05 (2 x CH, Ar), 128.89 (2 x CH, Ar), 128.45 (2 x CH, Ar), 128.10 (CH, Ar), 114.13 (2 x CH, Ar), 71.17 (CH*C*H$_2$OMs), 55.52 (O*C*H$_3$), 50.82 (*C*HCH$_2$OMs), 49.07 (NH*C*H$_2$), 37.05 (SO$_2$*C*H$_3$). Anal. Calcd for $C_{18}H_{21}NO_5S \bullet 0.1H_2O$ (365.2291): C, 59.20%; H, 5.85%; N, 3.84%. Found: C, 58.90%; H, 5.89%; N, 3.58%.

3-((3,4-Dimethoxybenzyl)amino)-3-oxo-2-phenylpropyl methanesulfonate (4j, R^1 = 3,4-diOCH$_3$, R^2 = H). Prepared from *N*-(3,4-dimethoxybenzyl)-3-hydroxy-2-phenylpropanamide (**3j**) (0.99 g, 3.10 mmol) and purified by petroleum ether – EtOAc gradient column chromatography eluting with 60:40 v/v. Product obtained as a white solid, yield 0.54 g (44 %). M.p. 114-116 ^{o}C. TLC (petroleum ether-EtOAc 1:1 v/v), R*f* = 0.51. ^{1}H NMR (DMSO-d$_6$): *δ* 8.71 (t, *J* = 5.9 Hz, 1H, N*H*), 7.42 (d, *J* = 7.1 Hz, 2H, Ar), 7.37 (t, *J* = 7.4 Hz, 2H, Ar), 7.31 (t, *J* = 7.1 Hz, 1H, Ar), 6.83 (d, *J* = 8.0 Hz, 1H, Ar), 6.70 (d, *J* = 9.1 Hz, 2H, Ar), 4.75 (t, *J* = 9.6 Hz, 1H, CHC*Ha*Hb), 4.35 (dd, *J* = 5.6, 9.5 Hz, 1H, CHCHa*Hb*), 4.27 (dd, *J* = 6.1, 15.1 Hz, 1H, NHC*Ha*Hb), 4.15 (dd, *J* = 5.7, 15.0 Hz, 1H, NHCHa*Hb*), 4.02 (dd, *J* = 5.6, 9.6 Hz, 1H, C*H*CHaHb), 3.71 (s, 3H, OC*H$_3$*), 3.59 (s, 3H, OC*H$_3$*), 3.15 (s, 3H, SO$_2$C*H$_3$*). ^{13}C-NMR (DMSO-d$_6$): *δ* 170.05 (C, C=O), 149.15 (C, *C*OCH$_3$), 148.19 (C, *C*OCH$_3$), 136.40 (C, Ar), 131.97 (C, Ar), 129.08 (2 x CH, Ar), 128.45 (2 x CH, Ar), 128.10 (CH, Ar), 119.53 (CH, Ar), 112.11 (CH, Ar), 111.11 (CH, Ar), 71.14 (CH*C*H$_2$OMs), 56.02 (O*C*H$_3$), 55.63 (O*C*H$_3$), 50.88 (*C*HCH$_2$OMs), 42.22 (NH*C*H$_2$), 37.02 (SO$_2$*C*H$_3$). HRMS (ESI), *m/z.* calcd for $C_{19}H_{23}NO_6SNa$ ([M + Na]$^+$), 416.1144; found, 461.1138.

3-((4-((*tert*-Butoxycarbonyl)amino)benzyl)amino)-3-oxo-2-phenylpropyl methanesulfonate (4k, R^1 = NHBoc, R^2 = H). Prepared from *tert*-butyl (4-((3-hydroxy-2-phenylpropanamido)methyl)phenyl)carbamate (**3k**) (1.0 g, 2.72 mmol) and purified by petroleum ether – EtOAc gradient column chromatography eluting with 50:50 v/v. Product obtained as a white solid, yield 0.64 g (53 %). M.p. 118-120 ^{o}C. TLC (petroleum ether-EtOAc 1:1 v/v), R*f* = 0.63. ^{1}H NMR (DMSO-d$_6$): *δ* 9.26 (brs, 1H, N*H*), 8.69 (t, *J* = 5.9 Hz, 1H, N*H*), 7.35 (m, 7H, Ar), 7.03 (d, *J* = 8.6 Hz, 2H, Ar), 4.72 (t, *J* = 9.6 Hz, 1H, CHC*Ha*Hb), 4.35 (dd, *J* = 5.7, 9.5 Hz, 1H, CHCHa*Hb*), 4.24 (dd, *J* = 5.9, 15.1 Hz, 1H, NHC*Ha*Hb), 4.17 (dd, *J* = 5.8, 15.1 Hz, 1H, NHCHa*Hb*), 4.00 (dd, *J* = 5.1, 9.0 Hz, 1H, C*H*CHaHb), 3.14 (s, 3H, SO$_2$C*H$_3$*), 1.46

(s, 9H, C(CH_3)$_3$). ^{13}C-NMR (DMSO-d$_6$): δ 170.04 (C, C=O), 153.21 (C, C=O), 138.73 (C, Ar), 136.32 (C, Ar), 132.92 (C, Ar), 129.04 (2 x CH, Ar), 128.58 (CH, Ar), 128.45 (2 x CH, Ar), 128.10 (CH, Ar), 127.85 (2 x CH, Ar), 118.42 (CH, Ar), 79.40 (*C*(CH$_3$)$_3$), 71.15 (CH*C*H$_2$OMs), 50.82 (*C*HCH$_2$OMs), 42.15 (NH*C*H$_2$), 37.02 (*C*H$_3$), 28.60 (C(*C*H$_3$)$_3$). HRMS (ESI), *m/z.* calcd for $C_{22}H_{29}N_2O_6S$ ([M + H]$^+$), 449.1806; found, 449.1741.

3-Oxo-2-phenyl-3-((4-(phenylsulfonamido)benzyl)amino)propyl methanesulfonate (11a, R^1 = H). Prepared from 3-hydroxy-2-phenyl-*N*-(4-(phenylsulfonamido)benzyl)propanamide (**10a**) (0.33 g, 0.81 mmol) and purified by petroleum ether – EtOAc gradient column chromatography eluting with 40:60 v/v. Product obtained as a white solid, yield 0.35 g (90 %). M.p. 80-82 °C. TLC (petroleum ether-EtOAc 1:1 v/v), R*f* = 0.37. ^{1}H NMR (DMSO-d$_6$): δ 10.22 (brs, 1H, N*H*SO$_2$), 8.85 (t, *J* = 6.0 Hz, 1H, N*H*), 7.81 (d, *J* = 7.7 Hz, 3H, Ar), 7.67 (t, *J* = 6.8 Hz, 2H, Ar), 7.38 (m, 5H, Ar), 7.21 (d, *J* = 8.5 Hz, 2H, Ar), 7.11 (d, *J* = 8.5 Hz, 2H, Ar), 4.74 (t, *J* = 9.6 Hz, 1H, CHC*Ha*Hb), 4.38 (ddd, *J* = 5.9, 11.9, 16.5 Hz, 2H, NHC*Ha*Hb + CHCHa*Hb*), 4.28 (dd, *J* = 5.7, 15.8 Hz, 1H, NHCHa*Hb*), 4.04 (dd, *J* = 5.9, 8.4 Hz, 1H, C*H*CHaHb), 3.14 (s, 3H, SO$_2$C*H$_3$*). ^{13}C-NMR (DMSO-d$_6$): δ 170.41 (C, C=O), 138.86 (C, Ar), 136.15 (C, Ar), 135.05 (CH, Ar), 132.67 (C, Ar), 131.39 (2 x CH, Ar), 129.97 (2 x CH, Ar), 129.10 (2 x CH, Ar), 128.47 (2 x CH, Ar), 128.45 (2 x CH, Ar), 128.29 (2 x CH, Ar), 128.19 (CH, Ar), 71.09 (CH*C*H$_2$OMs), 50.85 (*C*HCH$_2$OMs), 42.08 (NH*C*H$_2$), 37.04 (*C*H$_3$). HRMS (ESI), *m/z.* calcd for $C_{23}H_{25}N_2O_6S_2$ ([M + H]$^+$), 489.1196; found, 489.1149.

3-Oxo-2-phenyl-3-((4-(4-fluorophenylsulfonamido)benzyl)amino)propyl methanesulfonate (11b, R^1 = 4-F). Prepared from *N*-(4-((4-fluorophenyl)sulfonamido)benzyl)-3-hydroxy-2-phenylpropanamide (**10b**) (0.34 g, 0.79 mmol) and purified by petroleum ether – EtOAc gradient column chromatography eluting with 40:60 v/v. Product obtained as a white solid, yield 0.35 g (87 %). M.p. 130-132 °C. TLC (petroleum ether-EtOAc 1:1 v/v), R*f* = 0.5. ^{1}H NMR (DMSO-d$_6$): δ 10.24 (brs, 1H, N*H*SO$_2$), 8.85 (t, *J* = 6.0 Hz, 1H, N*H*), 7.88 (dd, *J* = 5.0, 9.0 Hz, 2H, Ar), 7.52 (t, *J* = 8.8 Hz, 2H, Ar), 7.36 (m, 5H, Ar), 7.22 (d, *J* = 8.4 Hz, 2H, Ar), 7.12 (d, *J* = 8.6 Hz, 2H, Ar), 4.73 (t, *J* = 9.6 Hz, 1H, CHC*Ha*Hb), 4.38 (d dd, *J* = 6.2, 5.6, 9.4 Hz, 2H, NHC*Ha*Hb + CHCHa*Hb*), 4.28 (dd, *J* = 5.7, 15.9 Hz, 1H, NHCHa*Hb*), 4.04 (dd, *J* = 5.3, 9.4 Hz, 1H, C*H*CHaHb), 3.14 (s, 3H, SO$_2$C*H$_3$*). ^{13}C-NMR (DMSO-d$_6$): δ 170.42 (C, C=O), 166.77 (C, Ar), 142.05 (C, Ar), 136.14 (C, Ar), 135.10 (C, Ar), 132.55 (C, Ar), 131.86 (CH, Ar), 131.78 (CH, Ar), 131.39 (2 x CH, Ar), 129.11 (2 x CH, Ar), 128.47 (2 x CH, Ar), 128.35 (2 x CH, Ar), 128.19 (CH, Ar), 117.41 (CH, Ar), 117.23 (CH, Ar), 71.10 (CH*C*H$_2$OMs), 50.86 (*C*HCH$_2$OMs), 42.08 (NH*C*H$_2$), 37.03 (*C*H$_3$). HRMS (ESI), *m/z.* calcd for $C_{23}H_{23}FN_2O_6S_2Na$ ([M + Na]$^+$), 529.0874; found, 529.0883.

3-((4-((4-Chlorophenyl)sulfonamido)benzyl)amino)-3-oxo-2-phenylpropyl methanesulfonate (11c, R^1 = 4-Cl). Prepared from *N*-(4-((4-chlorophenyl)sulfonamido)benzyl)-3-hydroxy-2-phenylpropanamide (**10c**) (0.42 g, 0.94 mmol) and purified by petroleum ether – EtOAc gradient column chromatography eluting with 40:60 v/v. Product obtained as a white solid, yield 0.22 g (45 %). M.p. 158-160 °C. TLC (petroleum ether-EtOAc 1:1 v/v), R*f* = 0.46. ^{1}H NMR (DMSO-d$_6$): δ 10.29 (brs, 1H, N*H*SO$_2$), 8.85 (t, *J* = 6.0 Hz, 1H, N*H*), 7.82 (d, *J* = 8.9 Hz, 2H, Ar), 7.76 (d, *J* = 8.9 Hz, 2H, Ar), 7.37 (m, 5H, Ar), 7.23 (d, *J* = 8.4 Hz, 2H, Ar), 7.14 (d, *J* = 8.6 Hz, 2H, Ar), 4.74 (t, *J* = 9.1 Hz, 1H, CHC*Ha*Hb), 4.38 (m, 2H, NHC*Ha*Hb + CHCHa*Hb*), 4.29 (dd, *J* = 5.7, 15.8 Hz, 1H, NHCHa*Hb*), 4.05 (m, 1H, C*H*CHaHb), 3.14 (s, 3H, SO$_2$C*H$_3$*). ^{13}C-NMR (DMSO-d$_6$): δ 170.42 (C, C=O), 142.11 (C, Ar), 140.09 (C, C-Cl), 137.65 (C, Ar), 136.15 (C, Ar), 132.48 (C, Ar), 131.40 (2 x CH, Ar), 130.39 (2 x CH, Ar), 130.20 (2 x CH, Ar), 129.90 (CH, Ar), 129.10 (2 x CH, Ar), 128.47 (2 x CH, Ar), 128.18 (CH, Ar), 120.88 (CH, Ar), 71.10 (CH*C*H$_2$OMs), 50.85 (*C*HCH$_2$OMs), 42.08 (NH*C*H$_2$), 37.03 (*C*H$_3$). HRMS (ESI), *m/z.* calcd for $C_{23}H_{23}ClN_2O_6S_2Na$ ([M + Na]$^+$), 545.0584; found, 545.0574.

3-((4-((4-Methoxyphenyl)sulfonamido)benzyl)amino)-3-oxo-2-phenylpropyl methanesulfonate (11d, R^1 = 4-OCH$_3$). Prepared from 3-hydroxy-*N*-(4-((4-methoxyphenyl)sulfonamido)benzyl)-2-phenylpropanamide (**10d**) (0.55 g, 1.26 mmol) and purified by petroleum ether – EtOAc gradient column chromatography eluting with 40:60 v/v. Product obtained as a white solid, yield 0.44 g (68 %). M.p. 110-114 °C. TLC (petroleum ether-EtOAc 1:1 v/v), R*f* = 0.41. ^{1}H NMR (DMSO-d$_6$): δ 10.07 (brs, 1H, N*H*SO$_2$), 8.84 (t, *J* = 5.95 Hz, 1H, N*H*),

7.73 (d, *J* = 9.1 Hz, 2H, Ar), 7.66 (t, *J* = 9.0 Hz, 1H, Ar), 7.38 (m, 5H, Ar), 7.17 (d, *J* = 9.1 Hz, 2H, Ar), 7.04 (d, *J* = 9.0 Hz, 2H, Ar), 6.98 (d, *J* = 5.9 Hz, 2H, Ar), 4.73 (t, *J* = 9.6 Hz, 1H, CHC*Ha*Hb), 4.37 (ddd, *J* = 4.2, 9.8, 13.8 Hz, 2H, NHC*Ha*Hb + CHCHa*Hb*), 4.28 (dd, *J* = 5.7, 15.8 Hz, 1H, NHCHa*Hb*), 4.03 (d, *J* = 7.2 Hz, 1H, C*H*CHaHb), 3.89 (s, 3H, OC*H*$_3$), 3.14 (s, 3H, SO_2C*H*$_3$). ^{13}C-NMR (DMSO-d$_6$): *δ* 170.41 (C, C=O), 164.21 (C, *C*-OCH_3), 141.81 (C, Ar), 136.15 (C, Ar), 132.86 (C, Ar), 131.37 (2 x CH, Ar), 130.97 (2 x CH, Ar), 130.06 (C, Ar), 129.32 (CH, Ar), 129.10 (2 x CH, Ar), 128.47 (2 x CH, Ar), 128.23 (2 x CH, Ar), 120.30 (CH, Ar), 114.82 (CH, Ar), 71.10 (CH*C*H_2OMs), 56.40 (O*C*H_3), 50.85 (*C*HCH_2OMs), 42.01 (NH*C*H_2), 37.03 (*C*H_3). HRMS (ESI), *m/z.* calcd for $C_{24}H_{26}N_2O_7S_2Na$ ([M + Na]$^+$), 541.1079; found, 541.1068.

General procedure for the preparation of 4-((2-phenyl-3-(1*H*-1,2,4-triazol-1-yl)propanamide)methyl)benzenaminium trifluoroacetic acid salt (7) and (4-(arylsulfonamido)phenyl)methanaminium 2,2,2-trifluoroacetate derivatives (9)

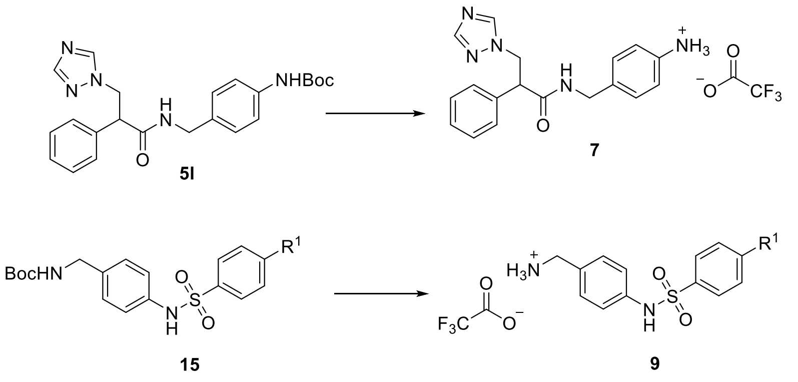

Where R^1: **a**= 4-H
b= 4-F
c= 4-Cl
d= 4-OCH_3

tert-Butyl (4-((2-phenyl-3-(1*H*-1,2,4-triazol-1-yl)propanamido)methyl)phenyl)carbamate (**5l**)/ *tert*-butyl(4-(substituted-phenylsulfonamido)benzyl)carbamate (0.48 g, 1.14 mmol) was stirred overnight with a solution of trifluoroacetic acid/CH_2Cl_2 (20 mL, 3:1 v/v). The solvent was then evaporated with further co-evaporation with EtOH (3 x 20 mL). To the resulting residue was added Et_2O (75 mL) and after stirring for 2 h the residue was collected by vacuum filtration and dried *in vacuo*.

4-((2-Phenyl-3-(1*H*-1,2,4-triazol-1-yl)propanamide)methyl)benzenaminium trifluoroacetic acid salt (7). Prepared from *tert*-butyl (4-((2-phenyl-3-(1*H*-1,2,4-triazol-1-yl)propanamido)methyl)phenyl)carbamate (0.48 g, 1.14 mmol) (**5l**). Product obtained as an orange amorphous solid, yield 0.41 g (82 %). TLC (CH_2Cl_2-MeOH 9:1 v/v), R*f* = 0.28. ^{1}H NMR (DMSO-d$_6$): *δ* 8.71 (t, *J* = 5.8 Hz, 1H, N*H*), 8.34 (s, 1H, triazole), 7.97 (s, 1H, triazole), 7.38 (d, *J* = 7.0 Hz, 2H, Ar), 7.33 (t, *J* = 7.4 Hz, 2H, Ar), 7.28 (d, *J* = 7.2 Hz, 1H, Ar), 7.05 (d, *J* = 8.0 Hz, 2H, Ar), 6.97 (d, *J* = 8.2 Hz, 2H, Ar), 4.83 (dd, *J* = 9.2, 13.5 Hz, 1H, CHC*Ha*Hb), 4.44 (dd, *J* = 6.4, 13.4 Hz, 1H, CHCHa*Hb*), 4.26 (dd, *J* = 6.4, 9.3 Hz, 2H, C*H*CHaHb + NHC*Ha*Hb), 4.08 (dd, *J* = 5.5, 15.4 Hz, 1H, NHCHa*Hb*). ^{13}C-NMR (DMSO-d$_6$): *δ* 170.70 (C, C=O), 158.77 (C, C=O), 158.50 (C, Ar), 158.22 (C, Ar), 151.92 (2 x CH, triaz), 137.49 (C, Ar), 134.17 (C, CF_3), 129.00 (2 x CH, Ar), 128.40 (3 x CH, Ar), 128.24 (2 x CH, Ar), 127.95 (CH, Ar), 121.47 (CH, Ar), 51.55 (CH*C*H_2OH), 51.14 (*C*HCH_2OH), 41.91 (NH*C*H_2). HRMS (ESI), *m/z.* calcd for $C_{18}H_{20}N_5O$ ([M + H]$^+$), 322.1702; found, 322.1662.

(4-(Phenylsulfonamido)phenyl)methanaminium trifluoroacetic acid salt [4] *(**9a**, R = H).* Prepared from *tert*-butyl(4-(phenylsulfonamido)benzyl)carbamate (1.45 g, 4.00 mmol) (R^1 = H). Product obtained as a light yellow solid, yield 1.44 g (95 %). M.p. 212-214 °C. TLC (petroleum ether-EtOAc 1:1 v/v), R*f* = 0.08. ^{1}H NMR (DMSO-d$_6$): *δ* 10.52 (brs, 1H, N*H*SO_2Ar), 8.20 (brs, 3H, N*H*$_3$), 7.80 (d, *J* = 7.3 Hz, 2H, Ar), 7.62 (t, *J* = 7.2 Hz, 1H, Ar), 7.56 (d, *J* = 7.5 Hz, 2H, Ar), 7.31 (d, *J* = 8.5 Hz, 2H, Ar), 7.13 (d, *J* = 8.5 Hz, 2H,

Ar), 3.91 (s, 2H, NH_3CH_2). ^{13}C NMR (DMSO-d_6): δ 139.86 (C, C=O), 138.37 (C, Ar), 133.48 (CH, Ar), 130.41 (2 x CH, Ar), 129.92 (C, Ar), 129.92 (2 x CH, Ar), 127.15 (2 x CH, Ar), 120.04 (2 x CH, Ar), 42.14 (NH*C*H_2).

(4-((4-Fluorophenyl)sulfonamido)phenyl)methanaminium trifluoroacetic acid salt ***(9b**, R = 4-F).* Prepared from *tert*-butyl(4-((4-fluorophenyl)sulfonamido)benzyl)carbamate (1.38 g, 4.00 mmol) (R[1] = 4-F). Product obtained as an orange solid, yield 1.39 g (97 %). M.p. 224-226 °C. TLC (petroleum ether-EtOAc 1:1 v/v), R*f* = 0.08. ^{1}H NMR (DMSO-d_6): δ 10.52 (brs, 1H, N*H*SO_2Ar), 8.18 (brs, 3H, N*H_3*), 7.86 (m, 2H, Ar), 7.41 (t, *J* = 8.8 Hz, 2H, Ar), 7.33 (d, *J* = 8.6 Hz, 2H, Ar), 7.13 (d, *J* = 8.6 Hz, 2H, Ar), 3.93 (s, 2H, NH_3C*H_2*). ^{13}C NMR (DMSO-d_6): δ 165.80 (C, C=O), 163.80 (C, *C*-F), 138.19 (C, Ar), 136.27 (C, Ar), 130.43 (2 x CH, Ar), 130.28 (CH, Ar), 130.14 (CH, Ar), 130.14 (C, Ar), 120.33 (2 x CH, Ar), 117.08 (CH, Ar), 116.90 (CH, Ar), 42.16 (NH*C*H_2). HPLC (Method B): 93 %, R_t = 0.50 min.

(4-((4-Chlorophenyl)sulfonamido)phenyl)methanaminium trifluoroacetic acid salt ***(9c**, R = 4-Cl).* Prepared from *tert*-butyl (4-((4-chlorophenyl)sulfonamido)benzyl)carbamate (1.22 g, 3.09 mmol) (R[1] = 4-Cl). Product obtained as a light orange solid, yield 1.25 g (98 %). M.p. 240-242 °C. TLC (petroleum ether-EtOAc 1:1 v/v), R*f* = 0.00. ^{1}H NMR (DMSO-d_6): δ 10.51 (s, 1H, N*H*SO_2), 8.10 (brs, 3H, $^{+}$N*H_3*CH_2), 7.78 (d, *J* = 8.8 Hz, 2H, Ar), 7.65 (d, *J* = 8.8 Hz, 2H, Ar), 7.32 (d, *J* = 8.6 Hz, 2H, Ar), 7.12 (d, *J* = 8.6 Hz, 2H, Ar), 3.92 (brs, 2H, $^{+}NH_3C$*H_2*). ^{19}F NMR (DMSO-d_6): δ -73.52. ^{13}C NMR (DMSO-d_6): δ 176.62 (C, C=O), 158.52, 158.27 (C, *C*F_3), 138.69 (C, Ar), 138.35 (C, Ar), 138.05 (C, Ar), 130.47 (2 x CH, Ar), 130.22 (C, Ar), 129.97 (2 x CH, Ar), 129.12 (2 x CH, Ar), 120.41 (2 x CH, Ar), 42.17 ($^{+}NH_3CH_2$). Anal. Calcd for $C_{15}H_{14}ClF_3N_2O_4S$ (410.7948): C, 43.86%; H, 3.43%; N, 6.82%. Found: C, 44.06%; H, 3.59%; N, 6.81%. HPLC (Method B): 98 %, R_t = 0.50 min.

(4-((4-Methoxyphenyl)sulfonamido)phenyl)methanaminium trifluoroacetic acid salt ***(9d**, R = 4-OCH$_3$).* Prepared from *tert*-butyl (4-((4-methoxyphenyl)sulfonamido)benzyl)carbamate (1.71 g, 4.35 mmol) (R[1] = 4-OCH_3). Product obtained as an orange solid, yield 1.59 g (90 %). M.p. 214-216 °C. TLC (petroleum ether-EtOAc 1:1 v/v), R*f* = 0.00. ^{1}H NMR (DMSO-d_6): δ 10.34 (s, 1H, N*H*SO_2), 8.11 (brs, 3H, $^{+}NH_3CH_2$), 7.72 (d, *J* = 9.0 Hz, 2H, Ar), 7.30 (d, *J* = 8.7 Hz, 2H, Ar), 7.12 (d, *J* = 8.6 Hz, 2H, Ar), 7.06 (d, *J* = 9.0 Hz, 2H, Ar), 3.91 (brs, 2H, $^{+}NH_3C$*H_2*), 3.80 (s, 3H, OC*H_3*). ^{19}F-NMR (DMSO-d6): δ -73.53. ^{13}C NMR (DMSO-d_6): δ 176.61 (C, *C*-OCH_3), 162.93 (C, C=O), 158.56 & 158.32 (C, *C*F_3), 138.64 (C, Ar), 131.51 (C, Ar), 130.36 (2 x CH, Ar), 129.61 (C, Ar), 129.39 (2 x CH, Ar), 119.84 (2 x CH, Ar), 114.89 (2 x CH, Ar), 56.13 (O*C*H_3), 42.19 ($^{+}NH_3$*C*H_2). HPLC (Method B): 99 %, R_t = 0.50 min.

General procedure for the preparation of *N*-(4-((4-substituedphenyl)sulfonamido)benzyl)-3-hydroxy-2-phenylpropanamides (10).

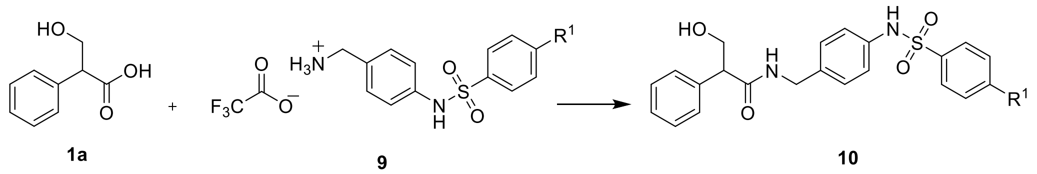

Where R[1]: **a**= 4-H
b=4-F
c=4-Cl
d=4-OCH_3

To an ice-cooled solution of tropic acid (1 eq) and HOBt (0.20 g, 1.33 mmol) in EtOAc (10 mL/mmol) was added DCC (1.1 eq) and the reaction stirred at 0 °C for 30 min. In a separate flask, a mixture of (4-(phenylsulfonamido)phenyl)methanaminium trifluoroacetic acid salt (**9**) (1 eq) in EtOAc (5 mL/mmol) and Et_3N (3 eq) was stirred at room temperature for 30 min to produce the free amine, The free amine mixture was then added to the cooled activated tropic acid-DCC mixture and the reaction stirred at room temperature overnight. Hexane (15 mL/mmol) was added to the reaction and the flask left in the freezer overnight to precipitate DCU, which was

removed by filtration and washed with EtOAc. The combined filtrates were washed with 4% aqueous HCl (2 x 30 mL), saturated aqueous $NaHCO_3$ (3 x 25 mL) and brine (3 x 30 mL). The organic layer was dried ($MgSO_4$) and evaporated under reduced pressure.

3-Hydroxy-2-phenyl-*N*-(4-(phenylsulfonamido)benzyl)propanamide (10a, R^1=H). Prepared from tropic acid (**1a**) (0.22 g, 1.33 mmol) and (4-(phenylsulfonamido)phenyl)methanaminium trifluoroacetic acid salt (**9a**) (0.5 g, 1.33 mmol). Product obtained as a white solid, yield 0.37 g (68 %). M.p. 58-60 °C. TLC (CH_2Cl_2-MeOH 95:5 v/v), R*f* = 0.67. ^{1}H NMR (DMSO-d_6): *δ* 10.21 (brs, 1H, N*H*SO_2), 8.43 (t, *J* = 5.9 Hz, 1H, N*H*), 7.74 (d, *J* = 7.1 Hz, 2H, Ar), 7.60 (t, *J* = 7.4 Hz, 1H, Ar), 7.54 (t, *J* = 7.5 Hz, 2H, Ar), 7.26 (m, 5H, Ar), 7.02 (d, *J* = 8.7 Hz, 2H, Ar), 6.97 (d, *J* = 8.7 Hz, 2H, Ar), 4.83 (t, *J* = 5.2 Hz, 1H, O*H*), 4.20 (dd, *J* = 6.1, 15.4 Hz, 1H, NHC*Ha*Hb), 4.10 (dd, *J* = 5.7, 15.4 Hz, 1H, NHCHa*Hb*), 3.97 (ddd, *J* = 5.6, 9.3, 15.5 Hz, 1H, CHC*Ha*Hb), 3.62 (dd, *J* = 5.4, 9.0 Hz, 1H, C*H*CHaHb), 3.51 (pentet, *J* = 5.1 Hz, 1H, CHCHa*Hb*). ^{13}C-NMR (DMSO-d_6): *δ* 172.14 (C, C=O), 139.96 (C, Ar), 138.82 (C, Ar), 136.61 (C, Ar), 135.72 (C, Ar), 133.31 (CH, Ar), 129.70 (2 x CH, Ar), 128.63 (2 x CH, Ar), 128.37 (2 x CH, Ar), 128.25 (2 x CH, Ar), 127.19 (CH, Ar), 127.09 (2 x CH, Ar), 120.55 (2 x CH, Ar), 63.77 (CH*C*H_2OH), 54.94 (*C*HCH_2OMs), 41.84 (NH*C*H_2). Anal. Calcd for $C_{22}H_{22}N_2O_4S$ (410.4868): C, 64.37%; H, 5.40%; N, 6.82%. Found: C, 64.40%; H, 5.72%; N, 7.22%.

***N*-(4-((4-Fluorophenyl)sulfonamido)benzyl)-3-hydroxy-2-phenylpropanamide (10b, R^1 = 4-F).** Prepared from tropic acid (**1a**) (0.21 g, 1.27 mmol) and (4-(4-fluorophenylsulfonamido)phenyl)methanaminium trifluoroacetic acid salt (**9b**) (0.5 g, 1.27 mmol). Product obtained as a white solid, yield 0.36 g (67 %). M.p. 62-64 °C. TLC (CH_2Cl_2-MeOH 95:5 v/v), R*f* = 0.64. ^{1}H NMR (DMSO-d_6): *δ* 10.23 (s, 1H, N*H*SO_2), 8.44 (t, *J* = 5.9 Hz, 1H, N*H*), 7.78 (dd, *J* = 5.2, 9.0 Hz, 2H, Ar), 7.38 (t, *J* = 8.9 Hz, 2H, Ar), 7.28 (m, 4H, Ar), 7.22 (m, 1H, Ar), 7.03 (d, *J* = 8.6 Hz, 2H, Ar), 6.97 (d, *J* = 8.6 Hz, 2H, Ar), 4.84 (t, *J* = 5.2 Hz, 1H, O*H*), 4.21 (dd, *J* = 6.1, 15.4 Hz, 1H, NHC*Ha*Hb), 4.11 (dd, *J* = 5.7, 15.4 Hz, 1H, NHCHa*Hb*), 3.97 (ddd, *J* = 5.6, 9.9, 15.9 Hz, CHC*Ha*Hb), 3.62 (dd, *J* = 5.5, 9.1 Hz, C*H*CHaHb), 3.52 (pentet, *J* = 5.1 Hz, 1H, CHCHa*Hb*). ^{13}C-NMR (DMSO-d_6): *δ* 172.17 (C, C=O), 156.71 (C, Ar), 163.71 (C, Ar), 138.80 (C, Ar), 136.45 (C, Ar), 135.94 (C, Ar), 130.19 (CH, Ar), 130.12 (CH, Ar), 128.63 (2 x CH, Ar), 128.37 (2 x CH, Ar), 128.29 (2 x CH, Ar), 127.19 (CH, Ar), 120.80 (2 x CH, Ar), 117.00 (CH, Ar), 116.82 (CH, Ar), 63.76 (CH*C*H_2OH), 54.95 (*C*HCH_2OH), 41.83 (NH*C*H_2). HRMS (ESI), *m/z.* calcd for $C_{22}H_{22}FN_2O_4S$ ($[M + H]^+$), 429.1279; found, 429.1305.

***N*-(4-((4-Chlorophenyl)sulfonamido)benzyl)-3-hydroxy-2-phenylpropanamide (10c, R^1 = 4-Cl).** Prepared from tropic acid (**1a**) (0.20 g, 1.22 mmol) and (4-(4-chlorophenylsulfonamido)phenyl)methanaminium trifluoroacetic acid salt (**9c**) (0.5 g, 1.22 mmol). Product obtained as a white solid, yield 0.45 g (84 %). M.p. 150-152 °C. TLC (CH_2Cl_2-MeOH 95:5 v/v), R*f* = 0.42. ^{1}H NMR (DMSO-d_6): *δ* 10.28 (brs, 1H, N*H*SO_2), 8.44 (t, *J* = 5.9 Hz, 1H, N*H*), 7.72 (d, *J* = 8.8 Hz, 2H, Ar), 7.62 (d, *J* = 8.8 Hz, 2H, Ar), 7.26 (m, 5H, Ar), 7.04 (d, *J* = 8.6 Hz, 2H, Ar), 6.96 (d, *J* = 8.6 Hz, 2H, Ar), 4.84 (t, *J* = 5.2 Hz, 1H, O*H*), 4.21 (dd, *J* = 6.1, 15.4 Hz, 1H, NHC*Ha*Hb), 4.11 (dd, *J* = 5.7, 15.4 Hz, 1H, NHCHa*Hb*), 4.00 (ddd, *J* = 5.6, 9.9, 14.9 Hz, 1H, CHC*Ha*Hb), 3.63 (dd, *J* = 5.4, 9.1 Hz, 1H, C*H*CHaHb), 3.52 (pentet, *J* = 5.1 Hz, 1H, CHCHa*Hb*). ^{13}C-NMR (DMSO-d_6): *δ* 172.16 (C, C=O), 138.81 (C, Ar), 138.78 (C, C-Cl), 138.20 (C, Ar), 136.27 (C, Ar), 136.09 (C, Ar), 129.89 (2 x CH, Ar), 129.06 (2 x CH, Ar), 128.63 (2 x CH, Ar), 128.38 (2 x CH, Ar), 128.34 (2 x CH, Ar), 127.19 (CH, Ar), 120.89 (2 x CH, Ar), 63.77 (CH*C*H_2OH), 54.95 (*C*HCH_2OH), 41.84 (NH*C*H_2). HRMS (ESI), *m/z.* calcd for $C_{22}H_{21}ClN_2O_4SNa$ ($[M + Na]^+$), 467.0809; found, 467.0799.

***N*-(4-((4-Methoxyphenyl)sulfonamido)benzyl)-3-hydroxy-2-phenylpropanamide (10d, R^1 = 4-OCH_3).** Prepared from tropic acid (**1a**) (0.20 g, 1.22 mmol) and (4-(4-methoxyphenylsulfonamido)phenyl)methanaminium trifluoroacetic acid salt (**9d**) (0.5 g, 1.22 mmol). Product obtained as an off-white solid, yield 0.47 g (87 %). M.p. 126-128 °C. TLC (CH_2Cl_2-MeOH 95:5 v/v), R*f* = 0.5. ^{1}H NMR (DMSO-d_6): *δ* 10.06 (brs, 1H, N*H*SO_2), 8.43 (t, *J* = 5.9 Hz, 1H, N*H*), 7.67 (d, *J* = 9.0 Hz, 2H, Ar), 7.26 (m, 5H, Ar), 7.05 (d, *J* = 9.0 Hz, 2H, Ar), 7.02 (d, *J* = 8.7 Hz, 2H, Ar), 6.96 (d, *J* = 8.7 Hz, 2H, Ar), 4.84 (t, *J* = 5.2 Hz, 1H, O*H*), 4.20 (dd, *J* = 6.1, 15.4 Hz, 1H, NHC*Ha*Hb), 4.10 (dd, *J* = 5.7, 15.4 Hz, 1H, NHCHa*Hb*), 3.97 (ddd, *J* = 5.7, 9.9, 14.9 Hz, 1H, CHC*Ha*Hb), 3.79 (s, 3H, OC*H_3*), 3.62 (dd, *J* = 5.4, 9.1 Hz,

1H, C*H*CHaHb), 3.52 (pentet, *J* = 5.1 Hz, 1H, CHCHa*Hb*). ^{13}C-NMR (DMSO-d$_6$): *δ* 172.15 (C, C=O), 162.82 (C, *C*-OCH$_3$), 138.82 (C, Ar), 136.87 (C, Ar), 135.45 (C, Ar), 131.60 (C, Ar), 129.31 (2 x CH, Ar), 128.63 (2 x CH, Ar), 128.37 (2 x CH, Ar), 128.20 (2 x CH, Ar), 127.19 (CH, Ar), 120.31 (2 x CH, Ar), 114.82 (2 x CH, Ar), 63.77 (CH*C*H$_2$OH), 56.08 (O*C*H$_3$), 54.94 (*C*HCH$_2$OH), 41.85 (NH*C*H$_2$). HRMS (ESI), *m/z.* calcd for $C_{23}H_{24}N_2O_5SNa$ ([M + Na]$^+$), 463.1304; found, 463.1295.

General procedure for preparation of the Boc protected *tert*-butyl (4-(substituted phenylsulfonamido)benzyl)carbamate (15) precursor of 9

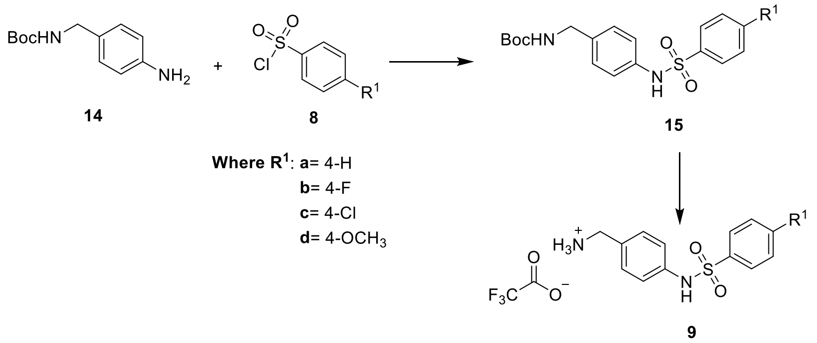

To an ice-cooled yellow solution of *tert*-butyl (4-aminobenzyl)carbamate (**14**) [4] (1 mmoL) in dry pyridine (10 mL/mmoL) was added benzenesulfonyl chloride derivative (**8**) (1.2 mmoL) portion wise and the reaction mixture was stirred at room temperature for 2 h, then pyridine removed under vacuum. The resulting syrup was diluted in CH_2Cl_2 (100 mL), washed with 0.5 M aqueous HCl (50 mL), H_2O (50 mL), dried (MgSO4) then concentrated under vacuum.

***tert*-Butyl (4-(phenylsulfonamido)benzyl)carbamate** (**15a**, R = 4-H) [1]

Prepared from benzenesulfonyl chloride (**8a**, R^1= 4-H) (0.69 mL, 5.4 mmoL). Product obtained as a white solid, yield 1.51 g (92 %) after purification with column chromatography eluting with petroleum ether – EtOAc 50:50 v/v. M.p. 160-162 °C. TLC (petroleum ether-EtOAc 3:2 v/v), R*f* = 0.53. 1H NMR (DMSO-d6): δ 10.21 (s, 1H, NHSO$_2$Ar), 7.70 (d, *J* = 7.4 Hz, 2H, Ar), 7.60 (t, *J* = 7.3 Hz, 1H, Ar), 7.54 (m, 2H, Ar), 7.27 (t, *J* = 6.3 Hz, 1H, N*H*CH$_2$), 7.08 (d, *J* = 8.5 Hz, 2H, Ar), 7.02 (d, *J* = 8.5 Hz, 2H, Ar), 4.01 (d, *J* = 6.1 Hz, 2H, NHC*H$_2$*), 1.37 (s, 9H, C(C*H$_3$*)$_3$).

***tert*-Butyl (4-((4-fluorophenyl)sulfonamido)benzyl)carbamate** (**15b**, R^1 = 4-F)

Prepared from 4-fluorobenzenesulfonyl chloride (**8b**, R^1= 4-F, 1.05g, 5.4 mmoL). Product obtained as a light orange solid, yield 1.49 g (87 %) which was washed with Et$_2$O (3 x 50 mL). M.p. 174-176 °C. TLC (petroleum ether-EtOAc 3:2 v/v), R*f* = 0.8. ^{1}H NMR (DMSO-d$_6$): δ 10.23 (brs, 1H, N*H*SO$_2$Ar), 7.80 (dd, *J* = 5.0, 10.0 Hz, 2H, Ar), 7.38 (t, *J* = 10.0 Hz, 2H, Ar), 7.28 (dd, *J* = 10.0, 5.0 Hz, 1H, N*H*CH$_2$), 7.10 (d, *J* = 5.0 Hz, 2H, Ar), 7.03 (d, *J* = 5.0 Hz, 2H, Ar), 4.02 (d, *J* = 5.0 Hz, 2H, NHC*H$_2$*), 1.38 (s, 9H, C(C*H$_3$*)$_3$). ^{13}C NMR (DMSO-d$_6$): δ 165.73 (C, *C*-F), 163.73 (C, C=O), 156.22 (C, Ar), 136.73 (C, Ar), 136.40 (C, Ar), 130.22 (CH, Ar), 130.14 (CH, Ar), 128.23 (2 x CH, Ar), 120.93 (2 x CH, Ar), 116.99 (CH, Ar), 116.81 (CH, Ar), 78.25 (C, *C*(CH$_3$)$_3$), 43.24 (NH*C*H$_2$), 28.68 (C(*C*H$_3$)$_3$). Anal. Calcd for $C_{18}H_{21}FN_2O_4S$•0.1H_2O (382.23482): C, 56.56%; H, 5.59%; N, 7.33%. Found: C, 56.18%; H, 5.77%; N, 7.07%.

***tert*-Butyl (4-((4-chlorophenyl)sulfonamido)benzyl)carbamate** (**15c**, R^1 = 4-Cl)

Prepared from 4-chlorobenzenesulfonyl chloride (**8c**, R^1= 4-Cl, 1.14, 5.4 mmoL). Product obtained as an orange solid, which was washed with Et$_2$O (3 x 50 mL), yield 1.27 g (71 %). M.p. 136-138 °C. TLC (petroleum ether-EtOAc 3:2 v/v), R*f* = 0.69. ^{1}H NMR (DMSO-

d_6): δ 10.29 (brs, 1H, N*H*SO_2), 7.73 (d, *J* = 8.7 Hz, 2H, Ar), 7.62 (d, *J* = 8.6 Hz, 2H, Ar), 7.28 (t, *J* = 6.1 Hz, 1H, N*H*CH_2), 7.10 (d, *J* = 8.7 Hz, 2H, Ar), 7.02 (d, *J* = 8.5 Hz, 2H, Ar), 4.01 (d, *J* = 6.1 Hz, 2H, NHCH_2), 1.37 (s, 9H, C(CH_3)$_3$). ^{13}C NMR (DMSO-d_6): δ 156.21 (C, C=O), 138.81 (C, Ar), 138.19 (C, *C*-Cl), 136.83 (C, Ar), 136.25 (C, Ar), 129.88 (2 x CH, Ar), 129.07 (2 x CH, Ar), 128.26 (2 x CH, Ar), 121.03 (2 x CH, Ar), 78.26 (*C*(CH_3)$_3$), 43.24 (NH*C*H_2), 28.69 (C(*C*H_3)$_3$). Anal. Calcd for $C_{18}H_{21}ClN_2O_4S$ (396.8879): C, 54.47; H, 5.33; N, 7.05 %. Found: C, 54.74; H, 5.37; N, 7.10 %. HRMS (ESI) *m/z*, calcd for $C_{18}H_{21}ClN_2O_4SNa$ ([M + Na]$^+$), 419.0803; found, 419.0809.

***tert*-Butyl (4-((4-methoxyphenyl)sulfonamido)benzyl)carbamate** (**15d**, R^1 = 4-OCH_3)

Prepared from 4-methoxybenzenesulfonyl chloride (**8d**, R^1= 4-OCH_3, 1.12, 5.4 mmoL). Product obtained as a semi-orange solid, yield 1.75 g (99 %). TLC (petroleum ether-EtOAc 3:2 v/v), R*f* = 0.41. ^{1}H NMR (DMSO-d_6): δ 10.07 (brs, 1H, N*H*SO_2), 7.68 (d, *J* = 8.9 Hz, 2H, Ar), 7.27 (t, *J* = 6.0 Hz, 1H, N*H*), 7.04 (m, 6H, Ar), 4.00 (d, *J* = 6.1 Hz, 2H, NHCH_2), 3.79 (s, 3H, OCH_3), 1.37 (s, 9H, C(CH_3)$_3$). ^{13}C NMR (DMSO-d_6): δ 162.82 (C, *C*-OCH_3), 156.20 (C, C=O), 136.85 (C, Ar), 136.21 (C, Ar), 131.64 (C, Ar), 129.32 (2 x CH, Ar), 128.68 (2 x CH, Ar), 120.44 (2 x CH, Ar), 114.81 (2 x CH, Ar), 78.23 (*C*(CH_3)$_3$), 56.06 (O*C*H_3), 43.24 (NH*C*H_2), 28.69 (C(*C*H_3)$_3$). HRMS (ESI) *m/z*, calcd for $C_{19}H_{24}N_2O_5SNa$ ([M + Na]$^+$), 415.1298; found, 415.1311.

HPLC Traces

5b

<Chromatogram>

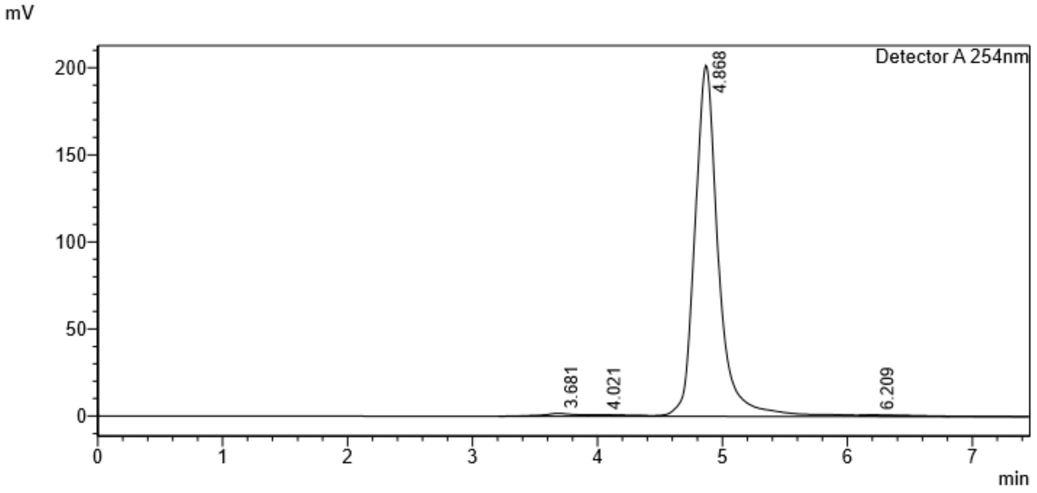

<Peak Table>

Detector A 254nm

Peak#	Ret. Time	Area	Height	Area%
1	3.681	29950	1643	1.098
2	4.021	21688	906	0.795
3	4.868	2672708	201565	98.018
4	6.209	2400	124	0.088
Total		2726747	204238	100.000

5c

<Chromatogram>

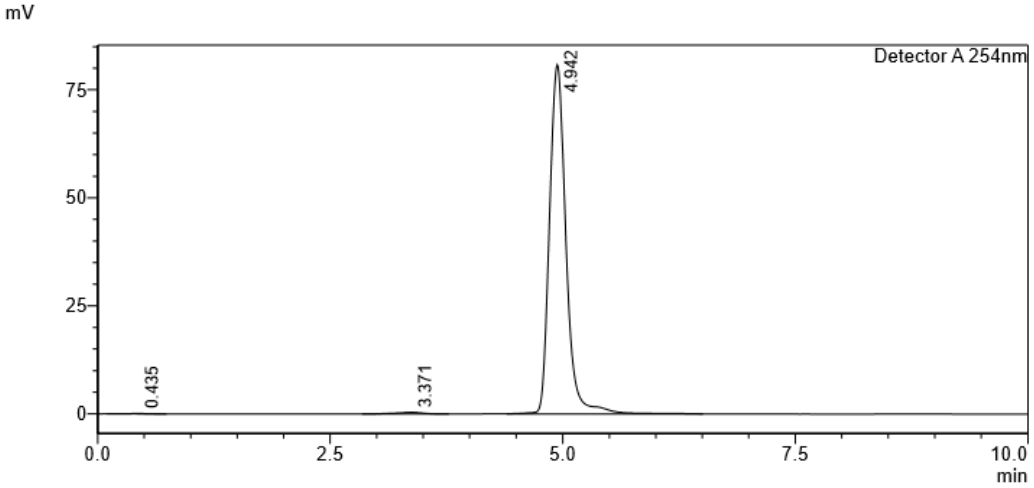

<Peak Table>

Detector A 254nm

Peak#	Ret. Time	Area	Height	Area%
1	0.435	1408	85	0.140
2	3.371	8641	399	0.860
3	4.942	994255	80836	98.999
Total		1004305	81321	100.000

5d

<Chromatogram>

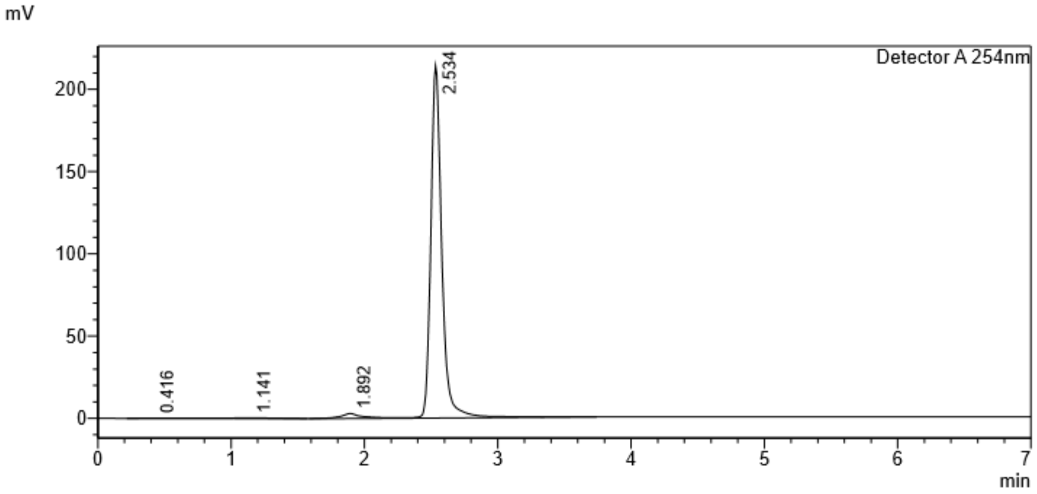

<Peak Table>

Detector A 254nm

Peak#	Ret. Time	Area	Height	Area%
1	0.416	2787	108	0.219
2	1.141	10130	349	0.796
3	1.892	34576	3008	2.718
4	2.534	1224571	214087	96.266
Total		1272063	217552	100.000

5e

<Chromatogram>

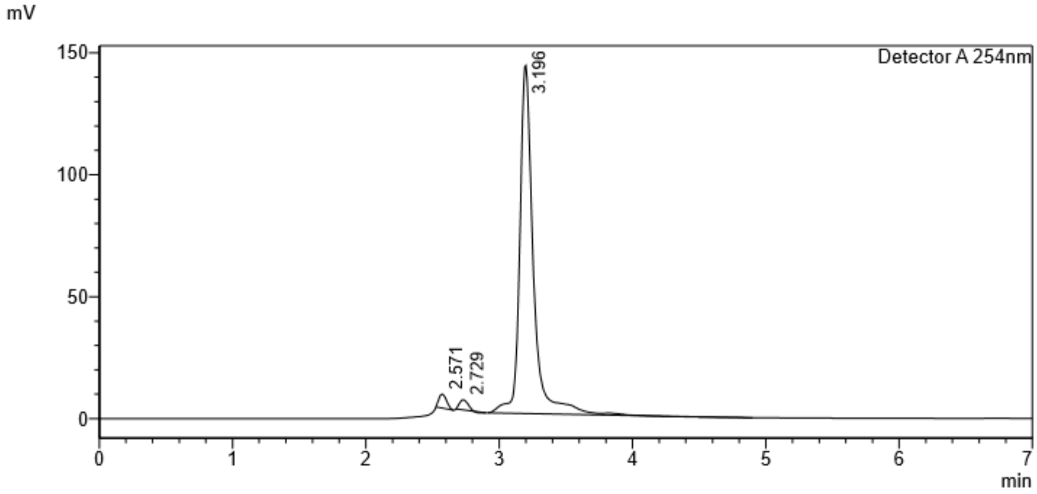

<Peak Table>

Detector A 254nm

Peak#	Ret. Time	Area	Height	Area%
1	2.571	22642	5674	2.162
2	2.729	17109	4104	1.634
3	3.196	1007336	142651	96.204
Total		1047087	152429	100.000

5f

<Chromatogram>

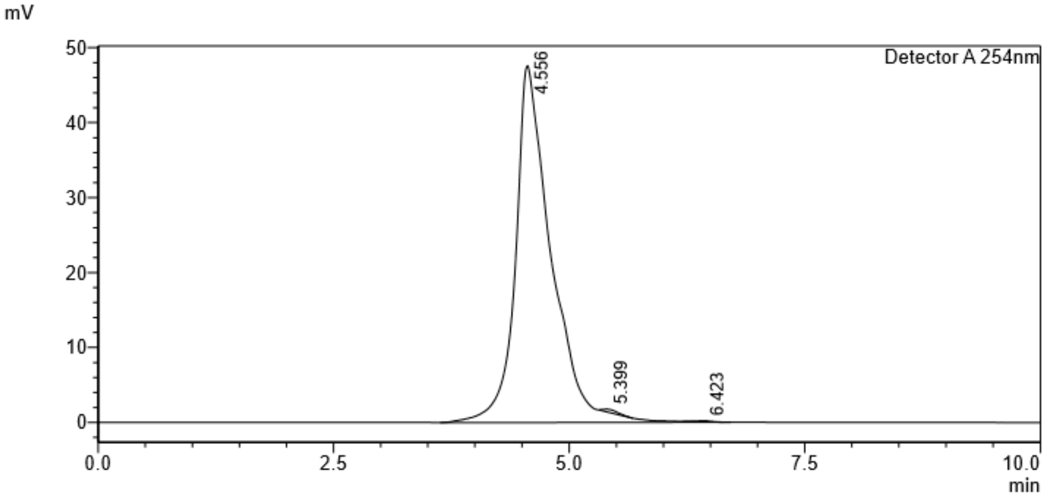

<Peak Table>

Detector A 254nm

Peak#	Ret. Time	Area	Height	Area%
1	4.556	1252926	47568	99.575
2	5.399	3867	335	0.307
3	6.423	1474	130	0.117
Total		1258267	48032	100.000

5g

<Chromatogram>

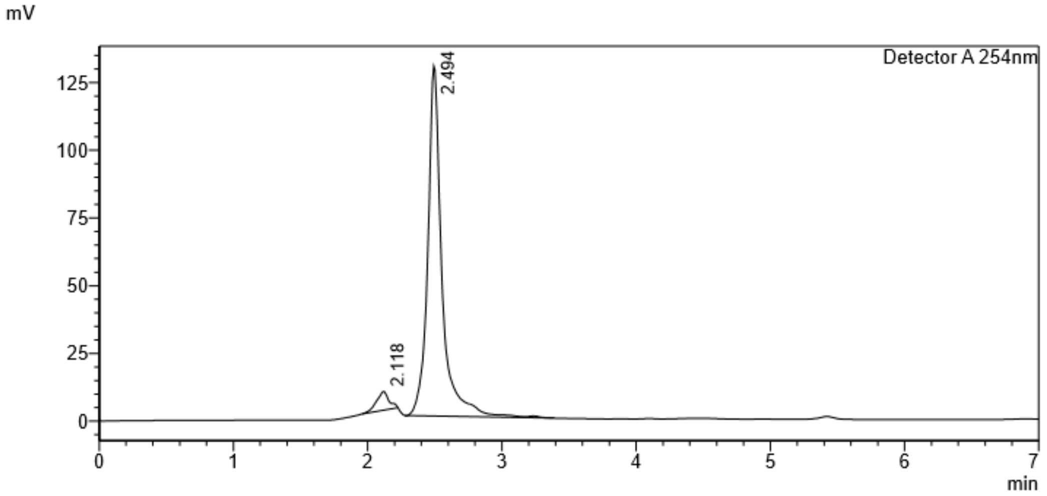

<Peak Table>

Detector A 254nm

Peak#	Ret. Time	Area	Height	Area%
1	2.118	45015	6907	4.520
2	2.494	950788	129215	95.480
Total		995803	136123	100.000

5h

<Chromatogram>

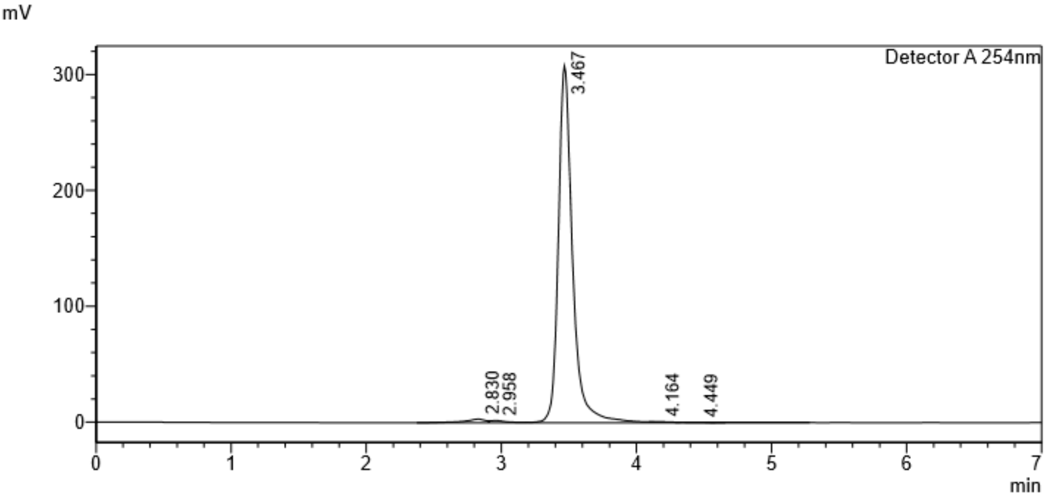

<Peak Table>

Detector A 254nm

Peak#	Ret. Time	Area	Height	Area%
1	2.830	34840	3177	1.500
2	2.958	16472	1932	0.709
3	3.467	2269279	307846	97.670
4	4.164	1578	247	0.068
5	4.449	1237	195	0.053
Total		2323407	313398	100.000

5i

<Chromatogram>

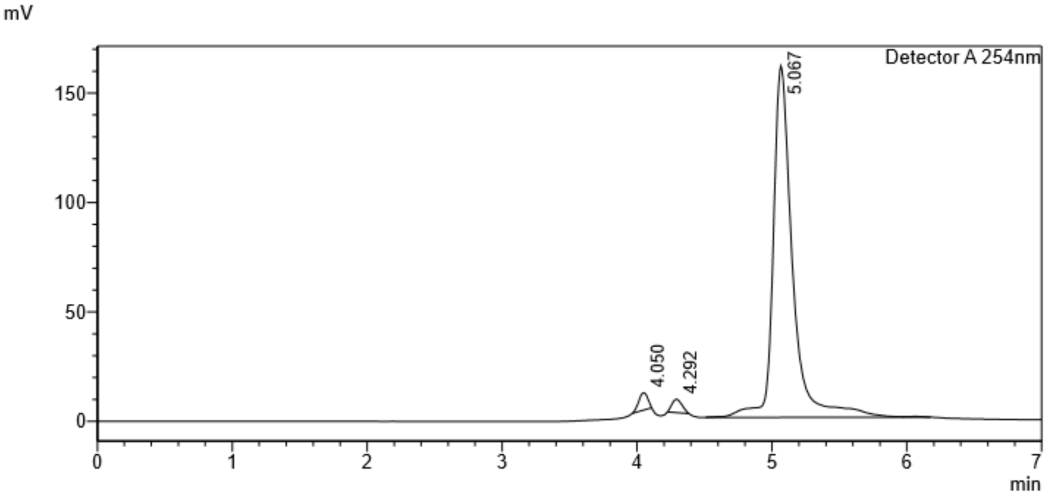

<Peak Table>

Detector A 254nm

Peak#	Ret. Time	Area	Height	Area%
1	4.050	37150	7965	2.210
2	4.292	31591	6111	1.879
3	5.067	1612349	160452	95.911
Total		1681089	174528	100.000

5j

<Chromatogram>

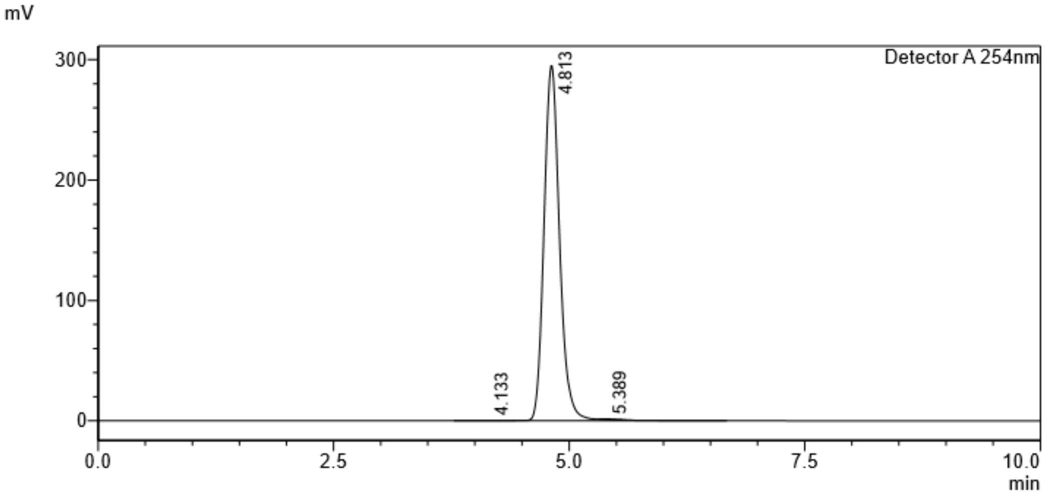

<Peak Table>

Detector A 254nm

Peak#	Ret. Time	Area	Height	Area%
1	4.133	1940	87	0.057
2	4.813	3421870	294822	99.866
3	5.389	2664	200	0.078
Total		3426474	295109	100.000

5k

<Chromatogram>

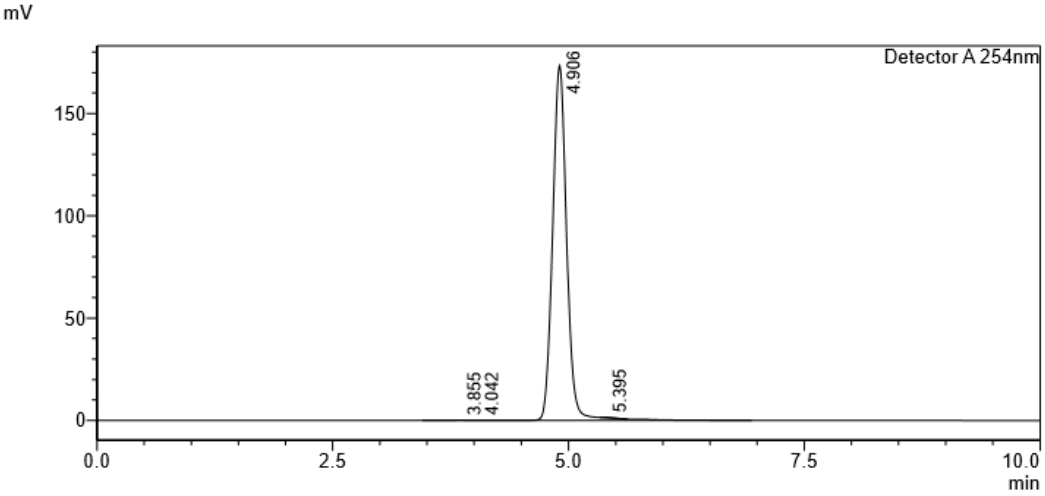

<Peak Table>

Detector A 254nm

Peak#	Ret. Time	Area	Height	Area%
1	3.855	1776	103	0.098
2	4.042	1393	79	0.077
3	4.906	1806695	173361	99.694
4	5.395	2370	219	0.131
Total		1812234	173762	100.000

5l

<Chromatogram>

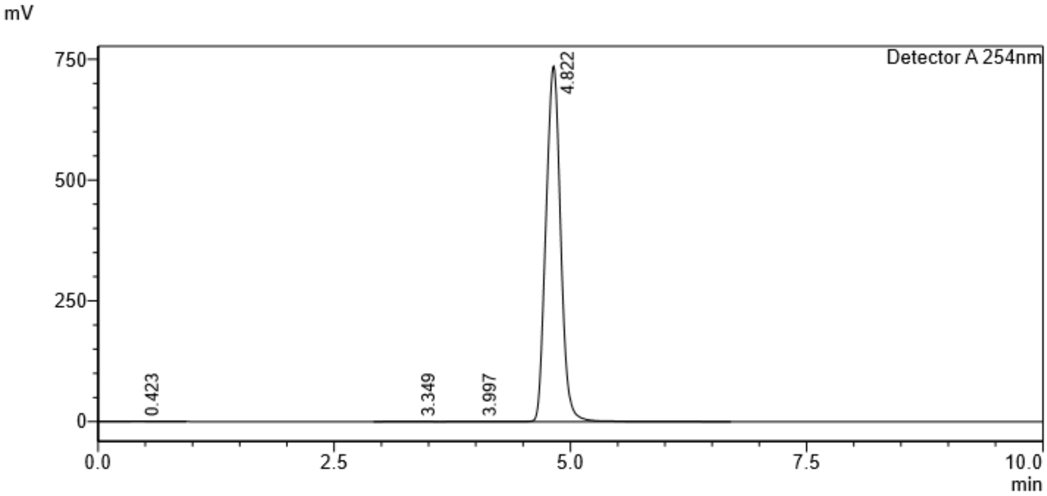

<Peak Table>

Detector A 254nm

Peak#	Ret. Time	Area	Height	Area%
1	0.423	12195	665	0.152
2	3.349	2824	136	0.035
3	3.997	9927	306	0.123
4	4.822	8021121	736045	99.690
Total		8046067	737153	100.000

12a

<Chromatogram>

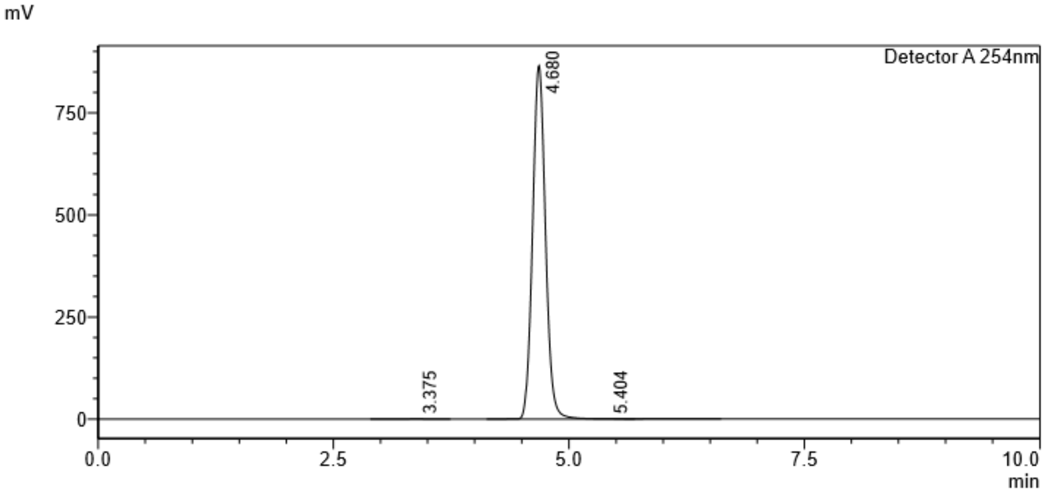

<Peak Table>

Detector A 254nm

Peak#	Ret. Time	Area	Height	Area%
1	3.375	2676	121	0.032
2	4.680	8309523	865789	99.894
3	5.404	6167	459	0.074
Total		8318367	866369	100.000

12b

<Chromatogram>

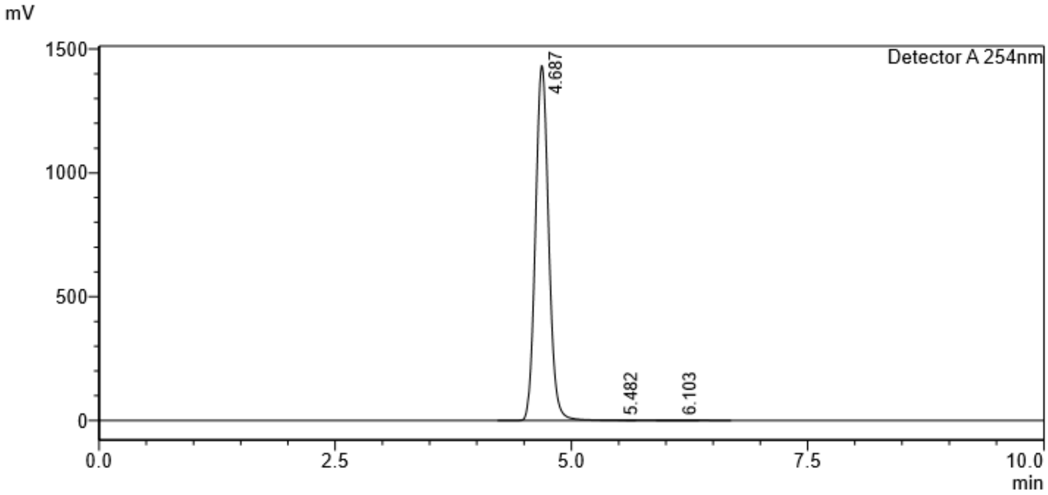

<Peak Table>

Detector A 254nm

Peak#	Ret. Time	Area	Height	Area%
1	4.687	14038058	1431887	99.903
2	5.482	11630	1082	0.083
3	6.103	1962	188	0.014
Total		14051650	1433156	100.000

12c

<Chromatogram>

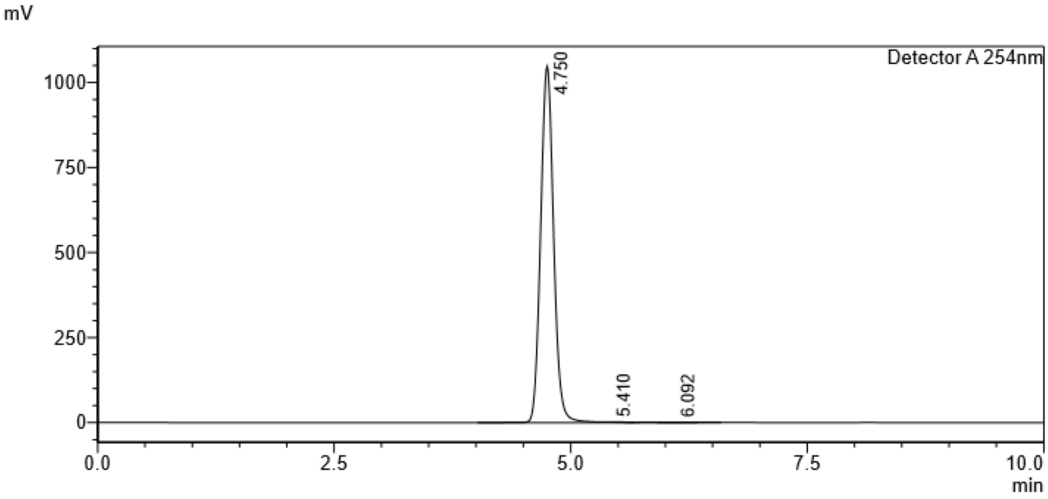

<Peak Table>

Detector A 254nm

Peak#	Ret. Time	Area	Height	Area%
1	4.750	10089207	1047789	99.905
2	5.410	7198	438	0.071
3	6.092	2416	229	0.024
Total		10098821	1048456	100.000

12d

<Chromatogram>

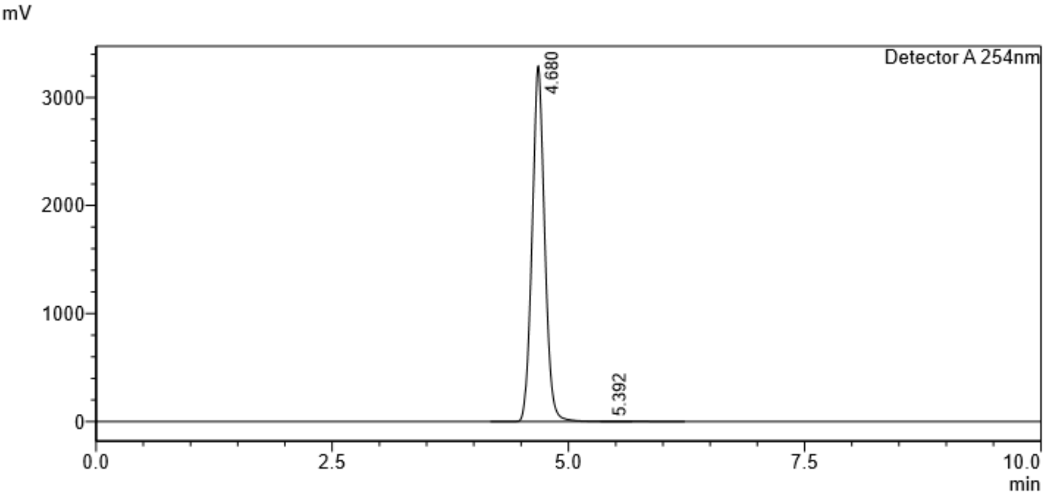

<Peak Table>

Detector A 254nm

Peak#	Ret. Time	Area	Height	Area%
1	4.680	30562984	3290856	99.990
2	5.392	2962	171	0.010
Total		30565946	3291027	100.000

References

[1] V. Karaluka, R.M. Lanigan, P.M. Murray, M. Badland, T.D. Sheppard, *Org. Biomol. Chem.* **2015**, *13*, 10888-10894.

[2] M.D. Andrews, S.K. Bagal, K.R. Gibson, K. Omoto, T. Ryckmans, S.E. Skerratt, P.A. Stupple, **2012**, WO 2012137089 A1.

[3] V. Perron, S. Abbott, N. Moreau, D. Lee, C. Penney, B. Zacharie, *Synthesis* **2009**, *2*, 283-289.

[4] J. Lee, J. Lee, M. Kang, M. Shin, J-M. Kim, S-U. Kang, J-O. Lim, H-K. Choi, Y-G. Suh, H-G. Park, U. Oh, H-D. Kim, Y-H. Park, H-J. Ha, Y-H. Kim, A. Toth, Y. Wang, R. Tran, L.V. Pearce, D.J. Lundberg, P.M. Blumberg, *J. Med. Chem.* **2003**, *46*, 3116-3126.
